# ValTrendsDB: bringing Protein Data Bank validation information closer to the user

**DOI:** 10.1093/bioinformatics/btz532

**Published:** 2019-07-02

**Authors:** Vladimír Horský, Veronika Bendová, Dominik Toušek, Jaroslav Koča, Radka Svobodová

**Affiliations:** 1 National Centre for Biomolecular Research, Faculty of Science, Masaryk University, Brno, Czech Republic; 2 CEITEC – Central European Institute of Technology, Masaryk University, Brno, Czech Republic; 3 Institute of Mathematics and Statistics, Faculty of Science, Masaryk University, Brno, Czech Republic

## Abstract

**Summary:**

Structures in PDB tend to contain errors. This is a very serious issue for authors that rely on such potentially problematic data. The community of structural biologists develops validation methods as countermeasures, which are also included in the PDB deposition system. But how are these validation efforts influencing the structure quality of subsequently published data? Which quality aspects are improving, and which remain problematic? We developed ValTrendsDB, a database that provides the results of an extensive exploratory analysis of relationships between quality criteria, size and metadata of biomacromolecules. Key input data are sourced from PDB. The discovered trends are presented via precomputed information-rich plots. ValTrendsDB also supports the visualization of a set of user-defined structures on top of general quality trends. Therefore, ValTrendsDB enables users to see the quality of structures published by selected author, laboratory or journal, discover quality outliers, etc. ValTrendsDB is updated weekly.

**Availability and implementation:**

Freely accessible at http://ncbr.muni.cz/ValTrendsDB. The web interface was implemented in JavaScript. The database was implemented in C++.

**Supplementary information:**

Supplementary data are available at *Bioinformatics* online.

## 1 Introduction

Biomacromolecular structural data are key results of modern life sciences. Their importance is reinforced by 13 Nobel prizes that were awarded for research on these data (ebi.ac.uk/pdbe/docs/nobel/nobels.html). Most structures of biomacromolecules and their ligands are accessible via the Protein Data Bank (PDB) database (Burley *et al.*, 2018). However, some structures were found to contain serious errors (Rupp, 2012). This discovery showed the importance of the validation of biomacromolecular complexes. The first validation approaches were focused on the geometric properties of standard biomacromolecular residues (i.e. amino acids, nucleotides) (Chen *et al.*, 2010). This validation approach was later extended to validate ligands (Bruno *et al.*, 2004). A step forward in validation was the release of PDB validation reports (Gore *et al.*, 2017) for almost every structure.

A major question is how the validation efforts of the scientific community are influencing the quality of structural data. Our cooperation with the Protein Data Bank in Europe (PDBe) motivated us to ask broader questions: How is the quality of biomacromolecular complexes changing over time? What factors are influencing it? To suggest answers to these questions, we carried out an exploratory analysis of trends between quality criteria, size (e.g. atom count) and the metadata of biomacromolecules (e.g. year of release). Its results can be explored in the novel ValTrendsDB database.

## 2 Database construction

ValTrendsDB is a database that presents the results of the factor pair relationship analysis. A factor represents a property or a validation metric. In the current version of ValTrendsDB, users can view trends between nearly 1700 pairs of factors that are sorted into groups. Descriptions of every factor and dataset version can be found in the Supplementary Data.

Input data for the analysis came from two sources. The PDB database provided biomacromolecular structures, as well as their validation results. This information was obtained for each structure from the mmCIF file, from the validation report, from queries to the PDBe REST API, and from the Chemical Component Dictionary, which is a database of ligands found in PDB entries (Westbrook *et al.*, 2015). Additional ligand quality information was acquired from the ValidatorDB database (Sehnal *et al.*, 2015). All PDB entries were included, regardless of their refinement method.

All the gathered data were processed statistically. Values of factors in pairs were sorted into nonequidistant intervals, which were then represented by an arithmetic mean. This workflow was designed to deal with dataset issues, e.g. the skewness of value distribution, while preserving the interpretability of factor pair plots. Spearman’s rank correlation coefficients were used to assess the factor pair relationships. Details of the workflow can be found in the Supplementary Data.

## 3 Functionality

The ValTrendsDB database brings the current state of biomacromolecular structure quality, along with its progress, trends and issues, closer to its users. They can view plots of each trend in the relationship of factor pairs. An example of such a plot is depicted in Supplementary Figure S1 on page 8 in the Supplementary Data.

Users can highlight data points representing their own set of PDB entries on top of global trends. With this functionality, they can pinpoint qualitative outliers, as well as view trends in the quality and properties of a set of structures that are of interest to them. It is suitable for the visualization and comparison of trends among, e.g. structures of a journal, protein family, experimental method, or structures from an author.

## 4 Discussion

The analysis identified several interesting quality-related trends. All of these trends are shown in much greater detail in the Supplementary Data. Namely, we found that the quality of geometry of biomacromolecular structures in PDB is steadily increasing. However, the same cannot be said in general for the quality of agreement between structure models and their source electron density, as well as for all quality aspects of ligand models. In these two cases, some quality factors are showing an improvement over time, while others are not.

It is not surprising that nearly all size factors show a trend of increasing size of biomacromolecular complexes over time, while a number of them show strong relationships with quality factors. Similarly, several quality factors exhibit strong relationships with the resolution of X-ray structures. Plots that show the discussed trends are included in the Supplementary Data.

ValTrendsDB is unique because of the breadth of the analysis whose results it presents. No other similar published analysis contains as many factors, as many data points, or is updated automatically on a weekly basis. That is why another publication that is being worked on will focus on the analysis itself.

The European Bioinformatics Institute, which maintains the PDBe, plans to integrate selected data and some of the plots from ValTrendsDB into their new validation pages. Specifically, PDBe will use these data to plot charts that will show the relative quality of an entry, as well as embed a few plots that show database-wide trends. The full functionality of ValTrendsDB will, however, remain solely on its website.

## Supplementary Material

btz532_Supplementary_Data
