# Supplementary material for: ValTrendsDB: bringing Protein Data Bank validation information closer to the user
Source: Bioinformatics. 2019 Jul 2;35(24):5389–90. doi: 10.1093/bioinformatics/btz532 (PMC6954638; doi:10.1093/bioinformatics/btz532)
Supplement: btz532_Supplementary_Data [file btz532_supplementary_data.pdf]

Supplementary data

Contents

Description of information, stored in ValTrendsDB 2
Description of factors 2
Description of the statistical workflow 4
A note on dataset versions and manual statistical analysis workflow 4
Introduction and challenges 4
Analysis of relationship between a factor pair 4
Relationship visualization design in ValTrendsDB 6
Definition of Spearman's rank correlation coefficient 7
Table S2 8
Plots of discussed trends 11
Geometry quality of biomacromolecular structures in time 11
Quality of agreement between biomacromolecule structure models and their source electron density in time 15
Geometry quality of ligands in time 18
Quality of agreement between ligand models and their source electron density in time 20
Trends of size factors in time 22
Relationships between biomacromolecule structure quality factors and the structure resolution factor 24
Relationships between ligand quality factors and the structure resolution factor 27
Relationships between biomacromolecule structure quality factors and size factors 29
Relationships between ligand quality factors and size factors 36
Complete listing of factors and factor groups 44
Structure metadata factors 44
Structure quality factors 47
Ligand quality factors 49
Version overview 52
References 52

Description of information, stored in ValTrendsDB

Description of factors

ValTrendsDB is a database that contains quality criteria, size and metadata of structures of biomacromolecular complexes, stored in the PDB database. For simplification, we denote these criteria and properties by the overall term "factors". Specifically, a factor represents a particular value of one property or quality criterion of a structure (i.e., of a PDB entry). Factors can be classified as either metadata factors, biomacromolecule quality factors, or ligand quality factors. The database contains 88 factors in total throughout all versions of the dataset, while the most recent dataset contains 84 factors. To simplify orientation within factors, we introduce groups of factors that gather similar factors (e.g., the *average ligand size* factor group). An exhaustive listing of all factors and their groups is included in the relevant section further down this Supplementary data document.

Metadata factors have been computed during data acquisition for ValTrendsDB with the aim of representing the various properties of biomacromolecular complexes, which are not related to quality, in a manner that is suitable for statistical processing. They include basal factors such as year of release of a PDB entry and its resolution. Additionally, several other properties whose relationship to quality is of interest to the research community have been computationally transformed into factors, e.g., the total number of chiral atoms in ligands, the ratio of single bonds in ligands.

The most populous factor groups among metadata factors, however, are those that quantify the size of asymmetric units of complexes (or their parts) in various ways, namely molecular weight, atom count, residue count, ligand count, and average ligand size. Each size factor group contains several interpretations of the complex property outlined in its name (e.g., the *molecular weight* factor group contains factors such as *total structure weight* and *ligand weight in structure*). Factors that quantify the size of whole preferred assemblies from several points of view have been included as well in a separate group. This group also includes the ligand flexibility factor (i.e., *flexibility ratio of ligands in preferred structure assembly*).

Biomacromolecule quality factors are grouped by the quality metric they have been derived from. All of them have been computed from the data of Worldwide Protein Data Bank (wwPDB) Validation Reports. Therefore, they encompass quality factors that are based on the agreement of atomic model and experimentally obtained data, e.g., R_{free} (Brunger, 1992), Real Space R-value (RSR) (Jones *et al.*, 1991) as well as its Z-score (RSRZ) (Kleywegt *et al.*, 2004), and residue real-space correlation coefficient (RSCC) (Yang *et al.*, 2016). Other quality factors are based on validation metrics that assess biopolymer geometry on the level of singular residues and their atoms, namely clashscore, percentages of Ramachandran outliers and sidechain outliers, and the root-mean-square value of the Z-scores (RMSZ) for bond angles and lengths (Chen *et al.*, 2010). The clashscore quality metric, which the factor of the same name is based on, quantifies the number of too close contacts between atoms per thousand atoms of a structure (Chen *et al.*, 2010).

Quality metrics that give one value per complex (e.g., RSRZ outlier percentage in residues) have been taken as factors as they were. Metrics that provide a separate value for each residue (e.g., RSR), however, have had their values arithmetically averaged for each PDB entry. Factors computed in this way have distinct names, e.g., *average structure RSR*. Additionally, the values of selected quality metrics (e.g., clashscore) are provided in wwPDB Validation Reports as percentile ranks. These ranks

show the quality of a PDB entry relative to the rest of entries in the PDB database. Each value signifies the percentage of complexes with lower or equal quality based on the metric. The percentile ranks of considered quality metrics have been included in the analysis as separate factors without any modifications. Factors created in this way have been named appropriately (e.g., *clashscore structure quality factor – percentile version*). Lastly, it is worth mentioning that two quality factors consider biomacromolecules on a per-chain basis (i.e., for the sake of their computation, an arithmetic average is produced for each chain). They represent each entry by the highest average RMSZ of bond angles (the factor *highest RMSZ of bond angles in residues of a chain in structure*) or bond lengths (the factor *highest RMSZ of bond lengths in residues of a chain in structure*) of all of its chains.

Ligand quality factors have been computed, as was mentioned earlier, from two sources. Those that represent the agreement between atomic models of ligands and experimentally measured data were computed from values of validation metrics listed in wwPDB Validation Reports, and are grouped by the quality metric they have been derived from, namely RSR and RSCC. Additionally, factors that quantify ligand model quality were considered as well. They were derived from the RMSZ for bond angles and lengths. All of the quality metrics mentioned in this paragraph provide one value per ligand of a complex. Values that have been assigned to each ligand in wwPDB Validation Reports have been averaged for each PDB entry and each metric, thus computing appropriately named quality factors, e.g., *average ligand RSCC*. In addition to the average factors, the RSCC metric is represented by an additional suitably named factor that shows the ratio of the number of ligand quality outliers to the total count of ligands in a complex, the *ratio of RSCC outliers among ligands* factor. Outliers are defined in this context as ligands whose RSCC metric value is below 0.8.The value of this threshold was taken from the official user guide to wwPDB Validation Reports (available at http://www.wwpdb.org/validation/2016/XrayValidationReportHelp).

Additional factors that represent ligand model quality in complexes are based on validation results that have been obtained from the ValidatorDB database. These can be divided into topological quality factors (which quantify atoms that are missing from ligands), chiral quality factors (which quantify the amount of ligand chiral carbon atoms with incorrect spatial configuration), and combined quality factors (which contain factors that combine both types of model issues). To further explore the domain of ligand model quality in the analysis, factors that are based on data from the ValidatorDB database have been enumerated via three approaches:

- Basic ligand quality factors: These factors are valued as the ratio of ligands with a problem to the total number ligands in a PDB entry (e.g., the factor *ratio of ligands with topological problems*).
- Relative ligand quality factors: These factors are valued as the ratio of the number of problematic features to the total number of features of all ligands in a PDB entry (e.g., the factor *relative ratio of atoms involved in carbon chirality problems* is valued as the ratio of the number of problematic chiral centers to the total number of chiral centers in all ligands in a PDB entry).
- Binary ligand quality factors: These factors are valued 1 for every PDB entry that fulfills the factor's criteria and 0 otherwise (e.g., factor *ratio of ligands without problems in topology and carbon chirality – binary version* is valued 1 for every PDB entry that has all ligands free of topology or carbon chirality errors; otherwise, it is valued 0).

Description of the statistical workflow

This section contains detailed description of the statistical processing workflow that was used for the analysis of trends between metadata and structure factors.

A note on dataset versions and manual statistical analysis workflow

It is important to note that information in this section refers to the "yearly 2017" dataset, which is the last dataset that was analyzed manually by a statistician. The most recent dataset differs from the "yearly 2017" dataset by including new and modified PDB entries, while excluding the obsolete ones. This automatically updated dataset is not put through the statistical workflow at this time, and therefore does not have its Spearman correlation coefficients updated. The next statistically analyzed dataset, the "yearly 2018", is expected to be made available by the end of May 2019.

Introduction and challenges

Statistical analysis of the dataset has been designed primarily to enable well-arranged visualization and characterization of the input data. Its other goal was to offer universal comparison of relationships between as many factor pairs as feasible, so as to offer comprehensive view on relationships in this analysis.

Input data contained information about 136,594 entries of the Protein Data Bank (PDB) database. This information was represented by values of 88 factors. Relationships between 1,852 pairs of factors have been assessed, since not all of the 7,656 possible ordered factor pairs were interesting for this project. Characteristic unwanted attribute of the dataset was high number of *NaN* values present in some factors (as shown in Table S2 at the end of this section). Another unwanted attribute was positive skewness (or negative skewness) of values of most factors and the occurrence of outliers situated to the right (or left) of most values. Additionally, nontrivial amount of values (up to 40 %) of some factors were equal to each other. Finally, a specific feature of the dataset was the presence of zero values.

Therefore, the statistical processing workflow has been designed in a way that preserves universal comprehensive view of relationships between factors, so that we have worked with all pairs of factors in the same way. The workflow also does not transform values, and accounts for skewness, unwanted amount of *NaN* values, and zero values.

Analysis of relationship between a factor pair

First, let us name two exemplar factors as X factor and Y factor. Further on in the text, we shall mention them independently, as well as a pair. Their notation comes from the axis on which they are visualized in plots in ValTrendsDB. It is also worth noting that some factors fulfill the role of an X factor in some plots while having the role of a Y factor in other plots.

The process of analyzing each factor pair can be divided into several steps:

1. PDB entries which have NaN value for the X factor were excluded from the analysis of this pair at this point. All PDB entries in the dataset were then sorted in ascending order by values of the X factor.
2. Then, the entries were classified into nonequidistant intervals (i.e., intervals with various width). This type of intervals has been chosen specifically because of the skewness of values. Values of most factors have been split into 100 intervals. A smaller count of intervals was

chosen for factors which values could not have been divided into 100 intervals (e.g., for factor *year of release*). Endpoints of intervals have been determined with the intention of achieving uniform split of values among intervals. This intention was in some cases challenged by too many occurrences of single value in several factors. Another criterion for value splitting was that for any combination of X and Y factor, each interval should contain at least 100 values. This criterion was challenged by varying count of *NaN* values among factors suited for the role of Y factor. Despite these challenges, we have determined a set of universal endpoints for intervals of each factor that can fulfill the role of X factor, so that, on average, more than 80 % of intervals contain at least 100 values regardless of the Y factor and its count of *NaN* values. Therefore, endpoints of intervals for X factors are universal: They do not depend on the concrete Y factor that is paired to an X factor.

3. PDB entries which have *NaN* value for the Y factor were excluded from the analysis of this pair at this point.
4. Each interval was checked if it contains at least 100 values after PDB entry culling in the previous step. Intervals that were not populated adequately were joined with their adjacent intervals either to the left, or to the right (depending on which of them had fewer values). An exception was the last interval to the right, which was right-unbounded. If this interval contained less than 100 values, it was not joined with the adjacent interval to the left. The reason is that if the last interval on the right was right-unbounded, it contained outliers in most cases, which were the cause of excessive length and variance of the interval. Therefore, such interval would be discarded from the analysis in later steps anyways (but they would not be discarded from visualization plots on the web page of ValTrendsDB). Had the second to last interval been mindlessly joined with the last one, its values would be lost from the analysis after culling of the last outlier-heavy interval.
5. Arithmetic mean was then computed for each interval, both of values of factor X and Y separately. That is to say, if values of factor X were sorted into 100 intervals, 100 arithmetic means were computed for factor X and 100 were computed for factor Y. Arithmetic mean has been chosen specifically to preserve interpretability of values in plots of factor pairs.
6. Next, we culled intervals which value of the arithmetic mean was a severe outlier to values of the arithmetic means of the rest of intervals. Culled intervals were in all cases left-unbounded, or right-unbounded (in the context of this analysis, they were either the first interval and the following ones, or the last interval and the preceding ones). The amount of PDB entries sorted into culled intervals ranged from 0 to 12 %.
7. Arithmetic means that belonged to remaining intervals were used to assess the relationship (i.e., association) between factors X and Y. The Spearman's rank correlation coefficient R_s (or Spearman's coefficient in short) has been used as quantifier for intensity of the association. Values of Spearman's coefficient range from -1 to 1. Positive coefficient value indicates direct rank association between factors X and Y, while negative coefficient value indicates indirect rank association. The concrete values of Spearman's coefficient can also be used to determine strength of the examined relationship. To measure the degree of association using Spearman's coefficient, a criterion table that distinguishes eight degrees of association is commonly used. However, such distinction is too delicate for our dataset. Therefore, we have instead used a simplified table (Table S1). An exact definition of Spearman's coefficient can be found further in this section.

Finally, let us remark that values of arithmetic means for factors X and Y in each interval depend only on which PDB entries have been sorted into an interval via their value of X factor. If the entries were sorted into intervals via their value of Y factor, interval endpoints and values of arithmetic mean for both factors for each interval would be different, and thus the value of Spearman's coefficient would be different.

Table S1. Criterion table used to interpret values of Spearman's rank correlation coefficient R_s.

R_s	Interpretation of the value	R_s	Interpretation of the value
[0; 0.3)	Weak or nonexistent degree of direct rank association	(-0.3; 0]	Weak or nonexistent degree of indirect rank association
[0.3; 0.7)	Moderate degree of direct rank association	(-0.7; -0.3]	Moderate degree of indirect rank association
[0.7; 1]	Strong degree of direct rank association	[-1; -0.7]	Strong degree of indirect rank association

Relationship visualization design in ValTrendsDB

One of the goals of ValTrendsDB is to intuitively visualize and characterize relationships between factors that represent either quality, or various properties of biomacromolecular complexes. The visualization has been designed so as to provide global, comprehensive, and universal view on relationships between significant pairs of factors. The process of computing the visualization data comes from the process of the analysis that is described in the previous section, but diverges from it in some of the steps.

The process of visualizing the relationship between two factors (let us name them X and Y – for details, see the start of the previous subsection) was carried out in several steps:

1. PDB entries which have *NaN* value for the X factor were excluded from the analysis of this pair at this point. All PDB entries in the dataset were then sorted in ascending order by values of the X factor and sorted into nonequidistant intervals by values of the same factor. The concrete procedure for doing so is described in the first two steps of the analysis.
2. PDB entries which have *NaN* value for the Y factor were excluded from the analysis of this pair at this point.
3. Each interval was checked if it still contains at least 100 values after PDB entry culling in the previous step. Intervals that were not populated adequately were joined with adjacent intervals. Unlike in the analysis, the last interval to the right was checked as well.
4. Arithmetic mean of Y factor values was then computed for each interval. In addition, minimum, lower quartile, median, upper quartile, and maximum have been computed for each interval from values of the Y factor.
5. Data distribution in the intervals has been represented using box plot – each interval has its own box. All intervals have been included in the visualization – even those that contain outliers. This decision was enacted in line with our effort to create a complete visualization of all valid data of each factor pair.

Boxes[1] of the box plots, used on the web of ValTrendsDB, are top-delimited and bottom-delimited by short black horizontal line segments. These line segments represent values of lower quartile (horizontal line segment located on the bottom end of the vertical line) and upper quartile (horizontal line segment located on the top end of the vertical line) of the Y factor. Its median value is also visualized by a short horizontal line segment which has its place between upper and lower quartile values[2]. Finally, values of arithmetic mean of the Y factor are shown as red dots. To preserve clarity of the plots, outlying and extreme (minimum, maximum) data points are not shown.

Values of all statistical quantities that describe Y factor data have been assigned to the primary Y axis that is located on the left edge of the plot. The X axis which visualizes intervals of the X factor is located on the bottom edge of the plot. In the case of our visualization design, it is more informative for users of ValTrendsDB to visualize interval endpoints of the X factor rather than values of the arithmetic mean. Do note however that even though all intervals are shown as equidistant to make the plot as clear as possible, their true length varies.

Absolute frequency of PDB entries that have been sorted into intervals is visualized for each interval on the background of the box plot using a gray bar plot. Its assigned axis is the secondary Y axis on the right edge of the plot. The scale of the bar plot is logarithmic.

An example of a plot that visualizes relationship between two factors in ValTrendsDB is shown in Figure S1.

Definition of Spearman's rank correlation coefficient

Suppose that x_1, x_2, …, x_n are realizations of factor X and y_1, y_2, …, y_n are realizations of factor Y (therefore, we have n pairs x_i, y_i, $i = 1, \dots, n$). We sort values x_i from lowest to highest and determine their rank p_i. Then, we sort values y_i from lowest to highest and determine their rank q_i.

The Spearman's rank correlation coefficient can be computed using formula

$$R_S = 1 - \frac{6\sum_{i=1}^{n}(p_i - q_i)^2}{n(n^2-1)}.$$

[1] In plots that visualize many intervals, a box may be drawn as a single vertical line instead. In this case, visualization of quantiles using short horizontal line segments is unchanged.

[2] In some plots, not all three line segments of boxes in the box plot are visible. This phenomenon signifies the fact that two of the quantiles can have same values. Additionally, all three quantiles can have the same value. In such case, only one horizontal line segment is visible in place of a box.

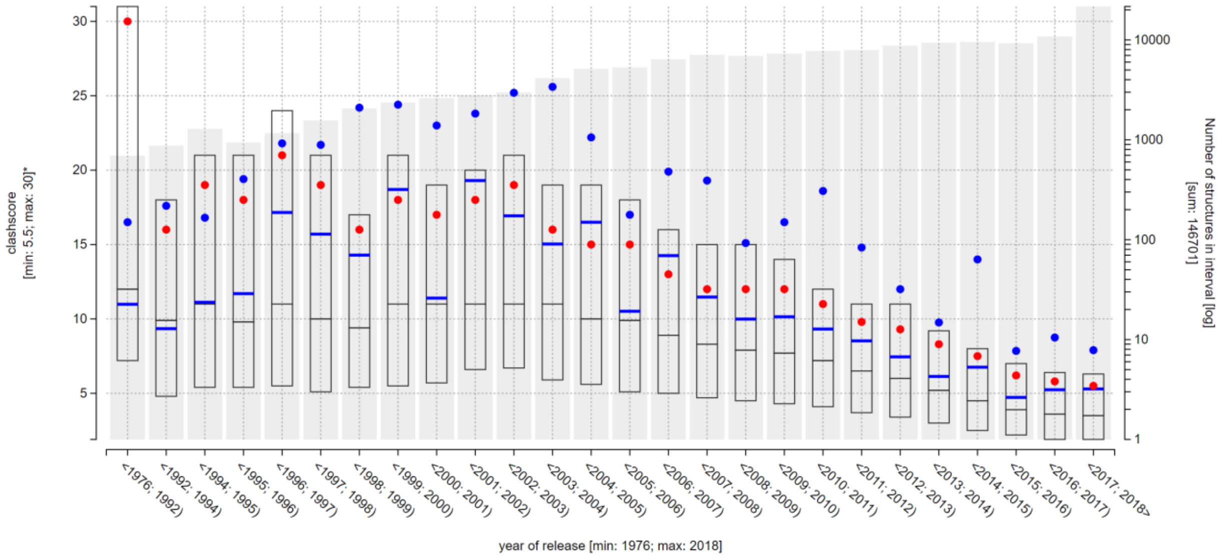

*clashscore structure quality factor

Figure S1. Example of a plot from ValTrendsDB showing relationship between clashscore (Gore *et al.*, 2017) quality factor and year of release of PDB entries. Clashscore is represented in each interval by an arithmetic average (red dot), median, lower quartile, and upper quartile (box plot). The number of entries in each interval is visualized by a gray bar plot. Average (blue dot) and median (blue line) clashscore of PDB entries, published in journals Nature and Science, is shown for comparison.

Table S2

Table S2. Notable value counts of each factor considered in the analysis.

factor name	total values	*NaN* values	"0" values	"1" values	"100" values
atom count (structure and ligand atoms)	136,594	0	0	0	1
atom count (structure and ligand atoms) on logarithmic scale	136,594	0	0	0	0
atom count of ligands in structure	136,594	0	12,911	263	292
atom count of ligands in structure, considering only ligands containing metal atoms	136,594	0	85,175	12,230	18
atom count of ligands in structure, considering only ligands validated by MotiveValidator	136,594	191	60,555	0	278
atom count of ligands in structure, considering only ligands validated by MotiveValidator that contain metal atoms	136,594	191	129,617	0	3
atom count of ligands in structure, considering only ligands validated by MotiveValidator, without ligands containing metal atoms	136,594	191	63,805	0	271
atom count of ligands in structure, disregarding water ligands	136,594	0	27,837	2,307	404
atom count of ligands in structure, disregarding water ligands and ligands containing metal atoms	136,594	0	38,059	753	374
atom count of ligands in structure, without ligands containing metal atoms	136,594	0	15,368	133	280
average ligand LLDF	136,594	57,146	74	58	0
average ligand LLDF of big ligands	136,594	80,111	104	59	0
average ligand LLDF of small ligands	136,594	88,609	36	38	0
average ligand RSCC	136,594	52,316	0	3	0
average ligand RSCC of big ligands	136,594	74,927	0	0	0

factor name	total values	*NaN* values	"0" values	"1" values	"100" values
average ligand RSCC of small ligands	136,594	84,246	0	3	0
average ligand size in structure	136,594	12,911	0	24,072	4
average ligand size in structure without ligands containing metal atoms	136,594	15,368	0	24,068	5
average ligand size in structure without ligands containing metal atoms, considering only ligands validated by MotiveValidator	136,594	63,996	0	0	0
average ligand size in structure without ligands containing metal atoms, disregarding water ligands	136,594	38,059	0	2,382	9
average ligand size in structure, considering only ligands validated by MotiveValidator	136,594	60,746	0	0	0
average ligand size in structure, considering only ligands validated by MotiveValidator and ligands containing metal atoms	136,594	129,808	0	0	0
average ligand size in structure, considering only ligands with metal atoms	136,594	85,175	0	42,496	0
average ligand size in structure, disregarding water ligands	136,594	27,837	0	10,047	8
average residue RSCC	136,594	24,827	0	0	0
average RMSZ of bond angles in ligands	136,594	42,884	2576	160	0
average RMSZ of bond angles in residues	136,594	781	0	656	0
average RMSZ of bond lengths in ligands	136,594	42,884	1047	174	0
average RMSZ of bond lengths in residues	136,594	781	0	572	0
average RSR of ligands in structure	136,594	52,316	0	0	0
average RSR of residues in structure	136,594	24,827	0	0	0
biopolymer weight in structure [kDa]	136,594	0	25	0	0
clashscore structure quality factor	136,594	457	1,991	82	1
clashscore structure quality factor - percentile version	136,594	393	109	195	2,131
flexibility ratio of ligands in preferred structure assembly	136,594	34,055	6,871	13,243	0
highest RMSZ of bond angles in residues of a chain in structure	136,594	781	0	759	0
highest RMSZ of bond lengths in residues of a chain in structure	136,594	781	0	638	0
chiral carbon count in ligands	136,594	191	82,098	4,790	75
ligand and water weight in structure [kDa]	136,594	0	14,752	0	0
ligand count in structure	136,594	0	12,911	2,758	329
ligand count in structure without ligands containing metal atoms	136,594	0	15,368	2,355	340
ligand count in structure without ligands containing metal atoms, considering only ligands validated by MotiveValidator	136,594	191	63,805	23,681	0
ligand count in structure without ligands containing metal atoms, disregarding water ligands	136,594	0	38,059	17,621	29
ligand count in structure, considering only ligands validated by MotiveValidator and ligands containing metal atoms	136,594	191	129,617	2,638	0
ligand count in structure, disregarding water ligands	136,594	0	27,837	13,900	45
ligand count, considering only ligands validated by MotiveValidator	136,594	191	60,555	24,262	0
ligand count, considering only ligands with metal atoms, in structure	136,594	0	85,175	14,861	12
ligand weight in structure [kDa]	136,594	0	34,171	0	0
number of unique biopolymer units in preferred structure assembly	136,594	0	26	106,994	0
number of unique ligand molecules in preferred structure assembly	136,594	0	34,055	35,875	0
Ramachandran outliers structure quality factor - percentile version	136,594	3,956	107	171	57,881
Ramachandran outliers structure quality factor [%]	136,594	3,956	57,502	177	1
ratio of ligands with problems in carbon chirality	136,594	60,746	73,167	593	0
ratio of ligands with topological problems	136,594	60,746	67,967	2,136	0
ratio of ligands without problems in topology and carbon chirality	136,594	60,746	2,761	65,729	0
ratio of ligands without problems in topology and carbon chirality -	136,594	60,746	10,119	65,729	0

factor name	total values	*NaN* values	"0" values	"1" values	"100" values
binary version					
ratio of LLDF outliers among ligands	136,594	57,146	31,435	8,008	0
ratio of RSCC outliers among ligands	136,594	52,316	54,563	3,073	0
ratio of RSCC outliers among residues	136,594	24,827	513	23	0
ratio of single bonds in ligands	136,594	60,746	0	6,704	0
relative ratio of atoms involved in carbon chirality problems	136,594	60,746	72,609	259	0
relative ratio of atoms involved in carbon chirality problems - binary version	136,594	60,746	72,609	3,239	0
relative ratio of atoms involved in topological problems	136,594	60,746	68,849	0	0
relative ratio of atoms involved in topological problems - binary version	136,594	60,746	68,849	6,999	0
relative ratio of atoms involved in topological problems and carbon atoms in carbon chirality problems	136,594	60,746	67,946	217	0
relative ratio of atoms involved in topological problems and carbon atoms in carbon chirality problems - binary version	136,594	60,746	67,946	7,902	0
residue and ligand count in structure	136,594	0	0	0	127
residue and ligand count in structure, considering only ligands validated by MotiveValidator	136,594	0	0	3	685
residue and ligand count in structure, disregarding water ligands	136,594	0	0	0	432
residue count in structure	136,594	0	25	0	253
R_{free}	136,594	32,676	0	0	0
RSRZ outlier percentage in residues - percentile version	136,594	24,865	55	107	5,788
RSRZ outlier percentage in residues [%]	136,594	24,865	5,756	197	33
R_{value}	136,594	25,573	0	0	0
sidechain outliers structure quality factor - percentile version	136,594	4,148	111	208	7,600
sidechain outliers structure quality factor [%]	136,594	4,148	7,571	254	4
structure atom count (without ligand atoms)	136,594	0	68	0	2
structure resolution [Å]	136,594	13,404	0	202	0
total biopolymer unit count of preferred structure assembly	136,594	0	26	60,142	0
total biopolymer weight of preferred structure assembly [Da]	136,594	0	26	0	0
total ligand molecule count in preferred structure assembly	136,594	0	34,055	16,537	3
total ligand weight of preferred structure assembly [Da]	136,594	0	34,222	0	0
total structure weight [kDa]	136,594	0	0	0	0
total water molecule count of preferred structure assembly	136,594	0	23,405	49,334	0
total water weight of preferred structure assembly [Da]	136,594	0	23,405	0	0
total weight of preferred structure assembly [Da]	136,594	0	0	0	0
water weight in structure [Da]	136,594	0	23,378	0	0
year of release	136,594	0	0	0	0

Plots of discussed trends

Selected plots from ValTrendsDB are shown in this section. All of them are based on the "yearly 2017" version of the analysis. Color coding of the Spearman coefficients can be found in table S1 on page 6. Note that plots in this section are simplified (e.g., there is no underlying bar plot and secondary Y axis) to emphasize discussed trends.

Geometry quality of biomacromolecular structures in time

Selected plots S2 to S8 show strong relationships between the year of release of PDB structures and factors that represent geometric quality of PDB structures. These relationships clearly demonstrate that geometric quality of PDB structures is improving in time.

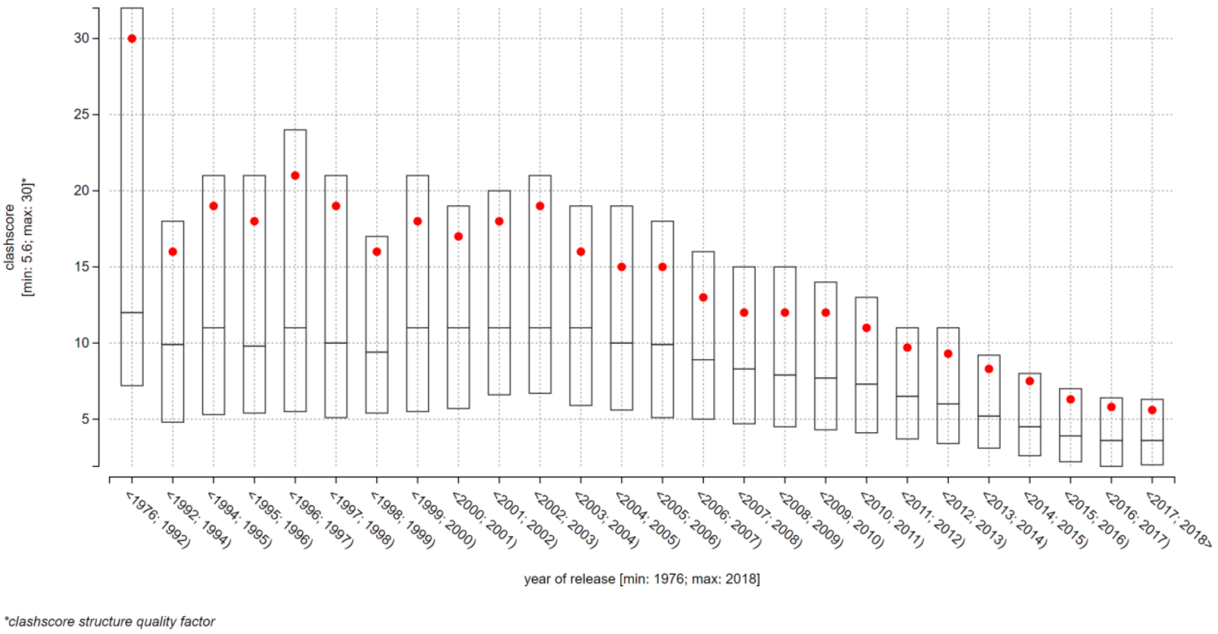

Figure S2. Relationship between factors *year of release* and *clashscore structure quality factor*. Lower values represent higher quality. Spearman's coefficient $\mathbf{R_s}$ **= -0.9635**.

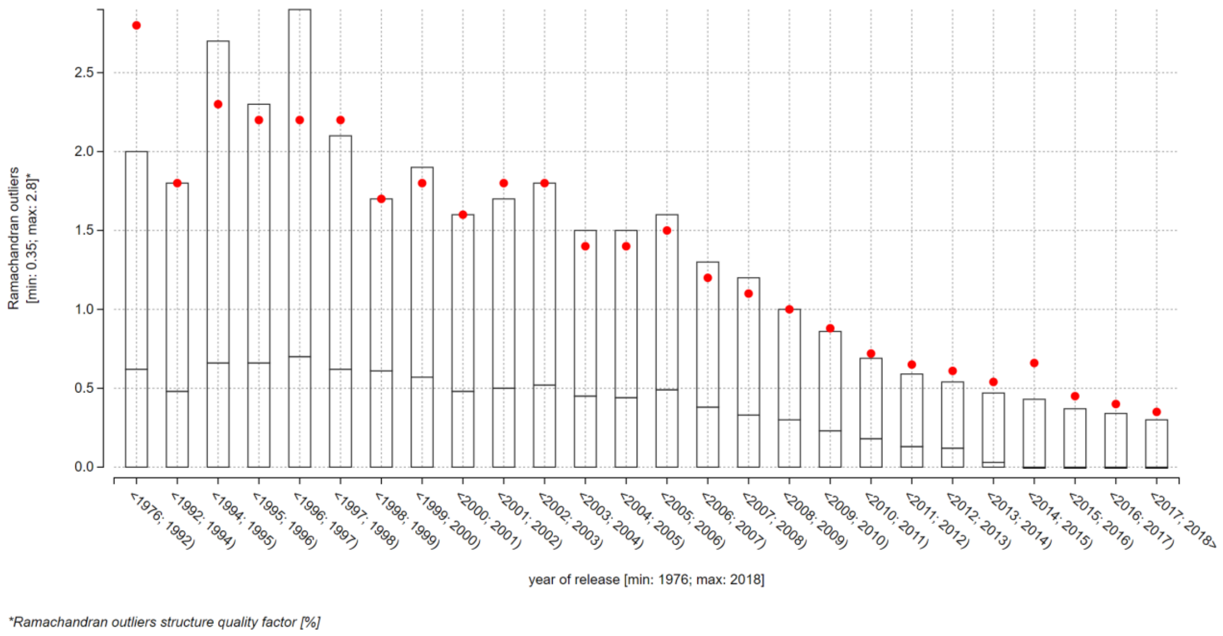

Figure S3. Relationship between factors *year of release* and *Ramachandran outliers structure quality factor*. Lower values represent higher quality. Spearman's coefficient $\mathbf{R_s}$ **=** **-0.9817**.

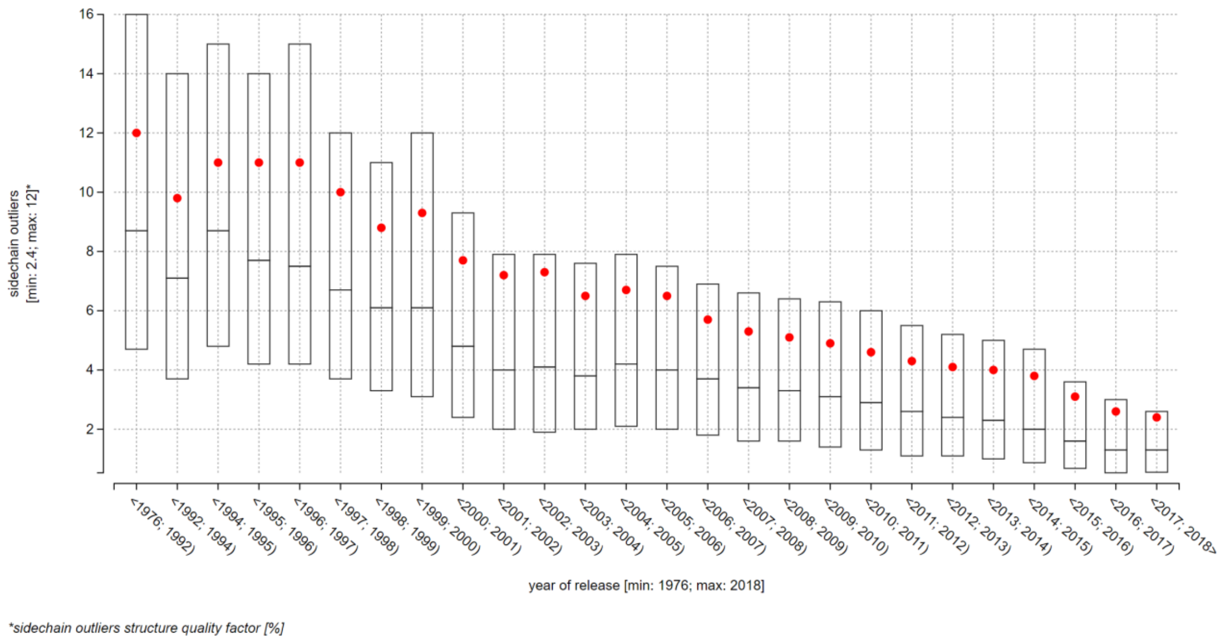

Figure S4. Relationship between factors *year of release* and *sidechain outliers structure quality factor*. Lower values represent higher quality. Spearman's coefficient $\mathbf{R_s}$ **=** **-0.9965**.

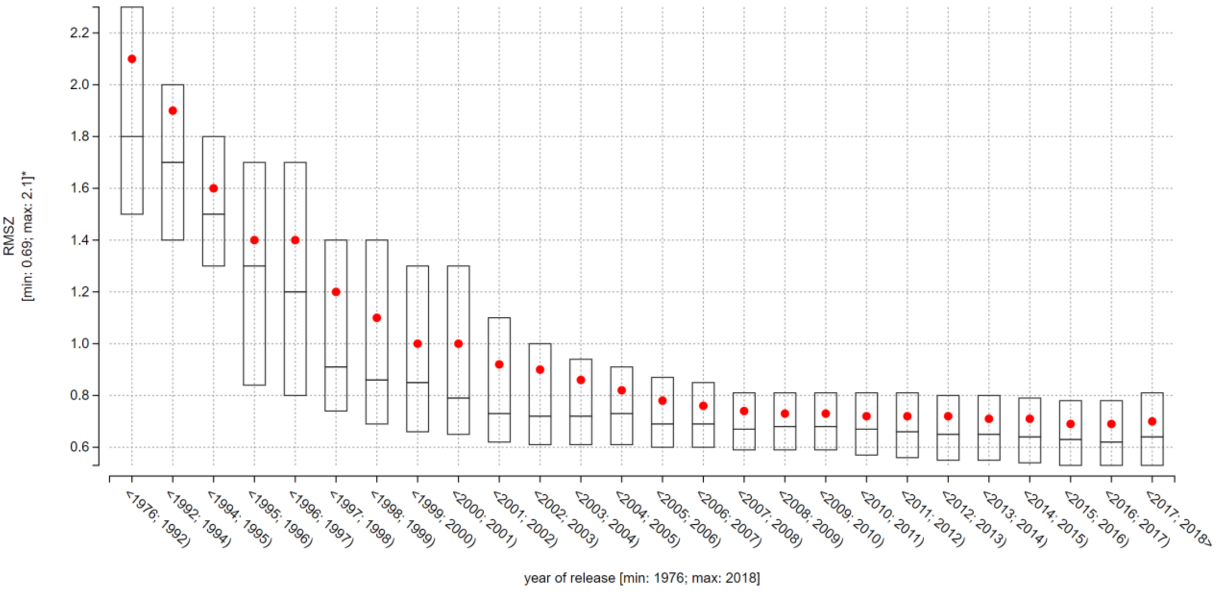

**average RMSZ of bond angles in residues*

Figure S5. Relationship between factors *year of release* and *average RMSZ of bond angles in residues*. Lower values represent higher quality. Spearman's coefficient $\mathbf{R_s}$ **= -0.9957**.

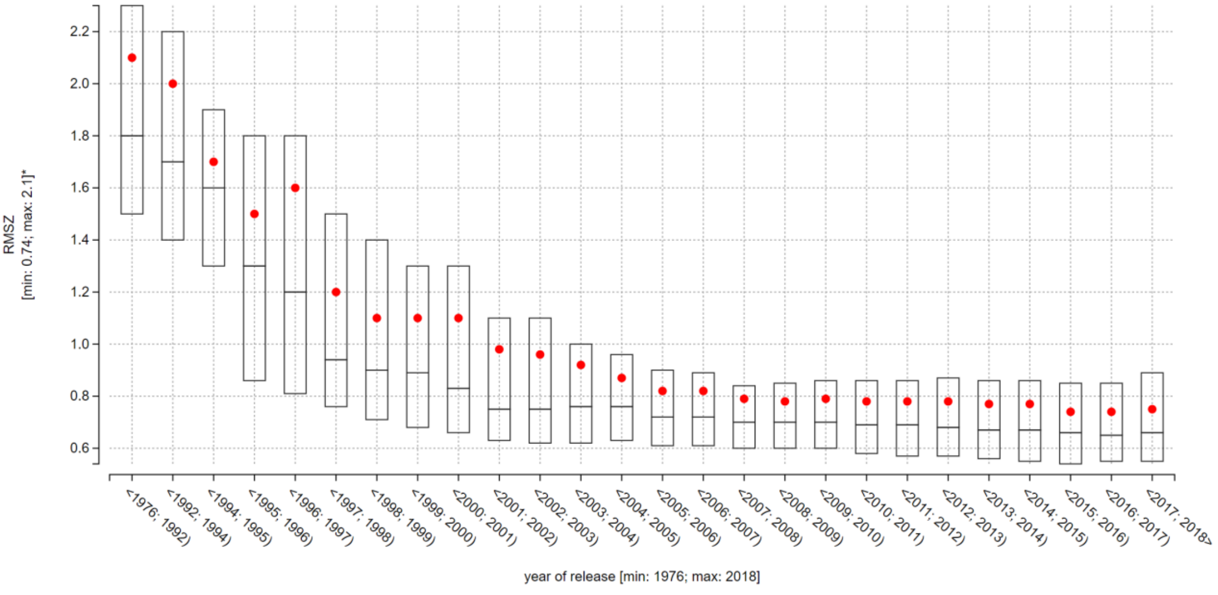

**highest RMSZ of bond angles in residues of a chain in structure*

Figure S6. Relationship between factors *year of release* and *highest RMSZ of bond angles in residues of a chain in structure*. Lower values represent higher quality. Spearman's coefficient $\mathbf{R_s}$ **= -0.9896**.

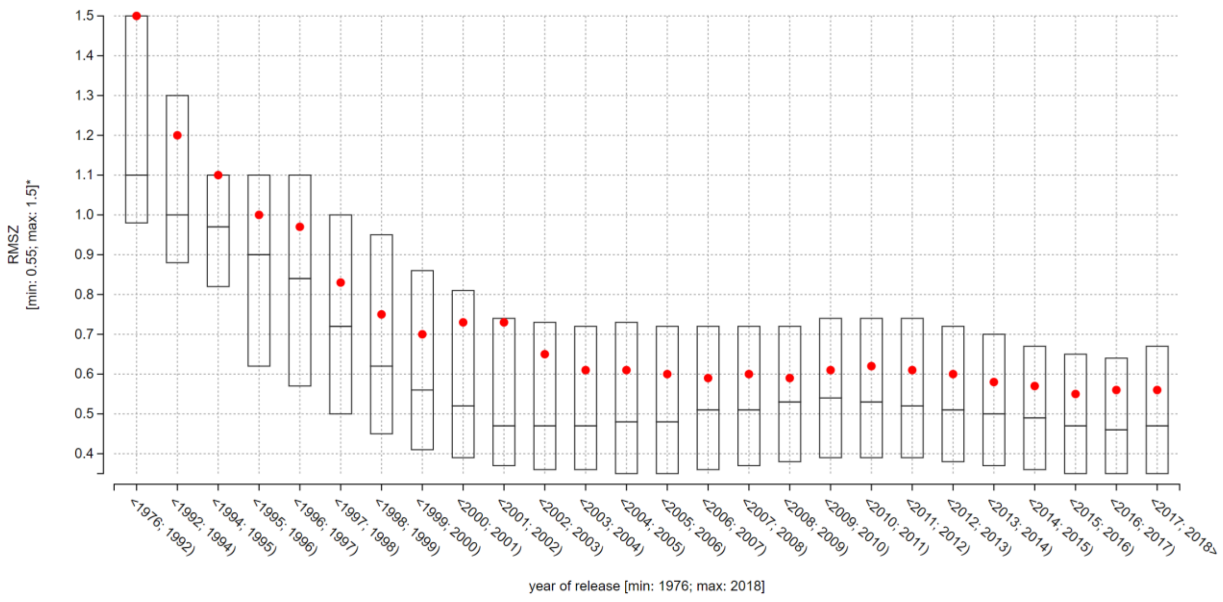

**average RMSZ of bond lengths in residues*

Figure S7. Relationship between factors *year of release* and *average RMSZ of bond lengths in residues*. Lower values represent higher quality. Spearman's coefficient $\mathbf{R_s}$ **= -0.9104**.

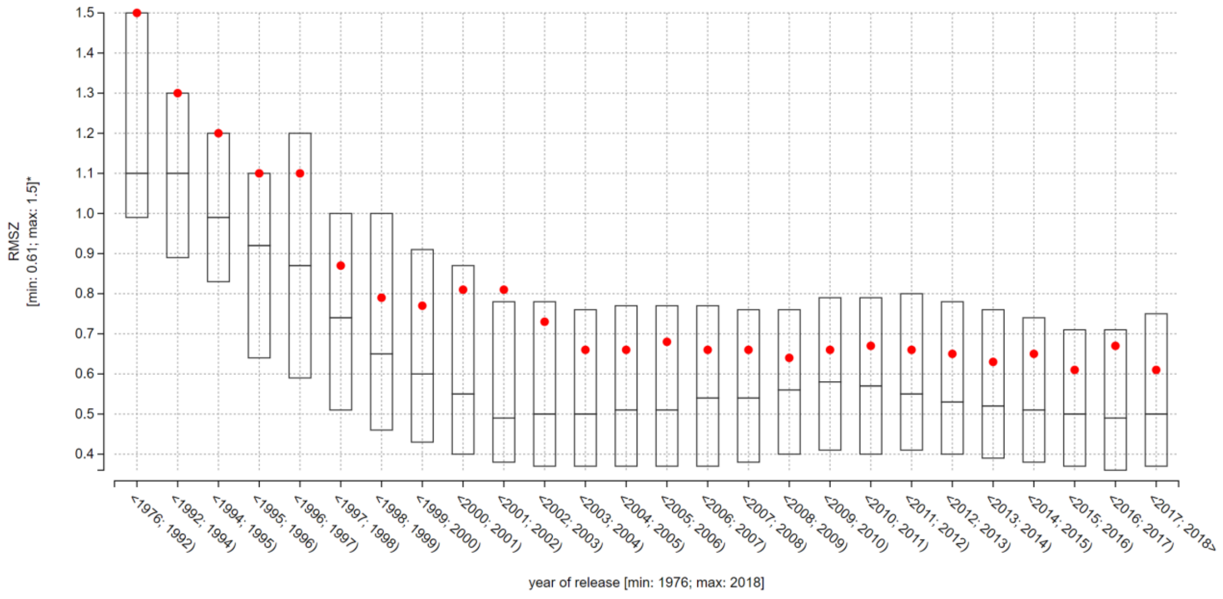

**highest RMSZ of bond lengths in residues of a chain in structure*

Figure S8. Relationship between factors *year of release* and *highest RMSZ of bond lengths in residues of a chain in structure*. Lower values represent higher quality. Spearman's coefficient $\mathbf{R_s}$ **= -0.8748**.

Quality of agreement between biomacromolecule structure models and their source electron density in time

Selected plots S9 to S11 show strong relationships between the year of release of PDB structures and factors that represent quality of agreement between PDB structure model and its underlying experimental data. On the contrary, plots S12 to S14 show no relationship between the above mentioned factors. Therefore, it is not clear whether fidelity of PDB structures improves in time, or not.

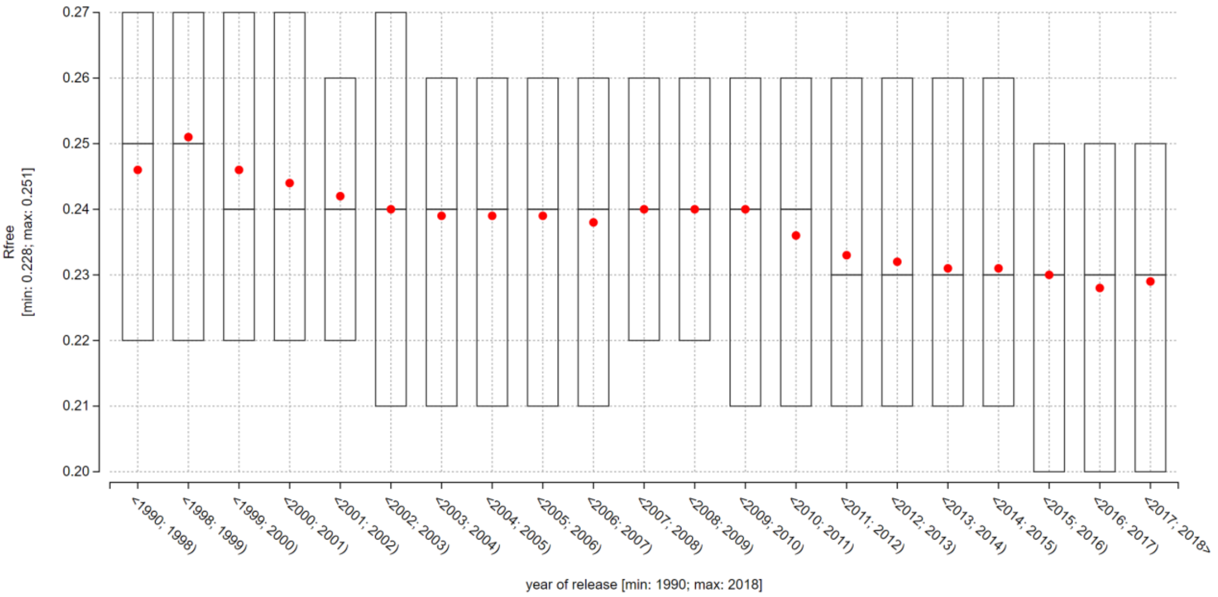

Figure S9. Relationship between factors *year of release* and R_{free}. Lower values represent higher quality. Spearman's coefficient $\mathbf{R_s}$ **=** **-0.9228**.

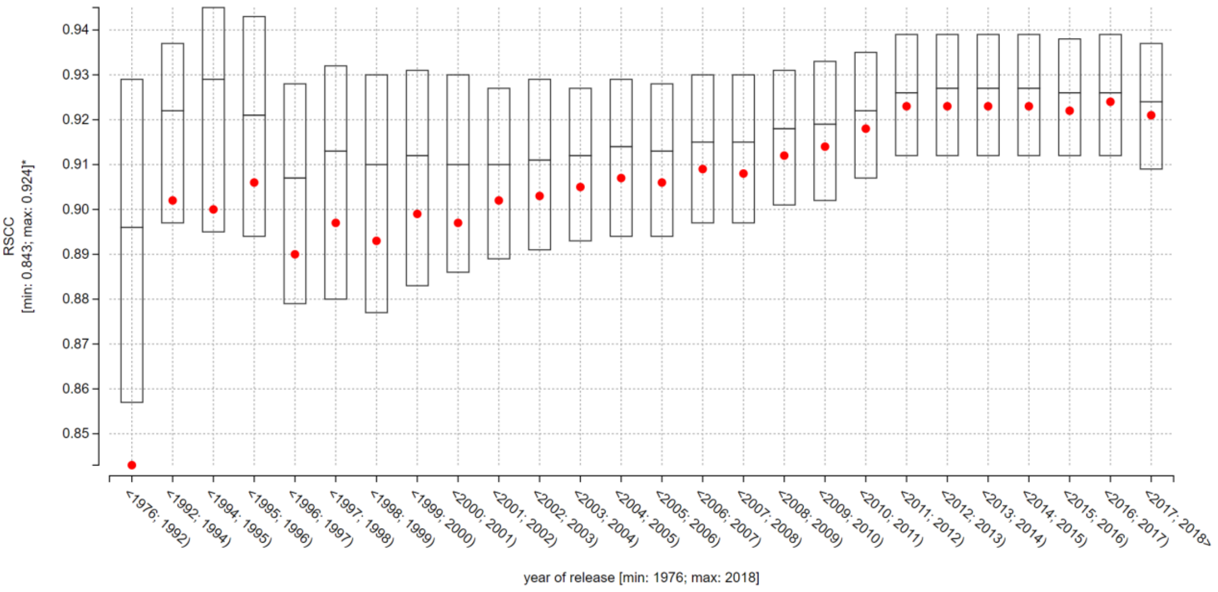

*average residue RSCC

Figure S10. Relationship between factors *year of release* and *average residue RSCC*. Higher values represent higher quality. Spearman's coefficient $\mathbf{R_s}$ **=** **0.9061**.

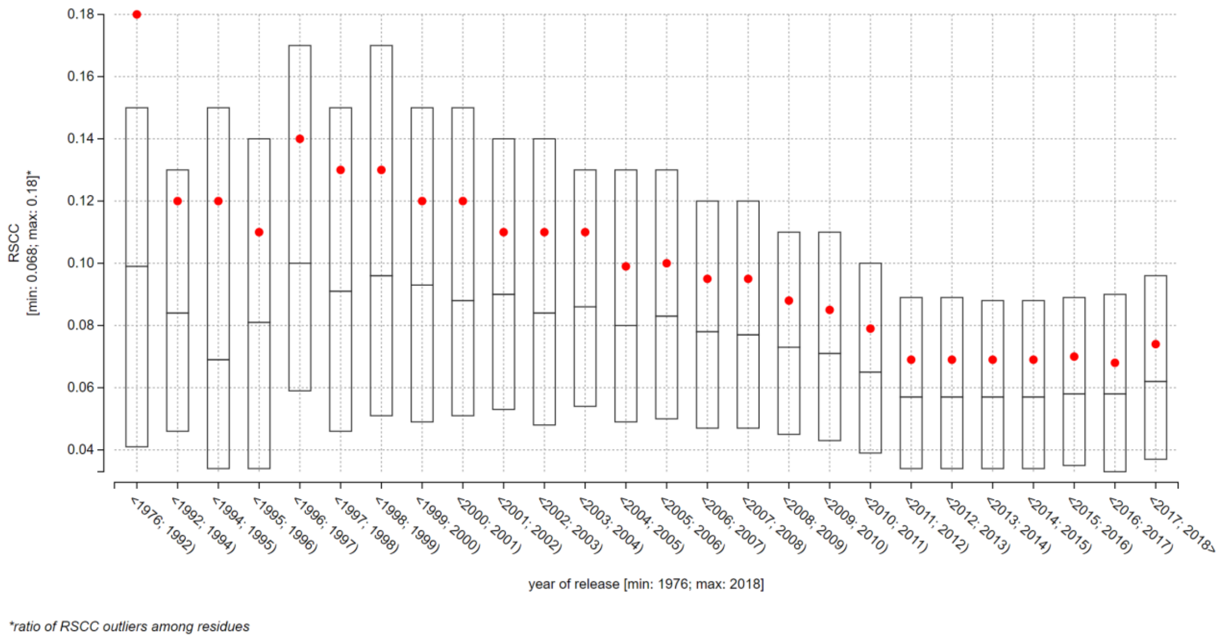

Figure S11. Relationship between factors *year of release* and *ratio of RSCC outliers among residues*. Lower values represent higher quality. Spearman's coefficient $\mathbf{R_s}$ **=** **-0.9409**.

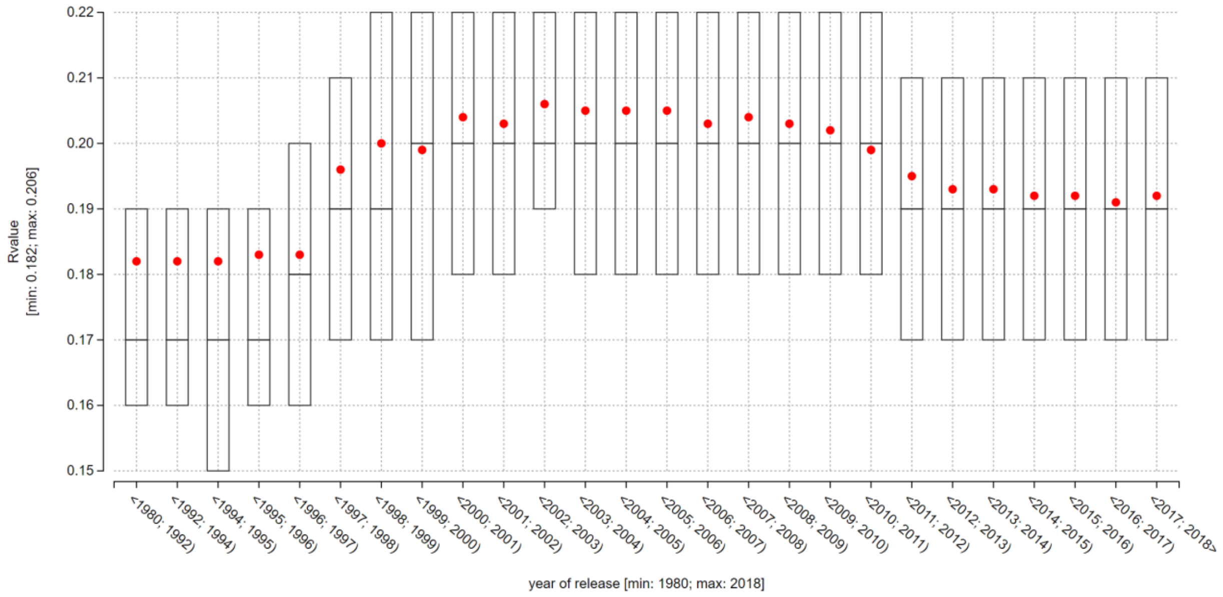

Figure S12. Relationship between factors *year of release* and R_{value}. Lower values represent higher quality. Spearman's coefficient $\mathbf{R_s}$ **=** **-0.1183**.

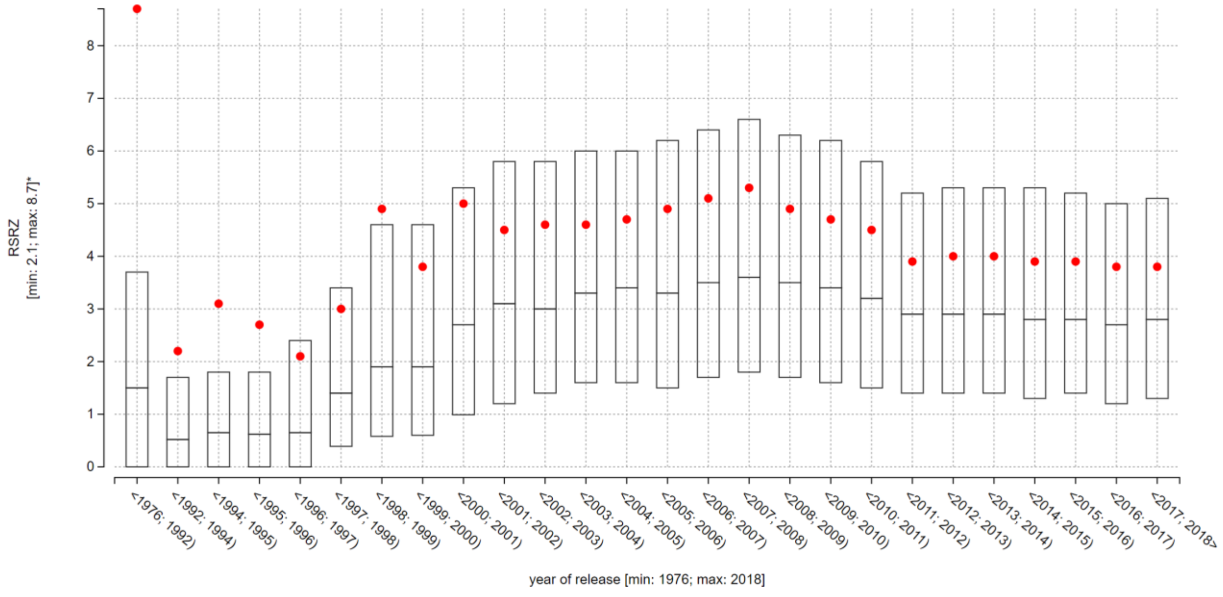

*RSRZ outlier percentage in residues [%]

Figure S13. Relationship between factors *year of release* and *RSRZ outlier percentage in residues*. Lower values represent higher quality. Spearman's coefficient $\mathbf{R_s}$ **= 0.1226**.

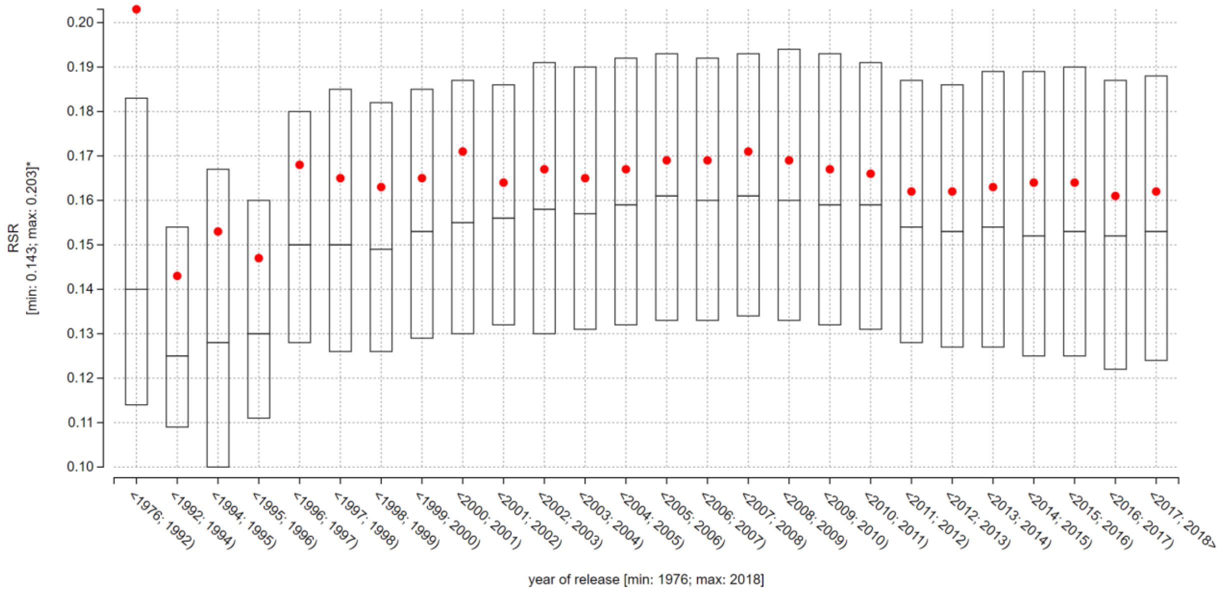

*average RSR of residues in structure

Figure S14. Relationship between factors *year of release* and *average RSR of residues in structure*. Lower values represent higher quality. Spearman's coefficient $\mathbf{R_s}$ **= -0.1365**.

Geometry quality of ligands in time

Selected plots S15 to S17, as well as plot S19, show moderate or strong relationships between the year of release of PDB structures and factors that represent geometric quality of ligands in PDB structures. Relationships in these plots show that geometric quality of ligands is improving in time. A relationship that does not support this trend is shown in plot S18.

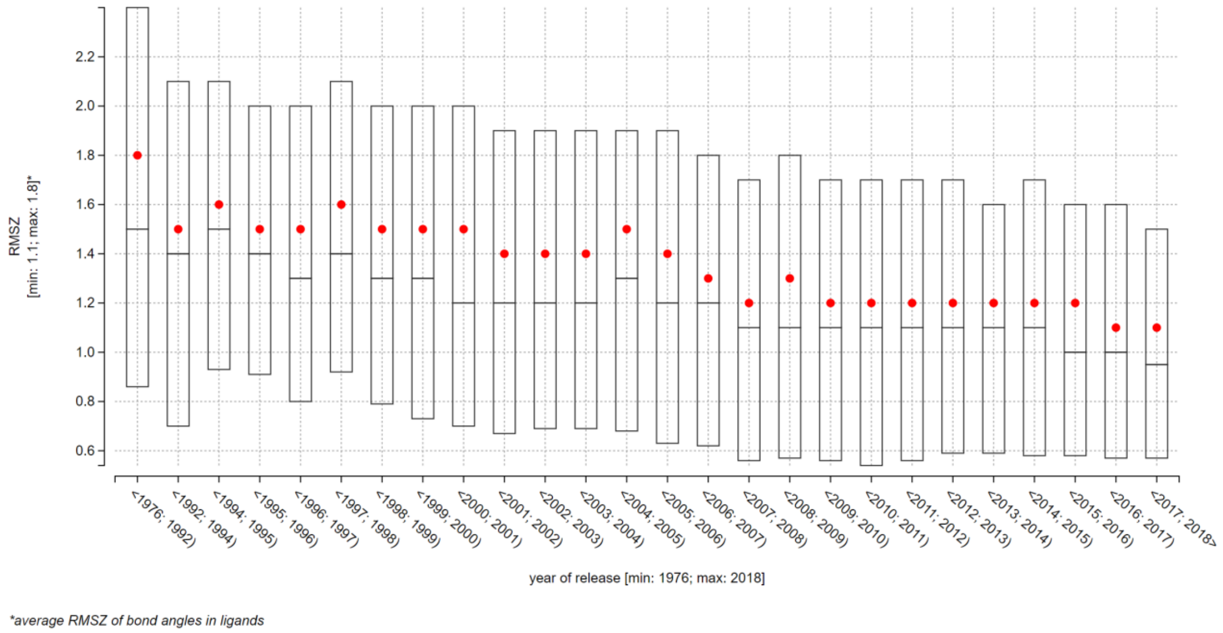

Figure S15. Relationship between factors *year of release* and *average RMSZ of bond angles in ligands*. Lower values represent higher quality. Spearman's coefficient $\mathbf{R_s}$ **= -0.9722**.

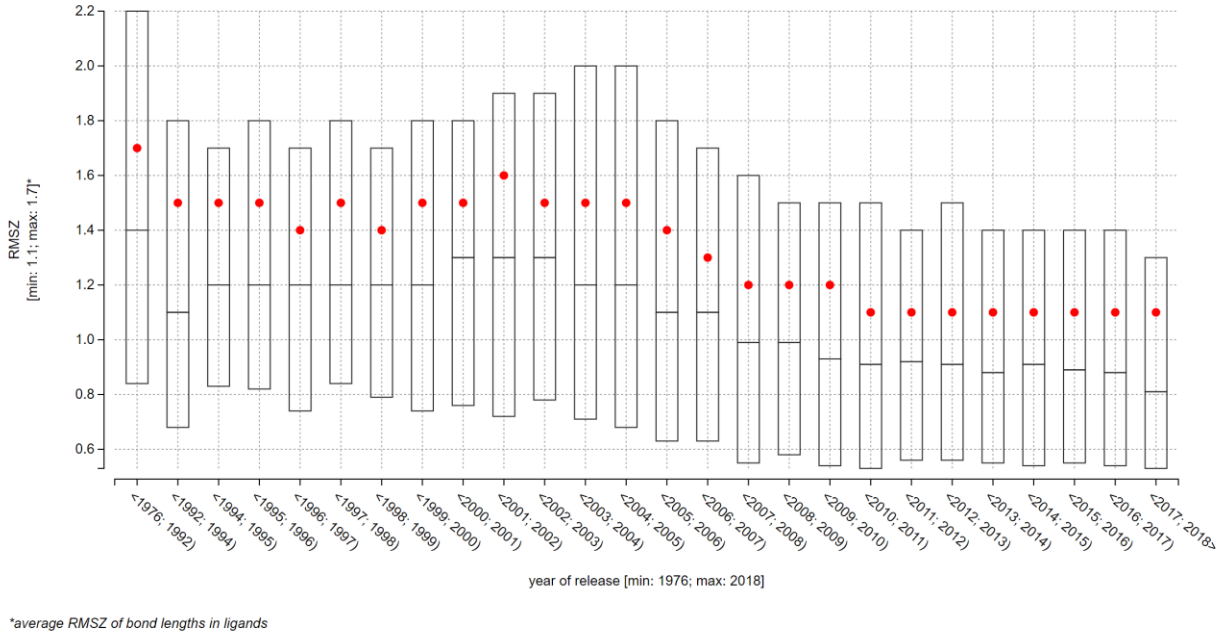

Figure S16. Relationship between factors *year of release* and *average RMSZ of bond lengths in ligands*. Lower values represent higher quality. Spearman's coefficient $\mathbf{R_s}$ **= -0.8304**.

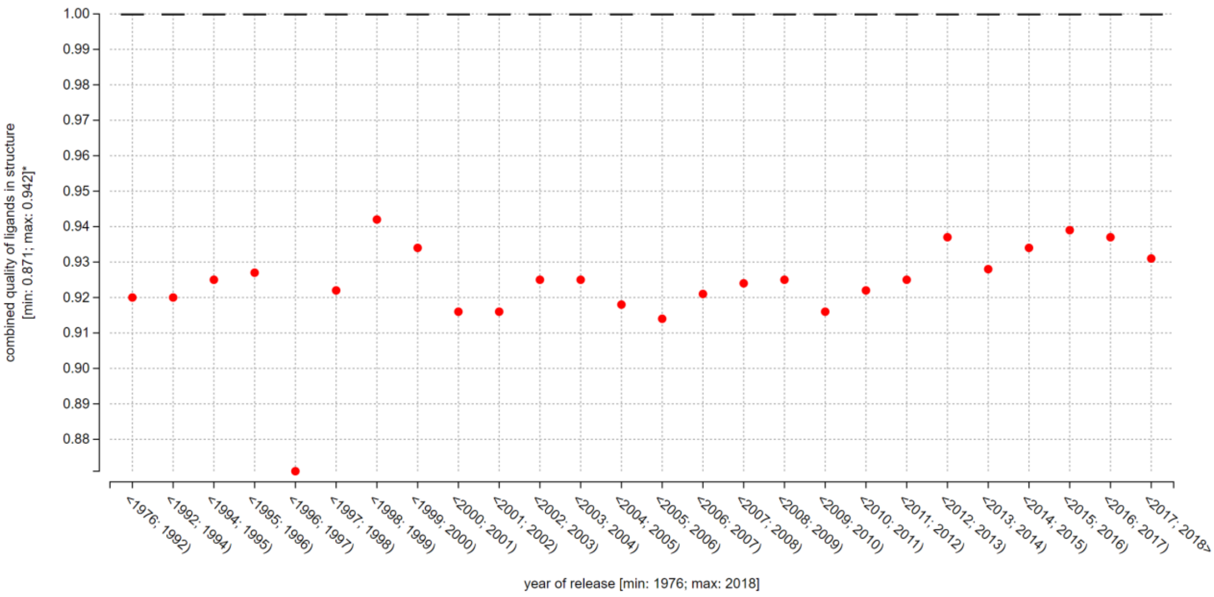

ratio of ligands without problems in topology and carbon chirality

Figure S17. Relationship between factors *year of release* and *ratio of ligands without problems in topology and carbon chirality*. Higher values represent higher quality. Note that all medians and all quartiles in the plot are equal to 1. Spearman's coefficient **R_s = 0.3696**.

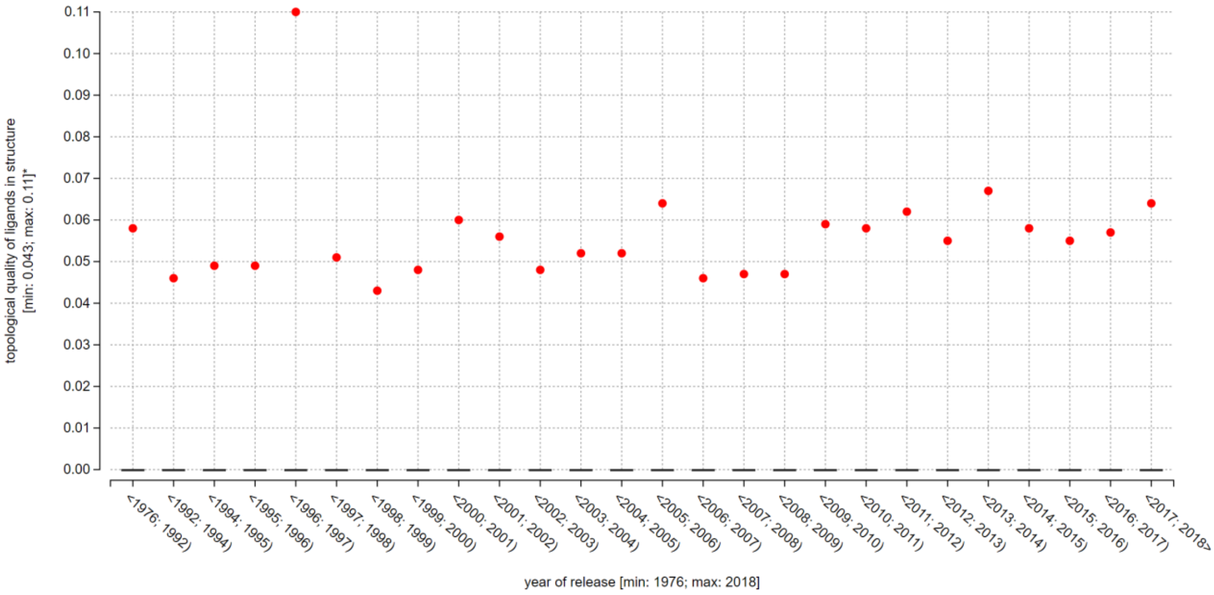

ratio of ligands with topological problems

Figure S18. Relationship between factors *year of release* and *ratio of ligands with topological problems*. Lower values represent higher quality. Note that all medians and all quartiles in the plot are equal to 0. Spearman's coefficient **R_s = 0.3704**.

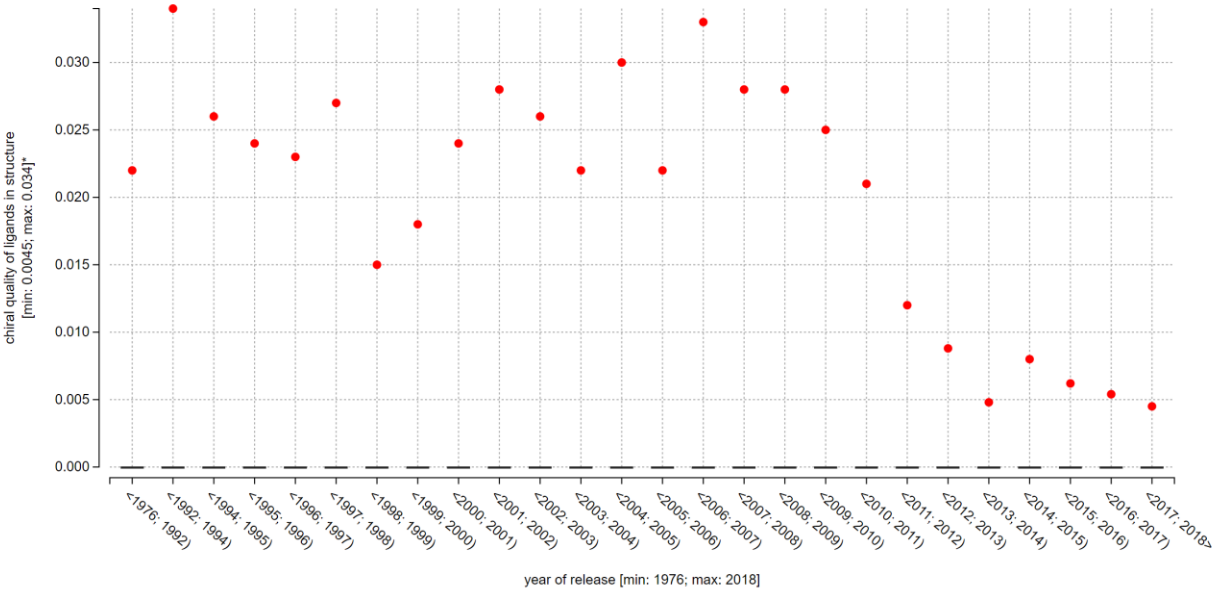

**ratio of ligands with problems in carbon chirality*

Figure S19. Relationship between factors *year of release* and *ratio of ligands with problems in carbon chirality*. Lower values represent higher quality. Note that all medians and all quartiles in the plot are equal to 0. Spearman's coefficient **R_s = -0.5696**.

Quality of agreement between ligand models and their source electron density in time

Selected plots S20 to S22 show relationships between the year of release of PDB structures and factors that represent quality of agreement between ligand model and its underlying experimental data. Plots S21 and S22 show weak or nonexistent relationships, while plot S20 shows moderate relationship that suggest decline in ligand model fidelity in time. Therefore, it is not clear whether ligand model fidelity improves in time, or not.

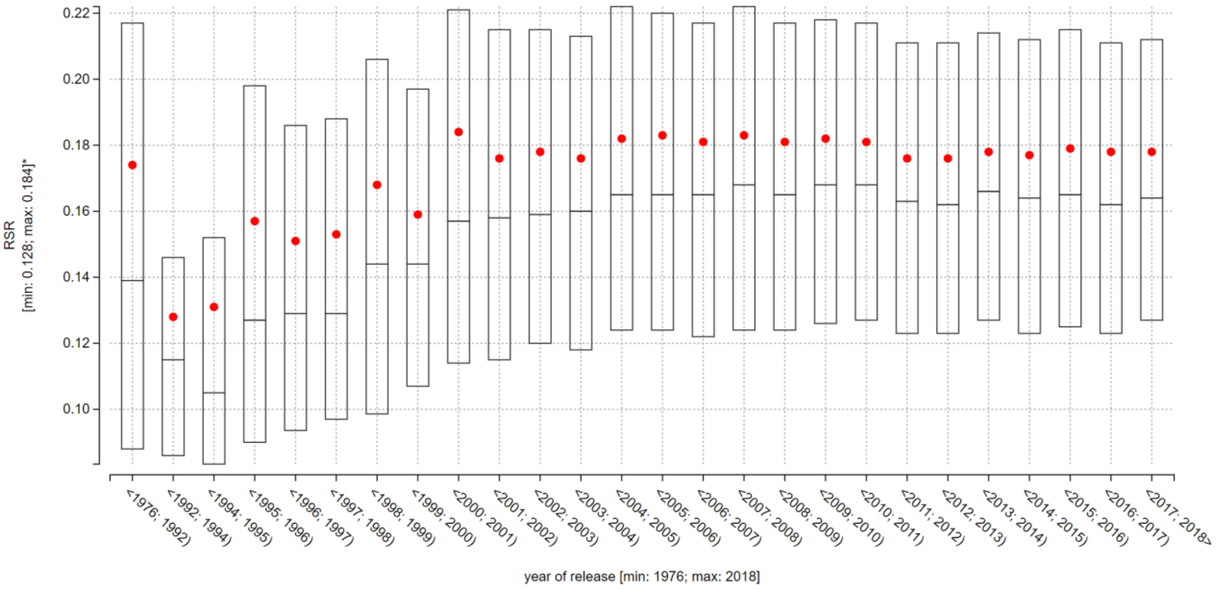

**average RSR of ligands in structure*

Figure S20. Relationship between factors *year of release* and *average RSR of ligands in structure*. Lower values represent higher quality. Spearman's coefficient **R_s = 0.4591**.

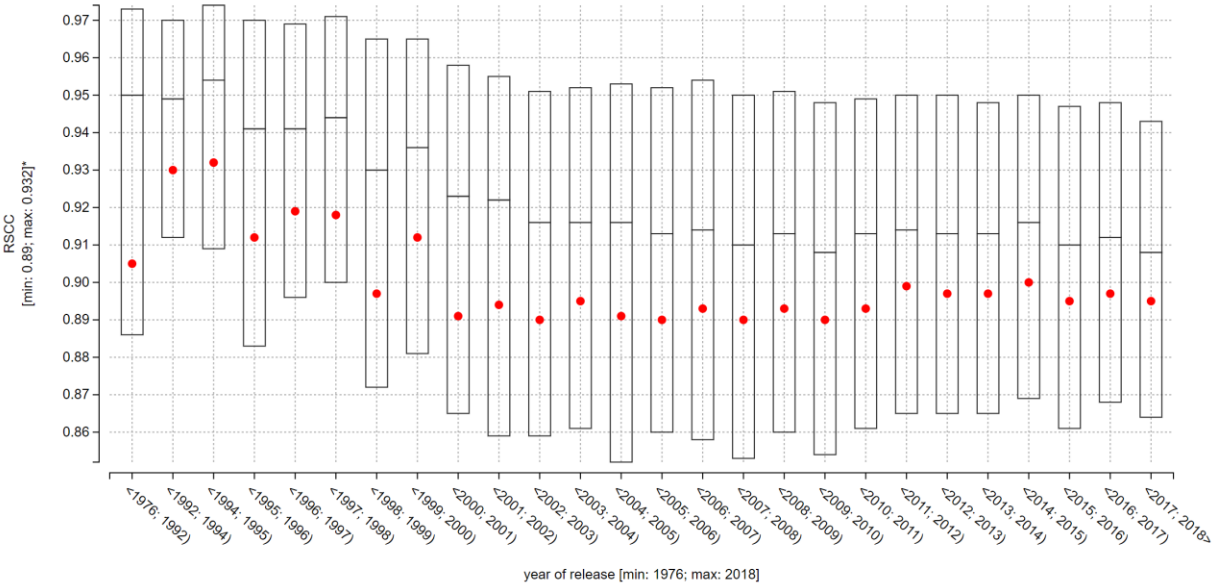

*average ligand RSCC

Figure S21. Relationship between factors *year of release* and *average ligand RSCC*. Higher values represent higher quality. Spearman's coefficient $\mathbf{R_s}$ **= -0.2400**.

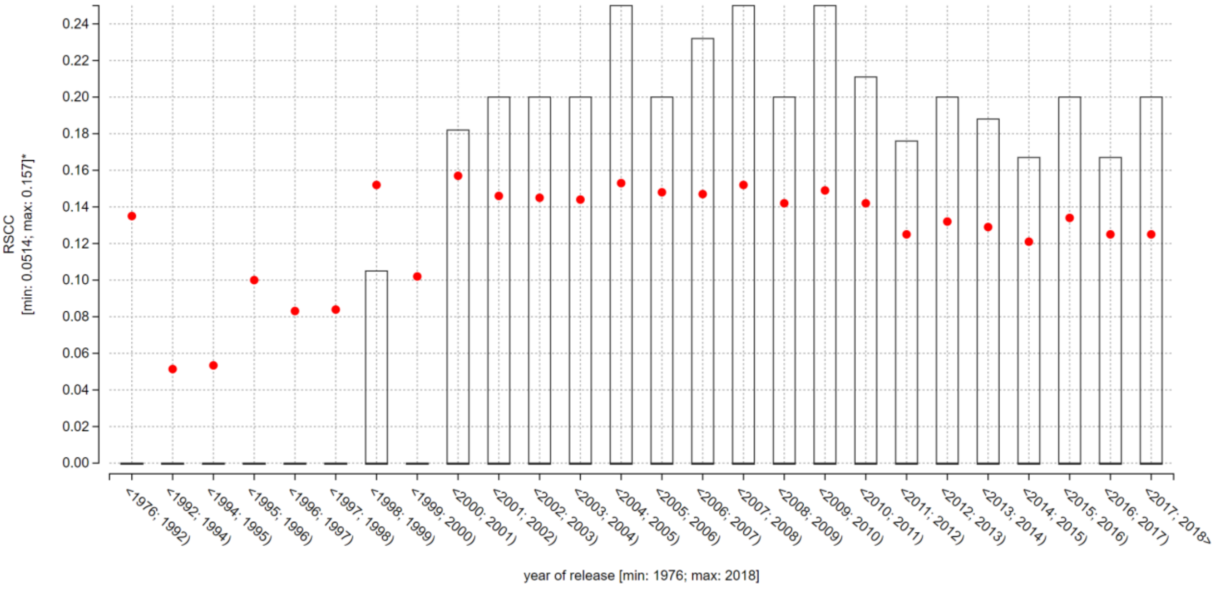

*ratio of RSCC outliers among ligands

Figure S22. Relationship between factors *year of release* and *ratio of RSCC outliers among ligands*. Lower values represent higher quality. Note that all medians and lower quartiles in the plot are equal to 0. Spearman's coefficient $\mathbf{R_s}$ **= 0.0843**.

Trends of size factors in time

Selected plots S23 to S27 show strong relationships between the year of release of PDB structures and factors that quantify size of biomacromolecular complexes in the PDB in various ways. Relationships in these plots clearly demonstrate the trend of PDB structure size increase in time.

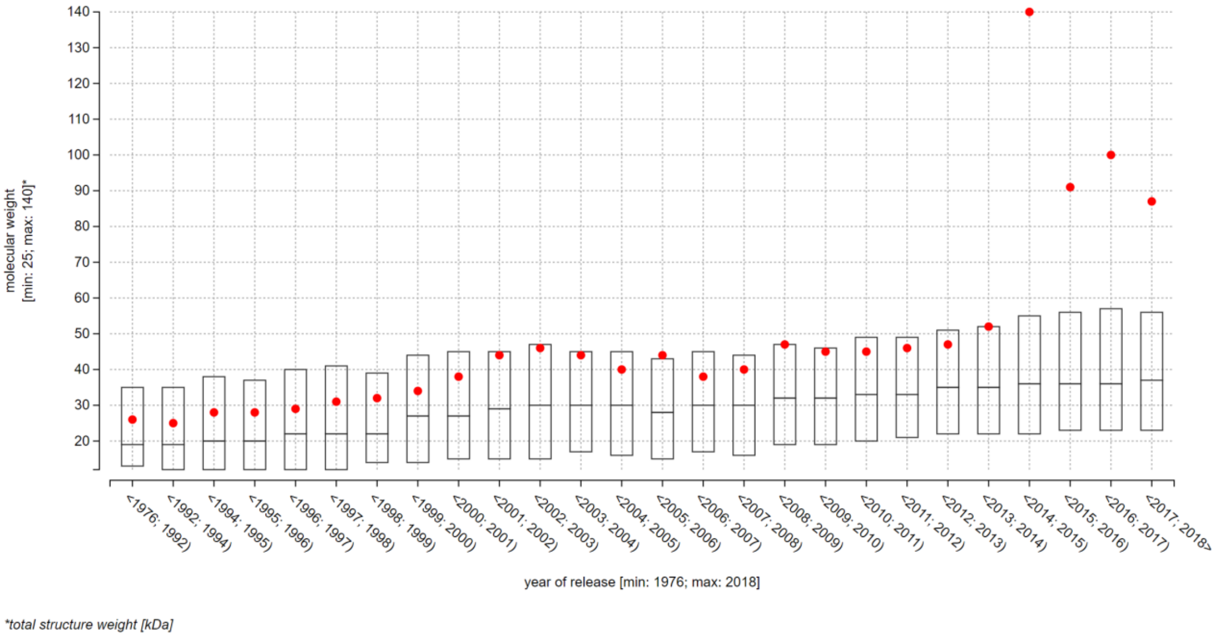

Figure S23. Relationship between factors *year of release* and *total structure weight*. Higher values represent larger structures. Spearman's coefficient $\mathbf{R_s}$ **= 0.9322**.

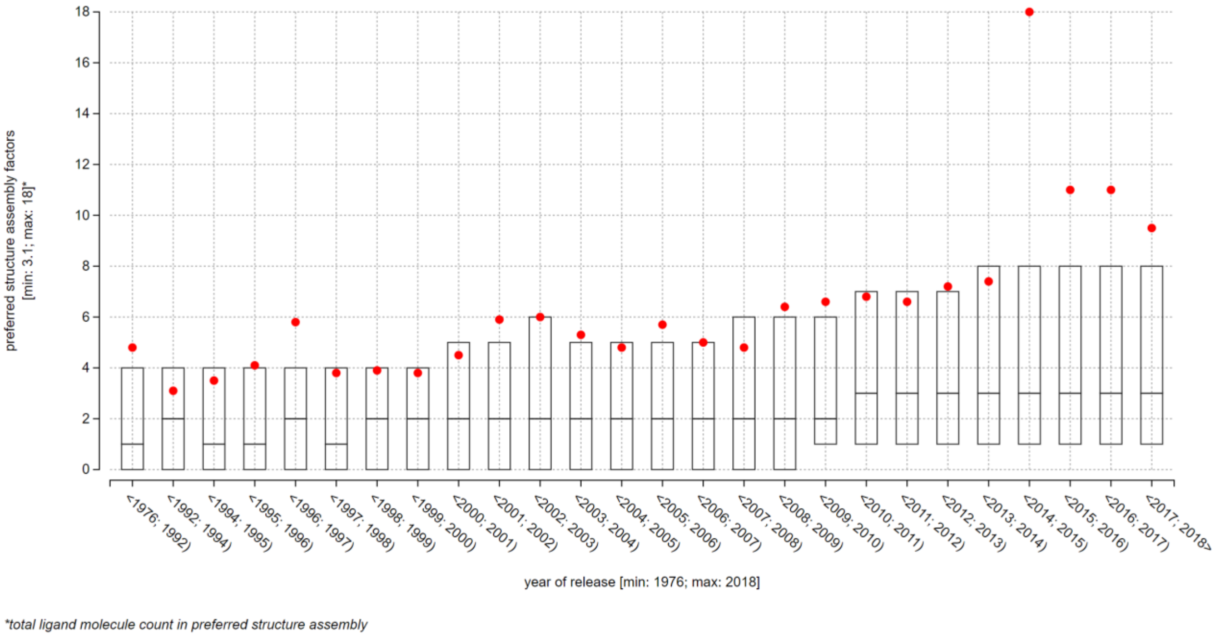

Figure S24. Relationship between factors *year of release* and *total ligand molecule count in preferred structure assembly*. Higher values represent larger structures. Spearman's coefficient $\mathbf{R_s}$ **= 0.8939**.

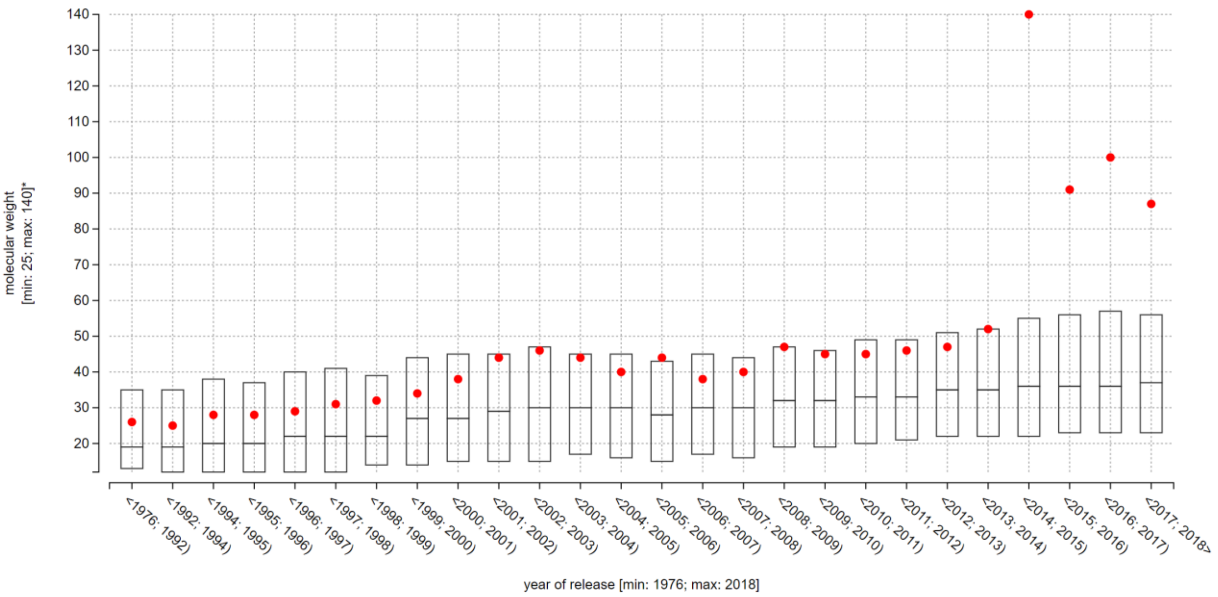

total structure weight [kDa]

Figure S25. Relationship between factors *year of release* and *total structure weight*. Higher values represent larger structures. Spearman's coefficient $\mathbf{R_s}$ **=** **0.9583**.

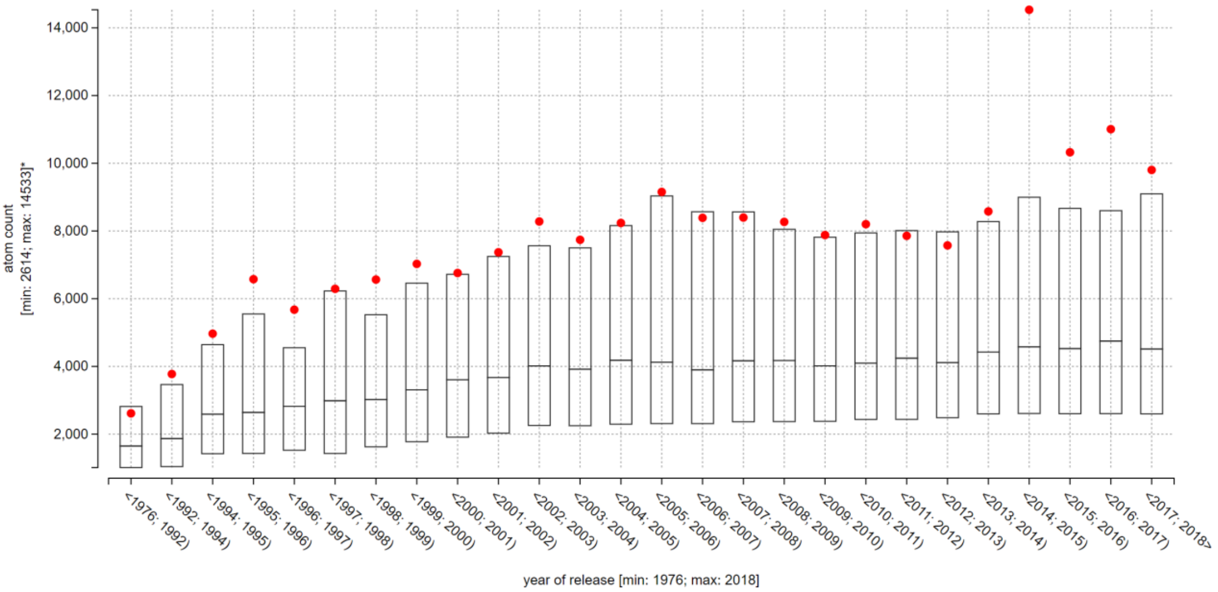

atom count (structure and ligand atoms)

Figure S26. Relationship between factors *year of release* and *atom count (structure and ligand atoms)*. Higher values represent larger structures. Spearman's coefficient $\mathbf{R_s}$ **=** **0.8400**.

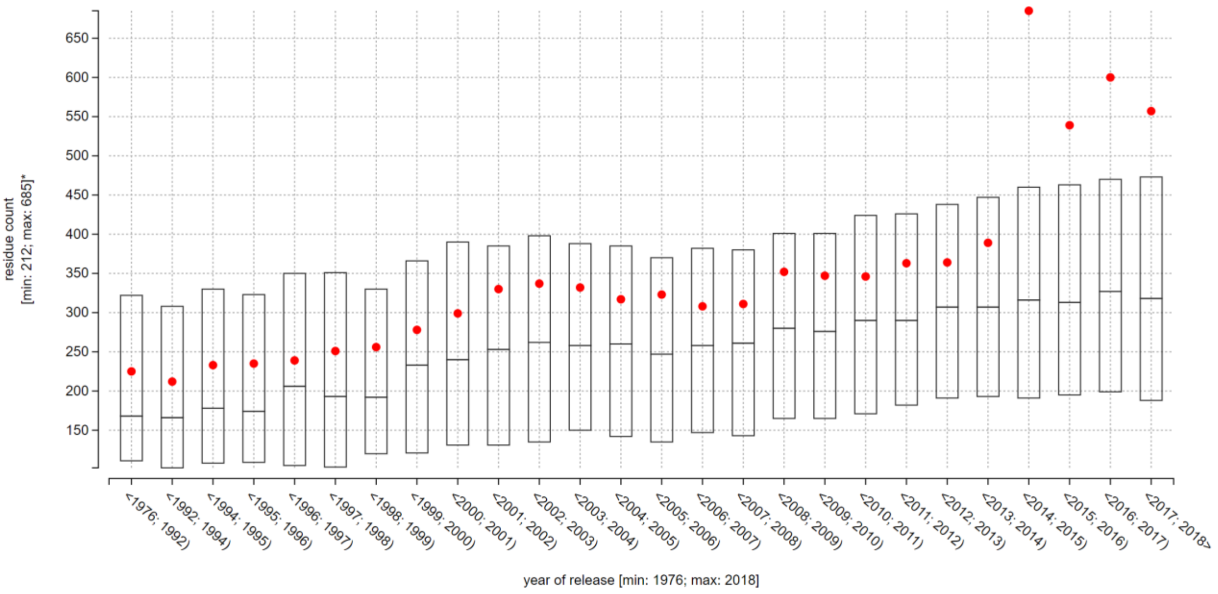

residue and ligand count in structure, disregarding water ligands

Figure S27. Relationship between factors *year of release* and *residue and ligand count in structure, disregarding water ligands*. Higher values represent larger structures. Spearman's coefficient $\mathbf{R_s}$ **= 0.9461**.

Relationships between biomacromolecule structure quality factors and the structure resolution factor

Selected plots S28 to S33 show strong relationships between the resolution of PDB entries, obtained using the X-ray crystallography experimental method, and various quality factors of biomacromolecular structures in PDB entries. These relationships clearly show that geometric quality of biopolymers, as well as their model-to-data fidelity, decreases with decreasing resolution.

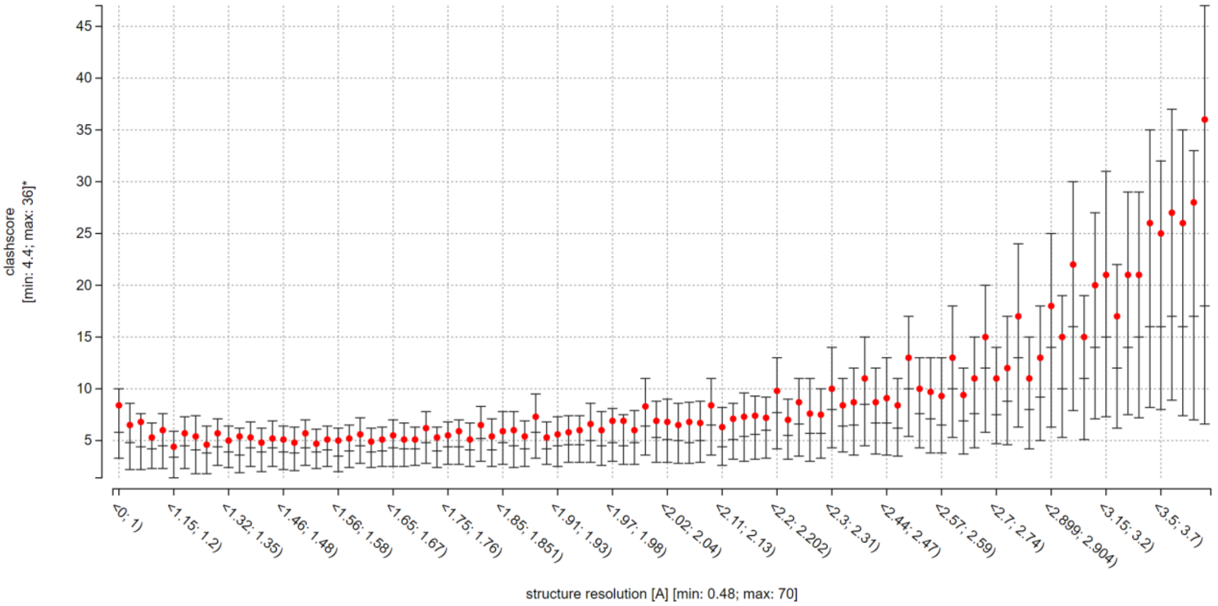

clashscore structure quality factor

Figure S28. Relationship between factors *structure resolution* and *clashscore structure quality factor*. Lower values on Y axis represent higher quality, while lower values on X axis represent higher resolution in Ångströms. Spearman's coefficient $\mathbf{R_s}$ **= 0.8822**.

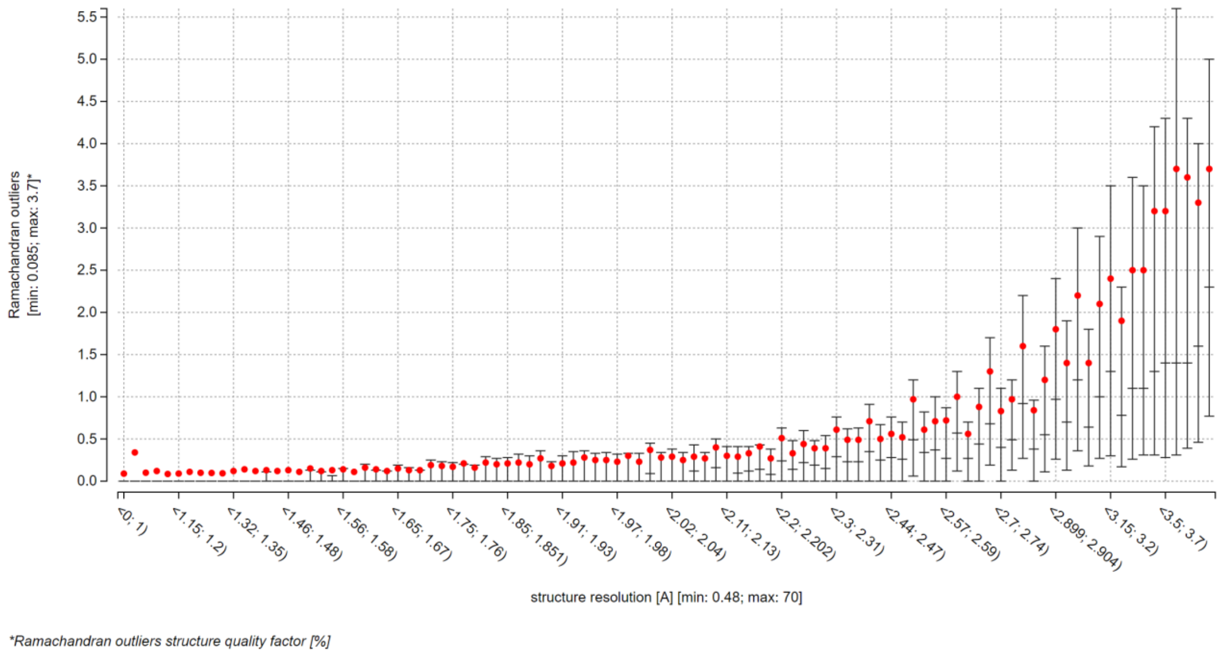

Figure S29. Relationship between factors *structure resolution* and *Ramachandran outliers structure quality factor*. Lower values on Y axis represent higher quality, while lower values on X axis represent higher resolution in Ångströms. Spearman's coefficient $\mathbf{R_s}$ **=** **0.9609**.

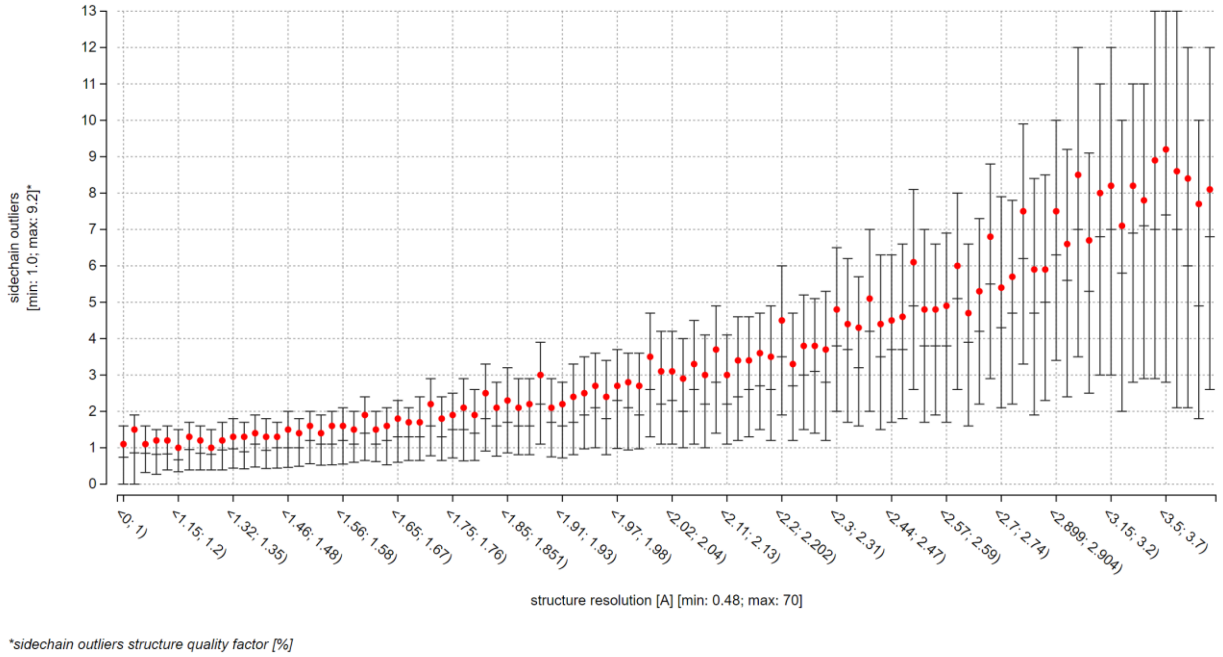

Figure S30. Relationship between factors *structure resolution* and *sidechain outliers structure quality factor*. Lower values on Y axis represent higher quality, while lower values on X axis represent higher resolution in Ångströms. Spearman's coefficient $\mathbf{R_s}$ **=** **0.9865**.

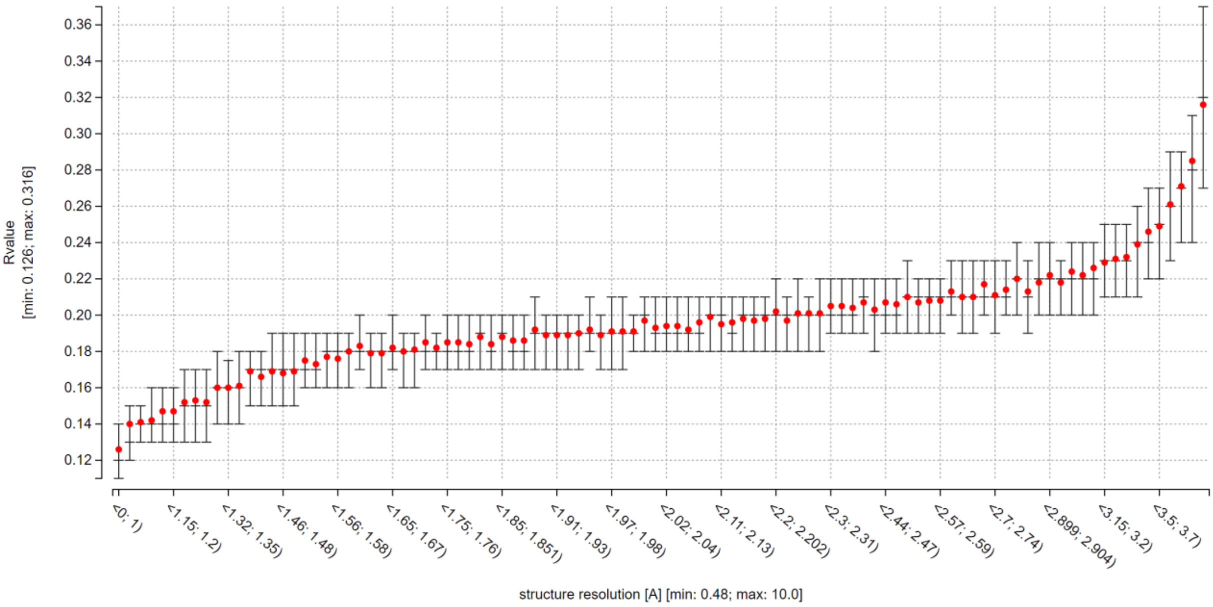

Figure S31. Relationship between factors *structure resolution* and R_{value}. Lower values on Y axis represent higher quality, while lower values on X axis represent higher resolution in Ångströms. Spearman's coefficient $\mathbf{R_s}$ **=** **0.9960**.

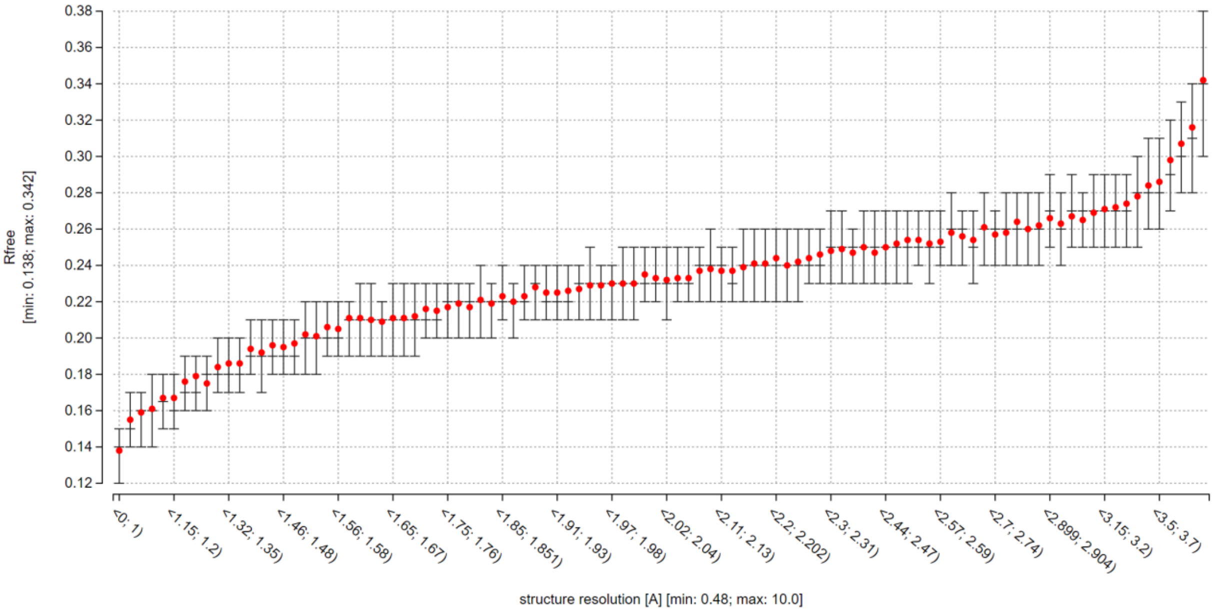

Figure S32. Relationship between factors *structure resolution* and R_{free}. Lower values on Y axis represent higher quality, while lower values on X axis represent higher resolution in Ångströms. Spearman's coefficient $\mathbf{R_s}$ **=** **0.9981**.

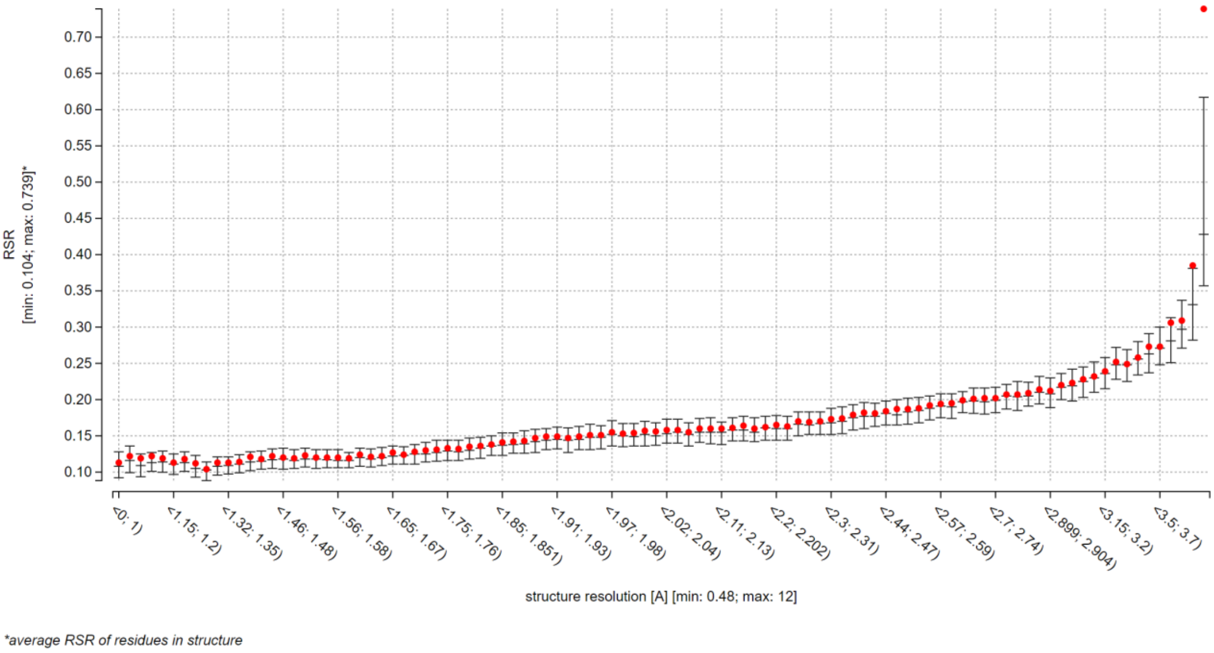

Figure S33. Relationship between factors *structure resolution* and *average RSR of residues in structure*. Lower values on Y axis represent higher quality, while lower values on X axis represent higher resolution in Ångströms. Spearman's coefficient $\mathbf{R_s}$ **=** **0.9896**.

Relationships between ligand quality factors and the structure resolution factor

Selected plots S34 to S36 show strong or moderate relationships between the resolution of PDB entries, obtained using the X-ray crystallography experimental method, and various ligand quality factors. These relationships clearly show that geometric quality of ligands in PDB entries, as well as their model-to-data fidelity, decreases with decreasing resolution.

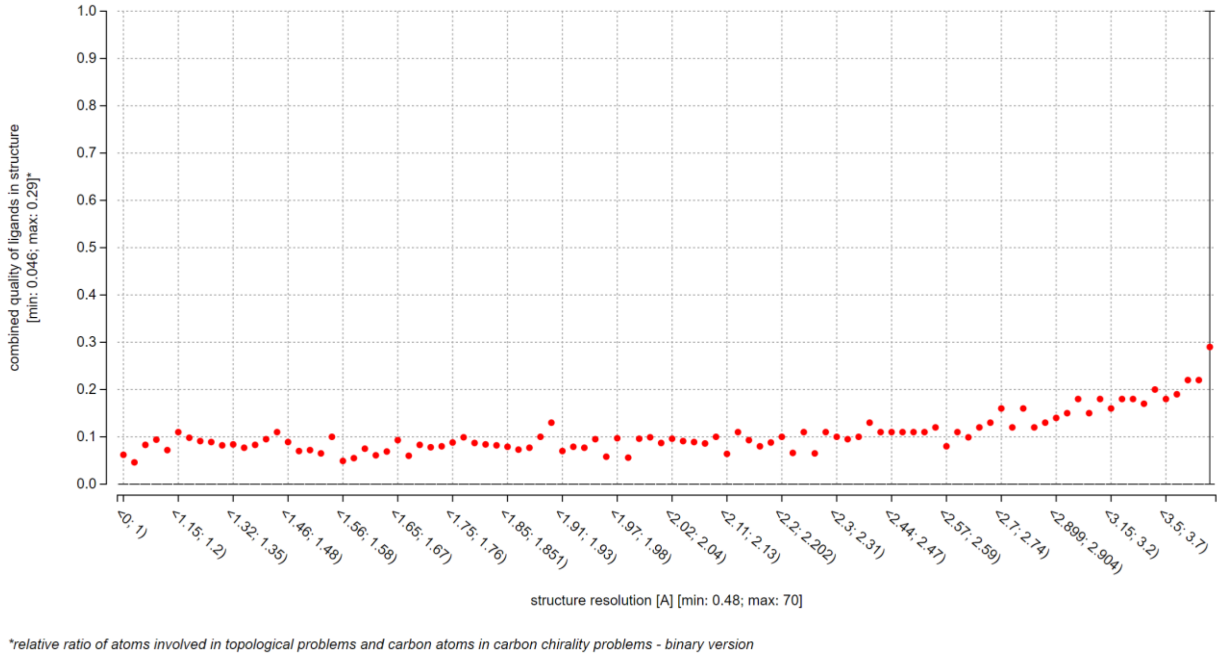

Figure S34. Relationship between factors *structure resolution* and *relative ratio of atoms involved in topological problems and carbon atoms in carbon chirality problems - binary version*. Lower values on Y axis represent higher quality, while lower values on X axis represent higher resolution in Ångströms. Note that all medians and lower quartiles in the plot are equal to 0. Spearman's coefficient $\mathbf{R_s}$ **=** **0.6740**.

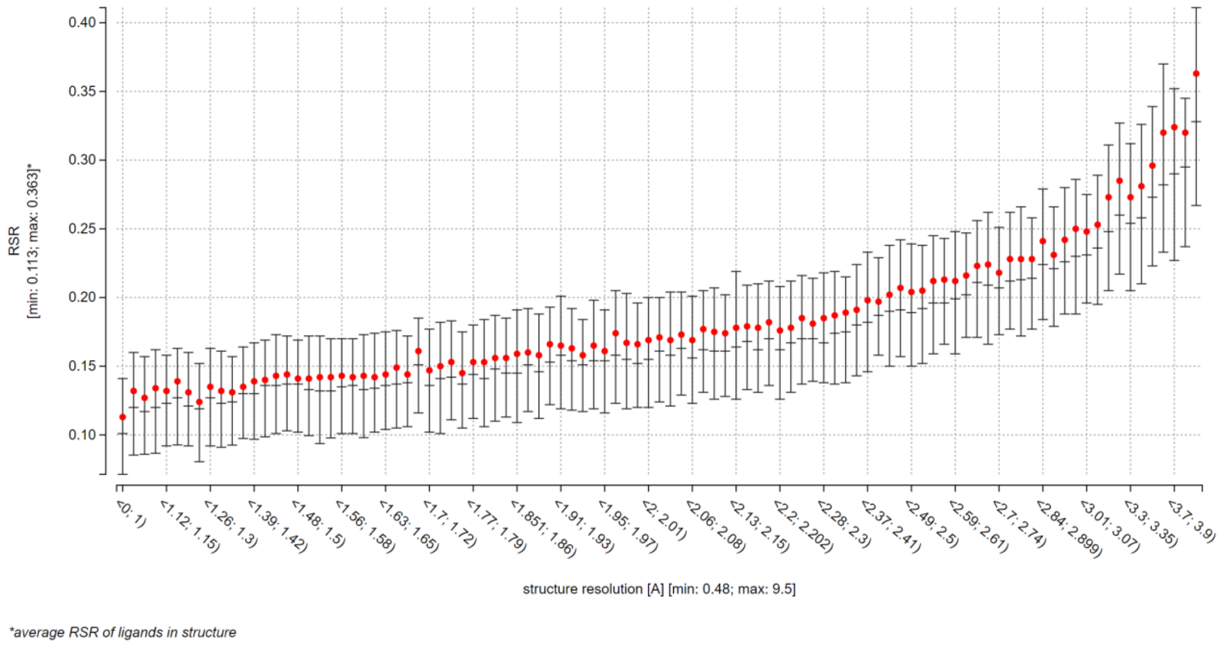

Figure S35. Relationship between factors *structure resolution* and *average RSR of ligands in structure*. Lower values on Y axis represent higher quality, while lower values on X axis represent higher resolution in Ångströms. Spearman's coefficient $\mathbf{R_s}$ **=** **0.9930**.

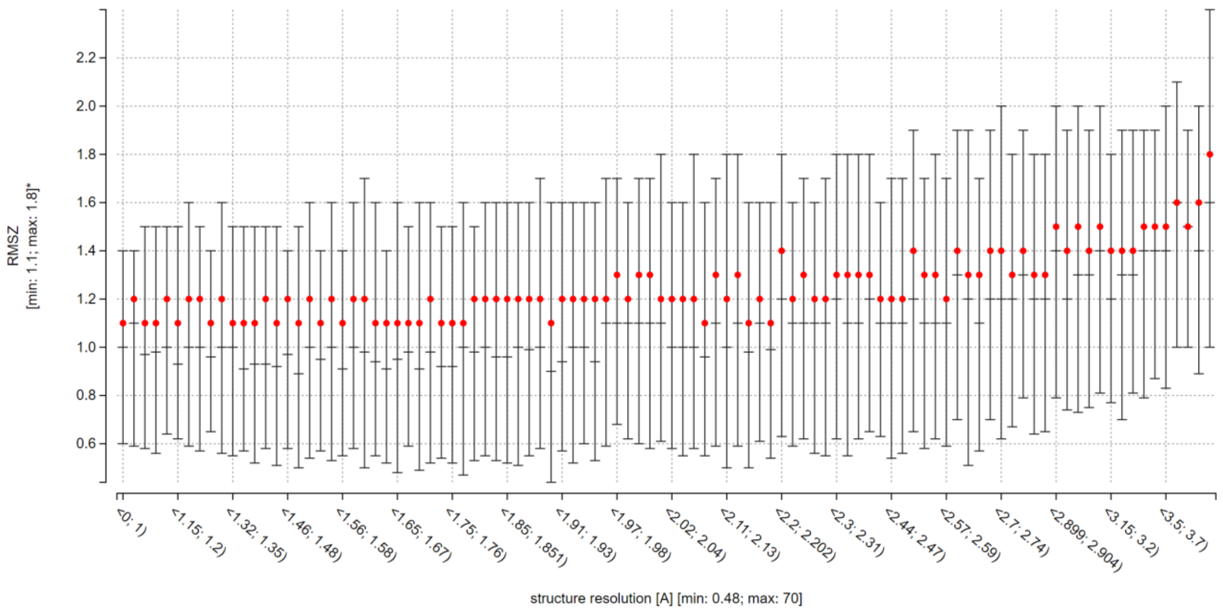

average RMSZ of bond angles in ligands

Figure S36. Relationship between factors *structure resolution* and *average RMSZ of bond angles in ligands*. Lower values on Y axis represent higher quality, while lower values on X axis represent higher resolution in Ångströms. Spearman's coefficient $\mathbf{R_s}$ **= 0.8184**.

Relationships between biomacromolecule structure quality factors and size factors

Selected plots S37 to S49 show strong or moderate relationships between factors that quantify size of biomacromolecular complexes in the PDB in various ways and factors that represent a number of quality facets of biomacromolecular structures. These relationships clearly show that with increase in size of PDB entries comes decrease in quality of their biomacromolecular components.

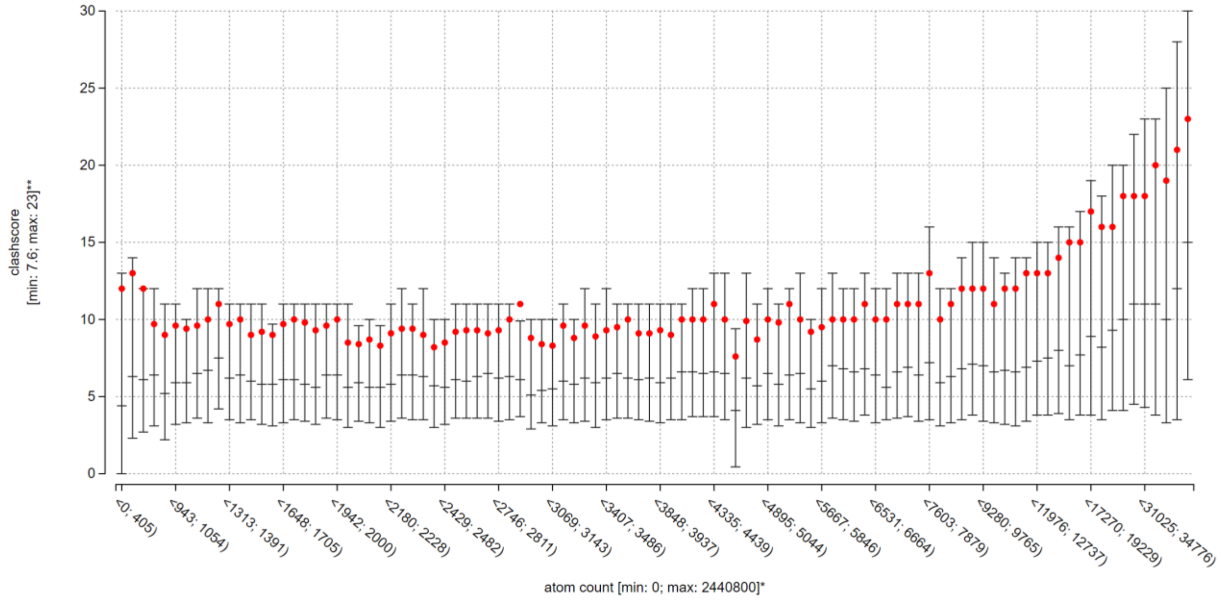

**structure atom count (without ligand atoms)*
***clashscore structure quality factor*

Figure S37. Relationship between factors *structure atom count (without ligand atoms)* and *clashscore structure quality factor*. Lower values on Y axis represent higher quality, while higher values on X axis represent larger structures. Spearman's coefficient $\mathbf{R_s}$ **= 0.6247**.

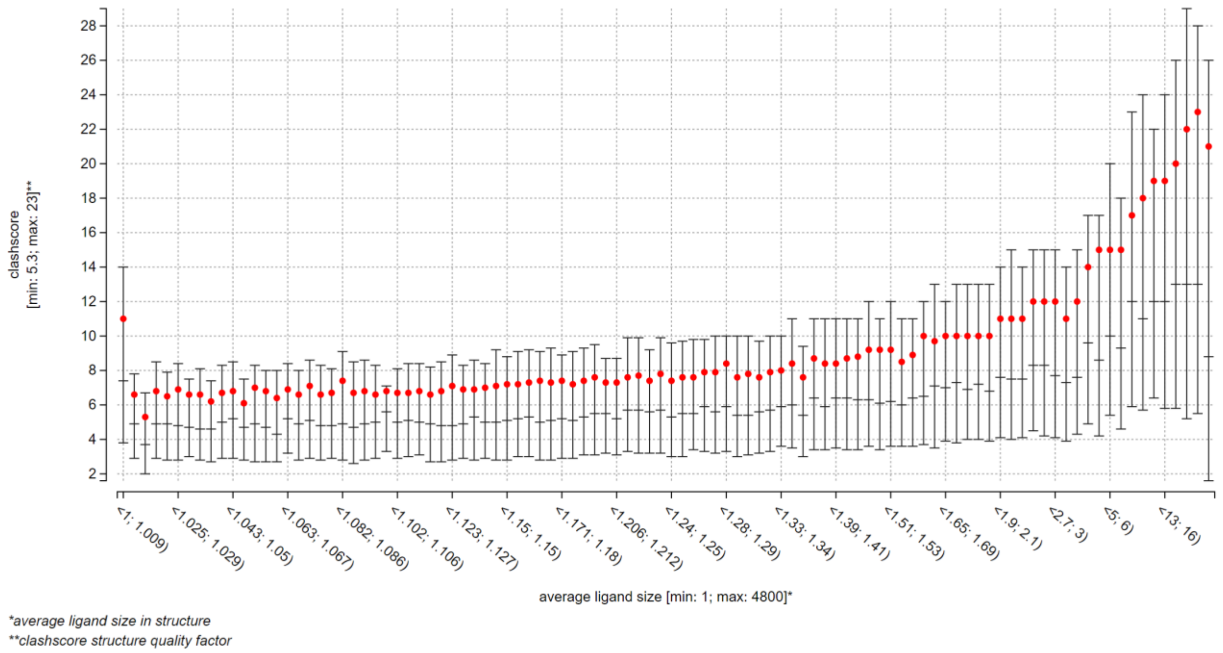

Figure S38. Relationship between factors *average ligand size in structure* and *clashscore structure quality factor*. Lower values on Y axis represent higher quality, while higher values on X axis represent larger structures. Spearman's coefficient $\mathbf{R_s}$ **= 0.9144**.

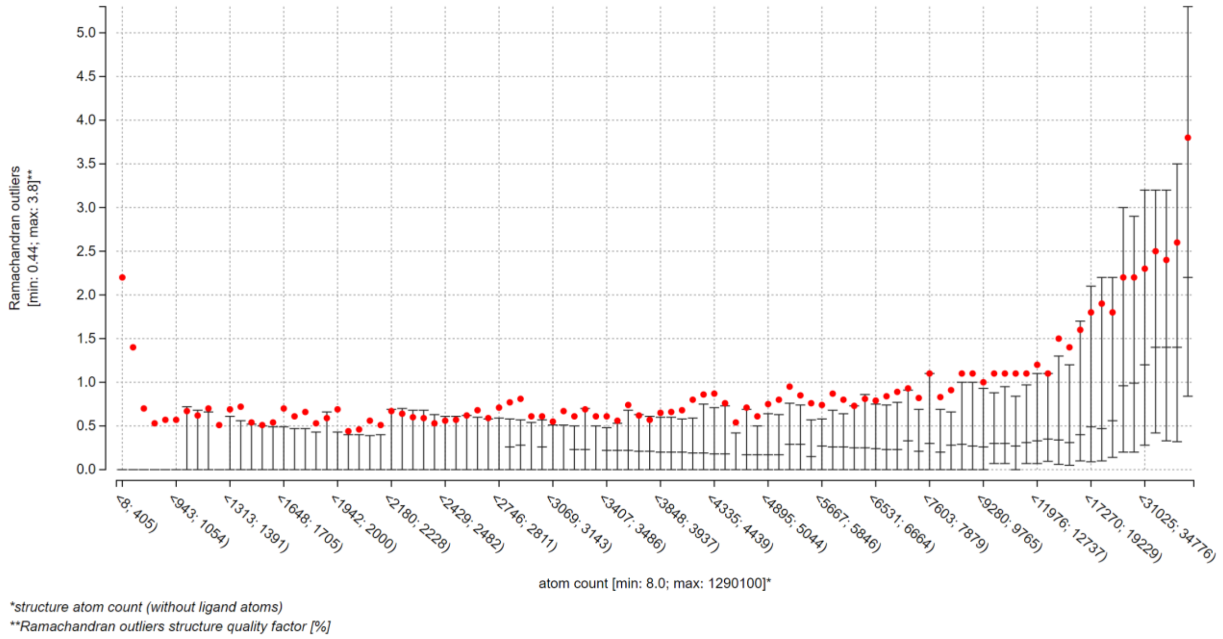

Figure S39. Relationship between factors *structure atom count (without ligand atoms)* and *Ramachandran outliers structure quality factor*. Lower values on Y axis represent higher quality, while higher values on X axis represent larger structures. Spearman's coefficient $\mathbf{R_s}$ **= 0.8064**.

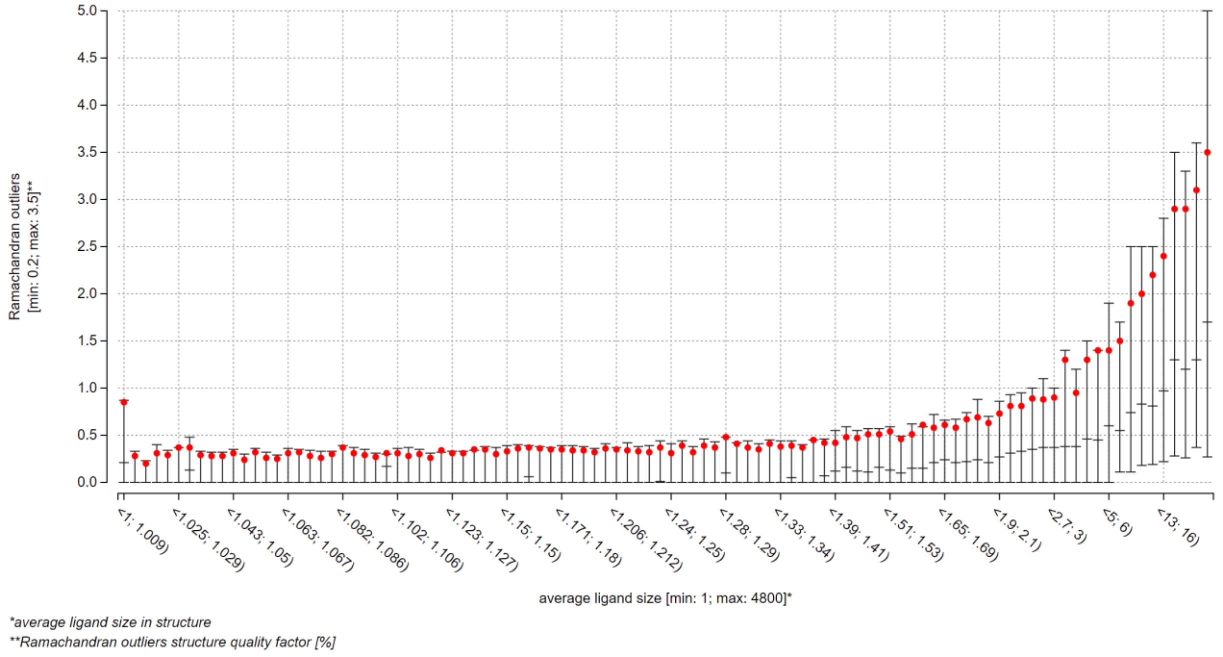

Figure S40. Relationship between factors *average ligand size in structure* and *Ramachandran outliers structure quality factor*. Lower values on Y axis represent higher quality, while higher values on X axis represent larger structures. Spearman's coefficient $\mathbf{R_s}$ **=** <u>**0.8523**</u>.

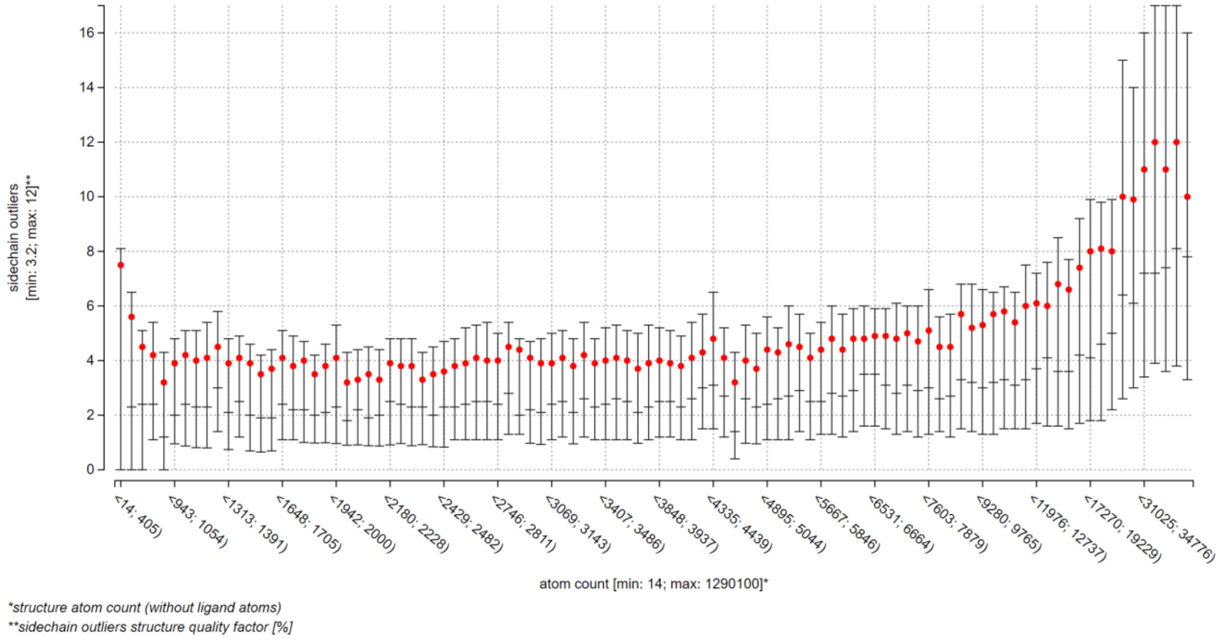

Figure S41. Relationship between factors *structure atom count (without ligand atoms)* and *sidechain outliers structure quality factor*. Lower values on Y axis represent higher quality, while higher values on X axis represent larger structures. Spearman's coefficient $\mathbf{R_s}$ **=** <u>**0.7377**</u>.

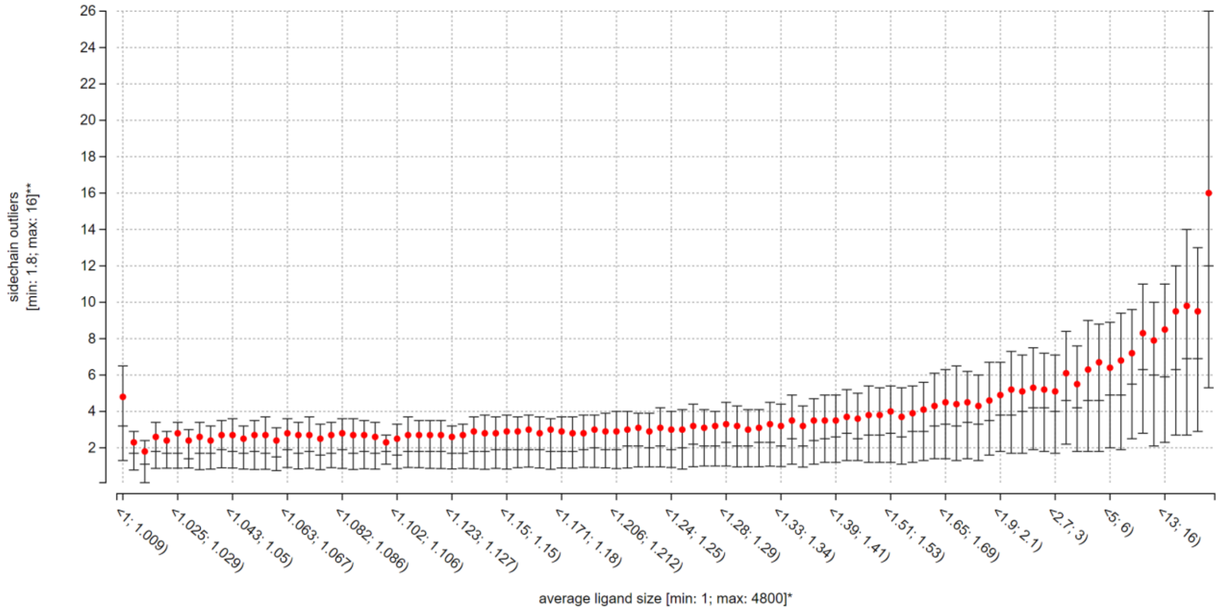

**average ligand size in structure*
***sidechain outliers structure quality factor [%]*

Figure S42. Relationship between factors *average ligand size in structure* and *sidechain outliers structure quality factor*. Lower values on Y axis represent higher quality, while higher values on X axis represent larger structures. Spearman's coefficient $\mathbf{R_s}$ **=** **0.9132**.

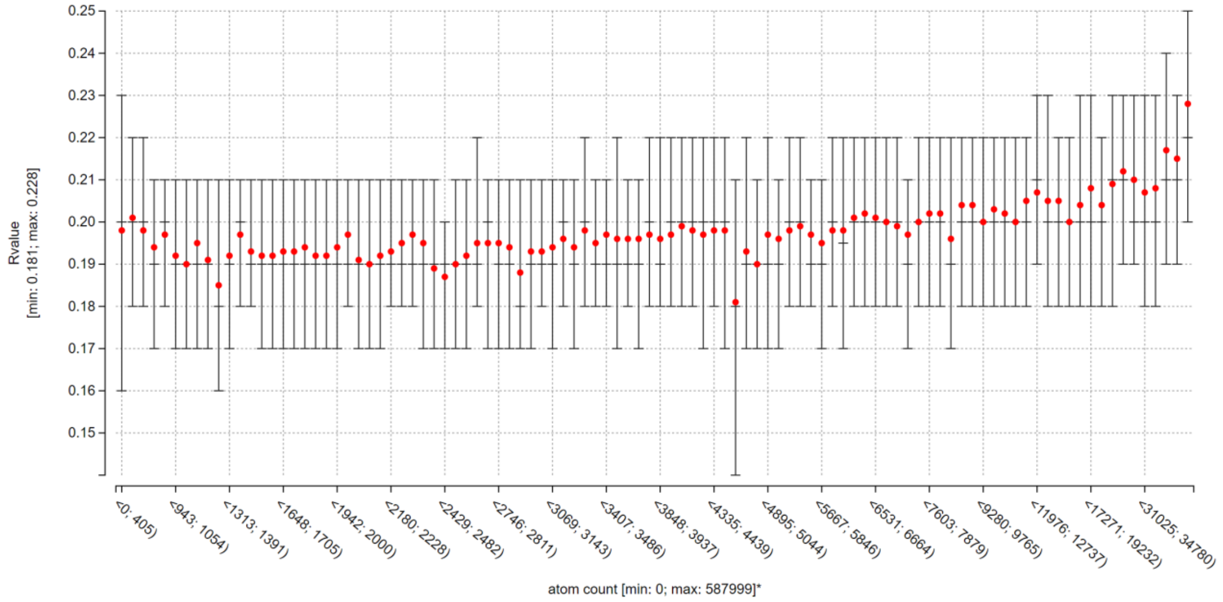

**structure atom count (without ligand atoms)*

Figure S43. Relationship between factors *structure atom count (without ligand atoms)* and R_{value}. Lower values on Y axis represent higher quality, while higher values on X axis represent larger structures. Spearman's coefficient $\mathbf{R_s}$ **=** **0.7711**.

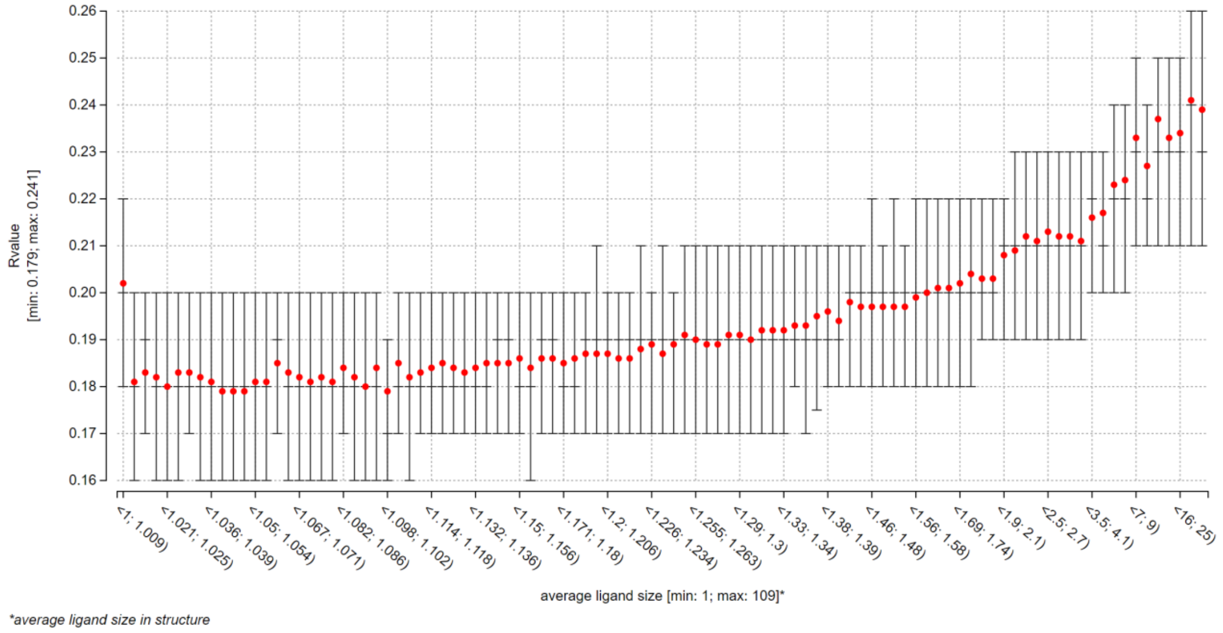

Figure S44. Relationship between factors *average ligand size in structure* and R_{value}. Lower values on Y axis represent higher quality, while higher values on X axis represent larger structures. Spearman's coefficient $\mathbf{R_s}$ **=** **0.9245**.

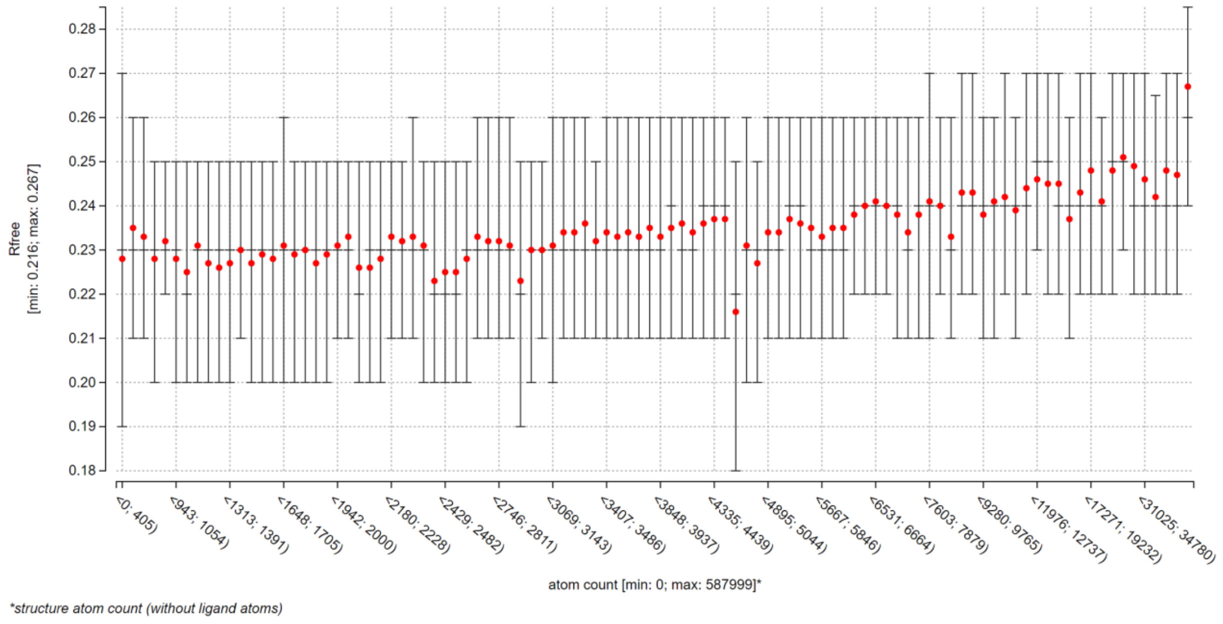

Figure S45. Relationship between factors *structure atom count (without ligand atoms)* and R_{free}. Lower values on Y axis represent higher quality, while higher values on X axis represent larger structures. Spearman's coefficient $\mathbf{R_s}$ **=** **0.8362**.

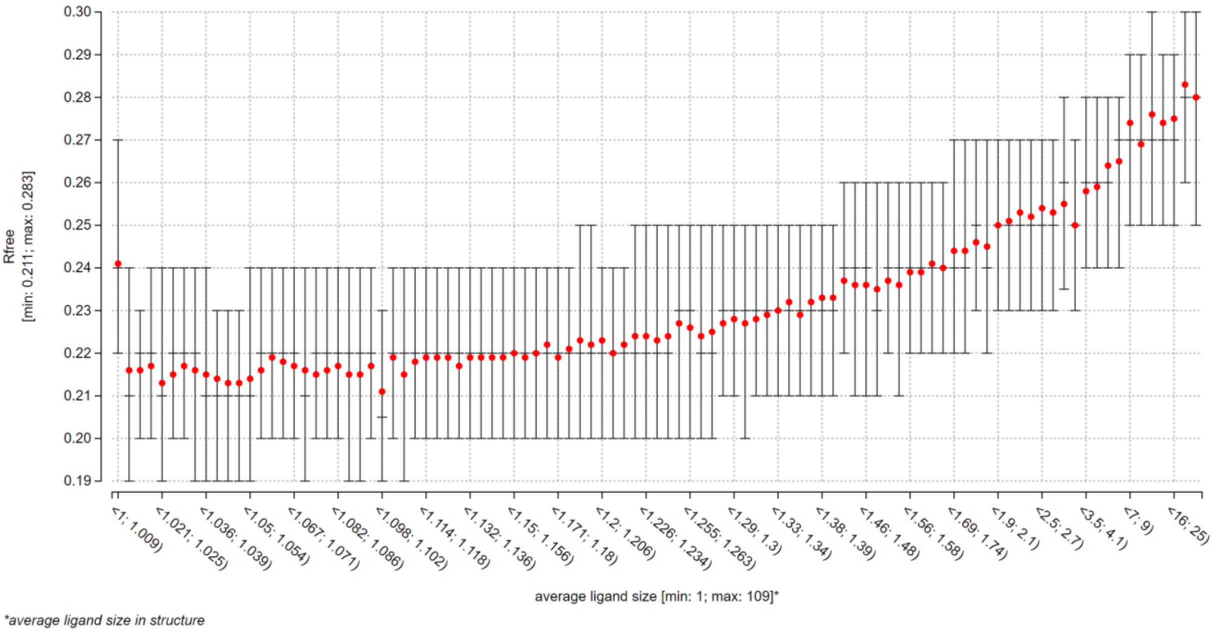

Figure S46. Relationship between factors *average ligand size in structure* and R_{free}. Lower values on Y axis represent higher quality, while higher values on X axis represent larger structures. Spearman's coefficient $\mathbf{R_s}$ **=** **0.9271**.

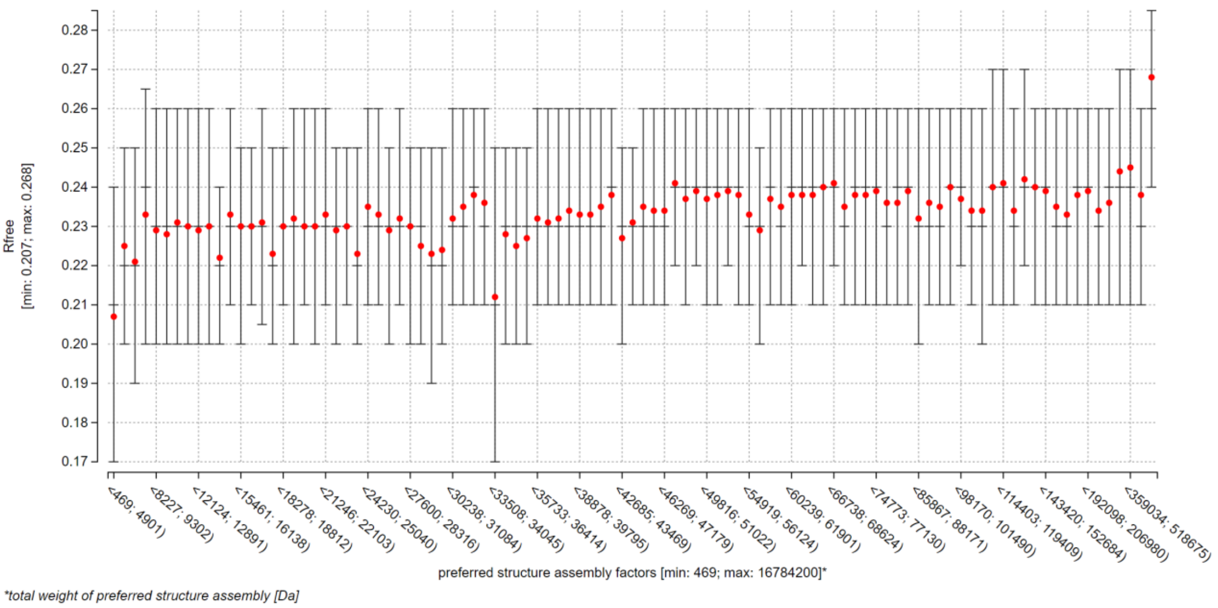

Figure S47. Relationship between factors *average ligand size in structure* and total weight of preferred structure assembly. Lower values on Y axis represent higher quality, while higher values on X axis represent larger structures. Spearman's coefficient $\mathbf{R_s}$ **=** **0.7226**.

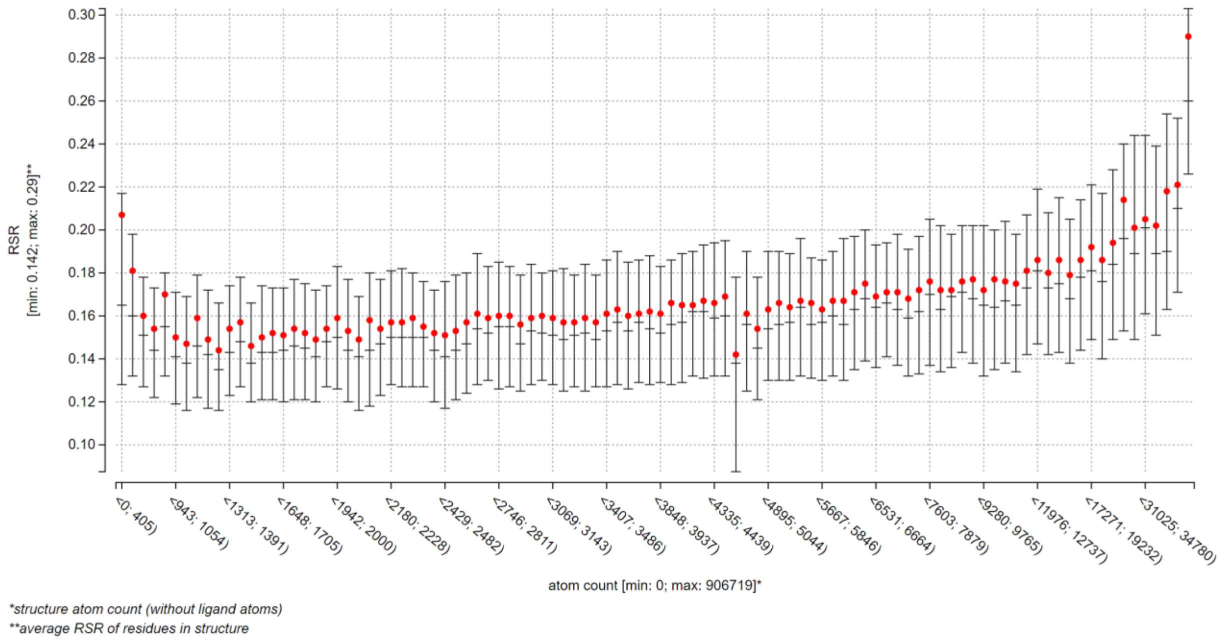

Figure S48. Relationship between factors *structure atom count (without ligand atoms)* and *average RSR of residues in structure*. Lower values on Y axis represent higher quality, while higher values on X axis represent larger structures. Spearman's coefficient $\mathbf{R_s}$ **= 0.8718**.

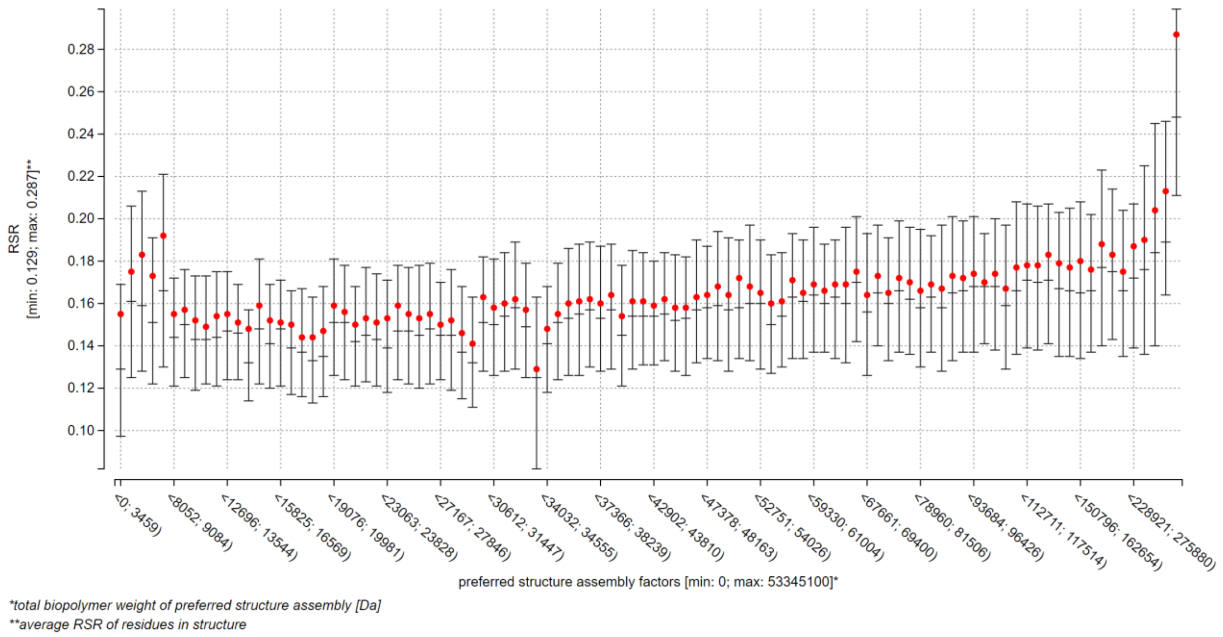

Figure S49. Relationship between factors *total biopolymer weight of preferred structure assembly* and *average RSR of residues in structure*. Lower values on Y axis represent higher quality, while higher values on X axis represent larger structures. Spearman's coefficient $\mathbf{R_s}$ **= 0.6828**.

Relationships between ligand quality factors and size factors

Selected plots S50 to S65 show strong or moderate relationships between factors that quantify the amount of ligands in PDB entries in various ways and factors that represent several facets of ligand quality. These relationships clearly demonstrate the trend that that ligand quality tends to decrease with increase of the total amount of ligands in PDB entries, while it also tends to decrease with increasing size of said ligands in PDB entries.

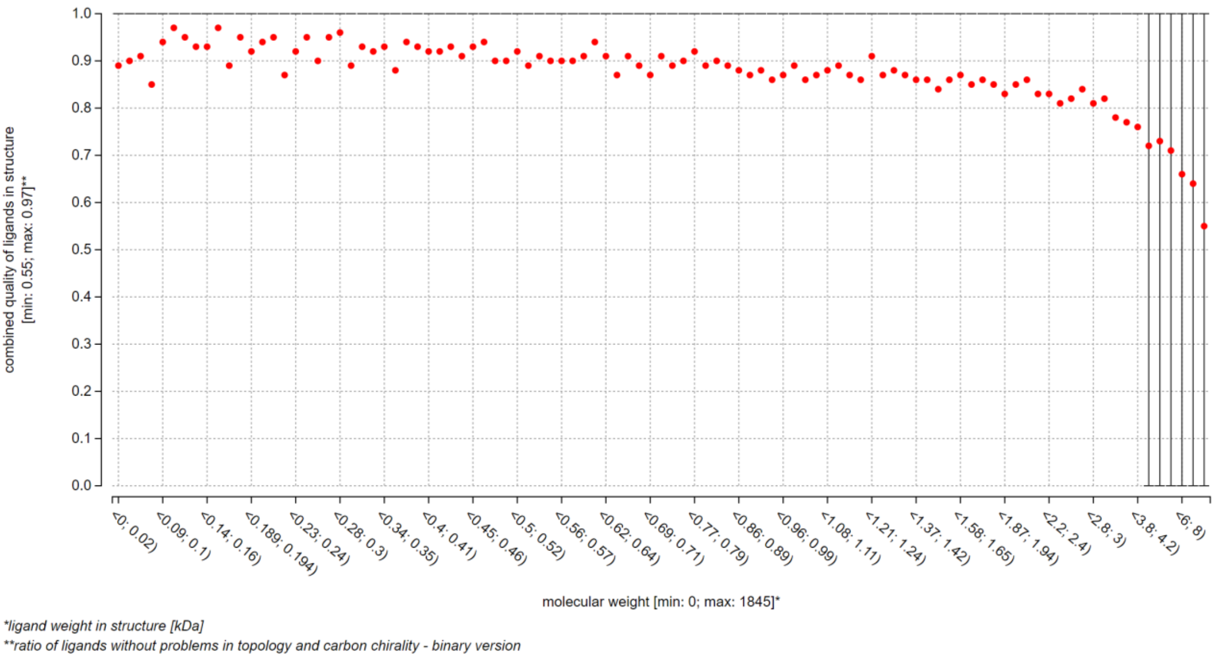

Figure S50. Relationship between factors *ligand weight in structure* and *ratio of ligands without problems in topology and carbon chirality - binary version*. Higher values on Y axis represent higher quality, while higher values on X axis represent larger structures. Note that all medians and upper quartiles in the plot are equal to 1. Spearman's coefficient $\mathbf{R_s}$ **= -0.8019**.

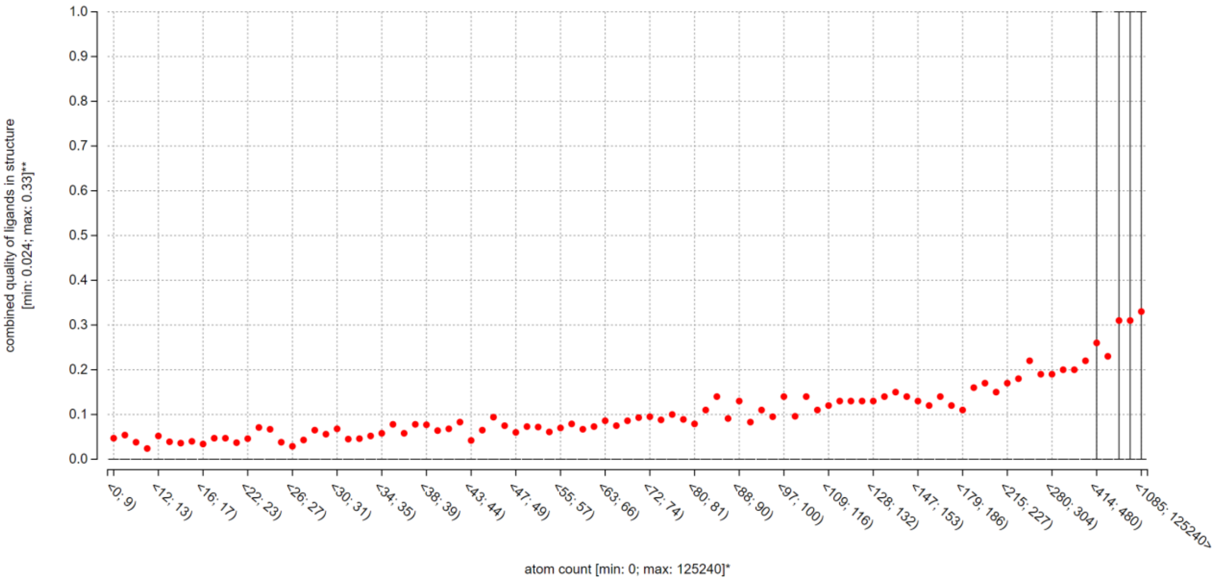

*atom count of ligands in structure, disregarding water ligands
**relative ratio of atoms involved in topological problems and carbon atoms in carbon chirality problems - binary version

Figure S51. Relationship between factors *atom count of ligands in structure, disregarding water ligands* and *relative ratio of atoms involved in topological problems and carbon atoms in carbon chirality problems - binary version*. Lower values on Y axis represent higher quality, while higher values on X axis represent larger structures. Note that all medians and lower quartiles in the plot are equal to 0. Spearman's coefficient $\mathbf{R_s}$ **=** **0.9410**.

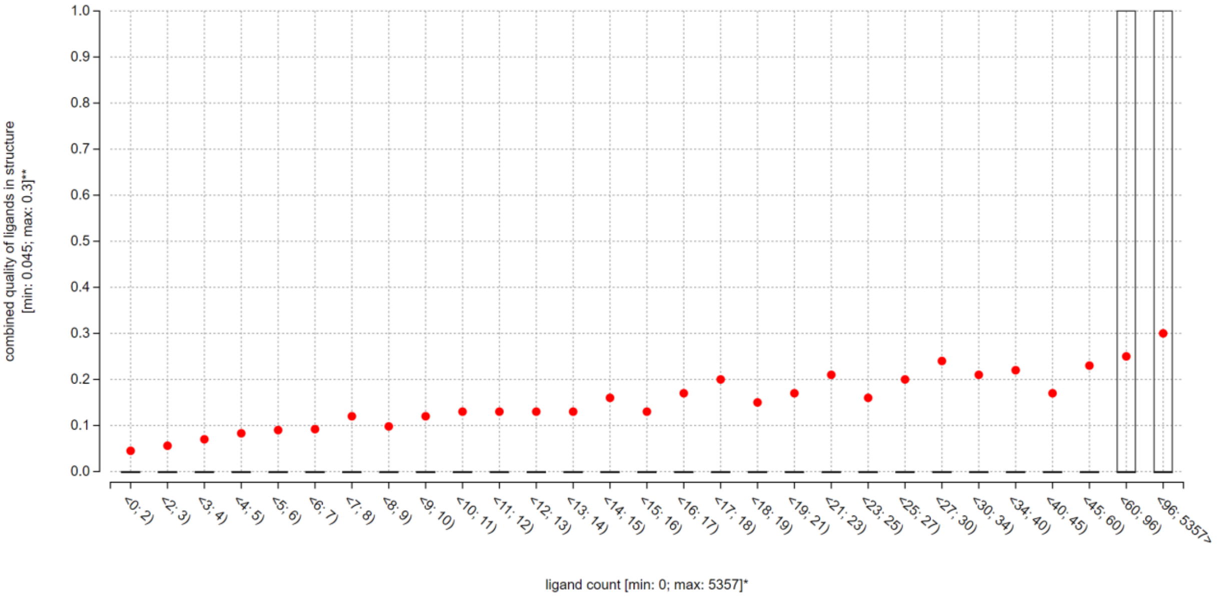

*ligand count in structure, disregarding water ligands
**relative ratio of atoms involved in topological problems and carbon atoms in carbon chirality problems - binary version

Figure S52. Relationship between factors *ligand count in structure, disregarding water ligands* and *relative ratio of atoms involved in topological problems and carbon atoms in carbon chirality problems - binary version*. Lower values on Y axis represent higher quality, while higher values on X axis represent larger structures. Note that all medians and lower quartiles in the plot are equal to 0. Spearman's coefficient $\mathbf{R_s}$ **=** **0.9608**.

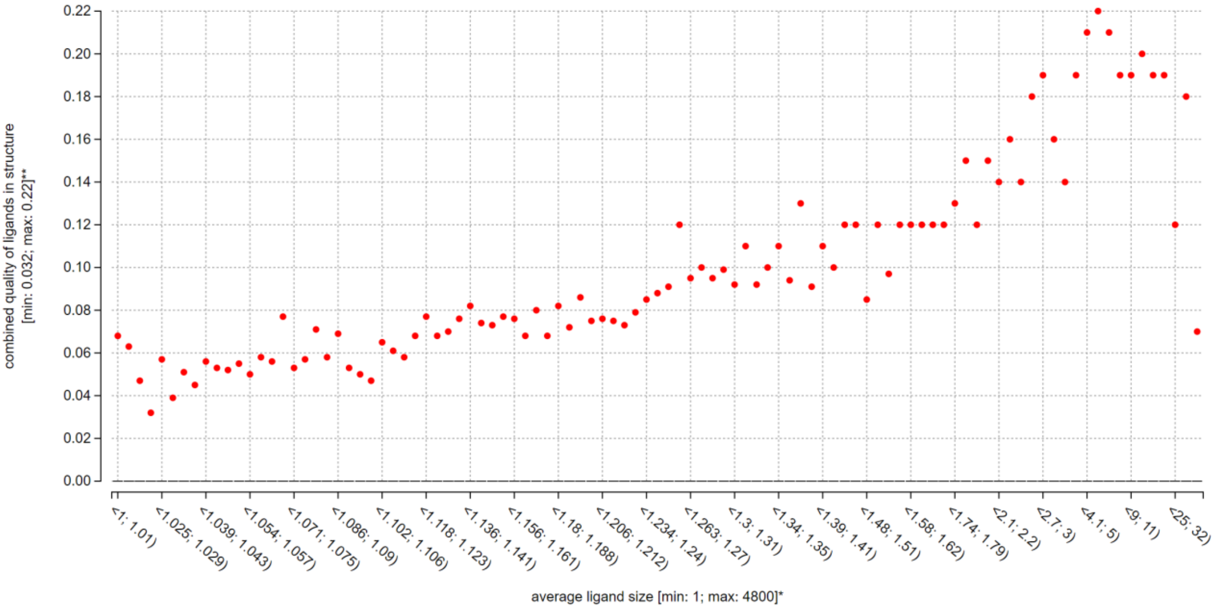

**average ligand size in structure*
***relative ratio of atoms involved in topological problems and carbon atoms in carbon chirality problems - binary version*

Figure S53. Relationship between factors *average ligand size in structure* and *relative ratio of atoms involved in topological problems and carbon atoms in carbon chirality problems - binary version*. Lower values on Y axis represent higher quality, while higher values on X axis represent larger structures. Note that all medians and both quartiles in the plot are equal to 0. Spearman's coefficient **R_s = 0.9490**.

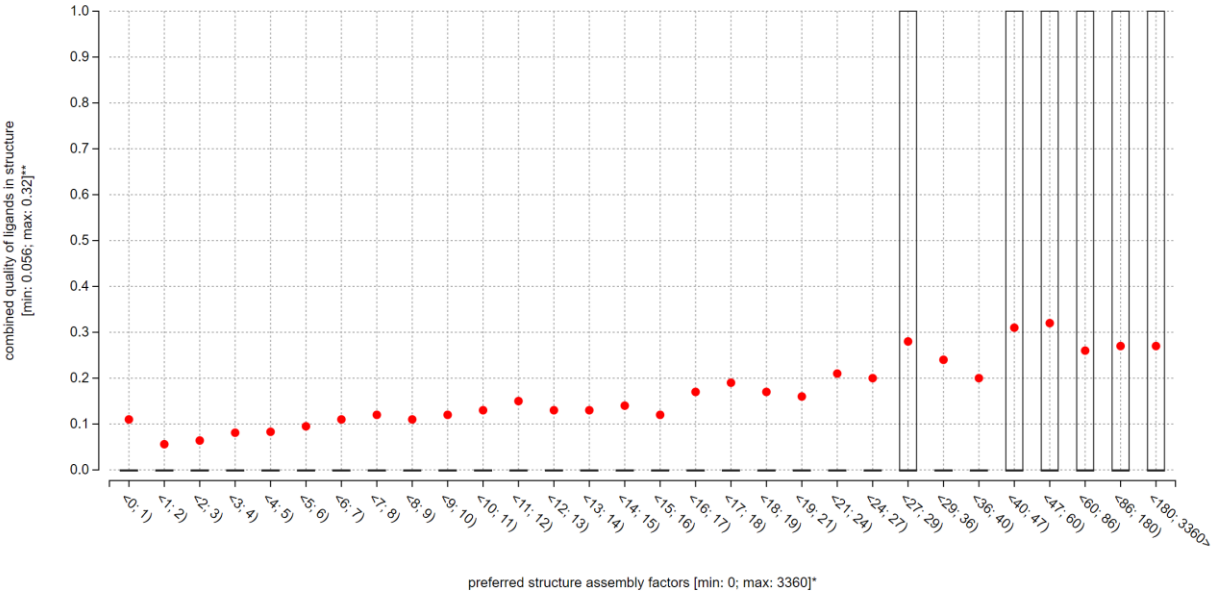

**total ligand molecule count in preferred structure assembly*
***relative ratio of atoms involved in topological problems and carbon atoms in carbon chirality problems - binary version*

Figure S54. Relationship between factors *total ligand molecule count in preferred structure assembly* and *relative ratio of atoms involved in topological problems and carbon atoms in carbon chirality problems - binary version*. Lower values on Y axis represent higher quality, while higher values on X axis represent larger structures. Note that all medians and lower quartiles in the plot are equal to 0. Spearman's coefficient **R_s = 0.9615**.

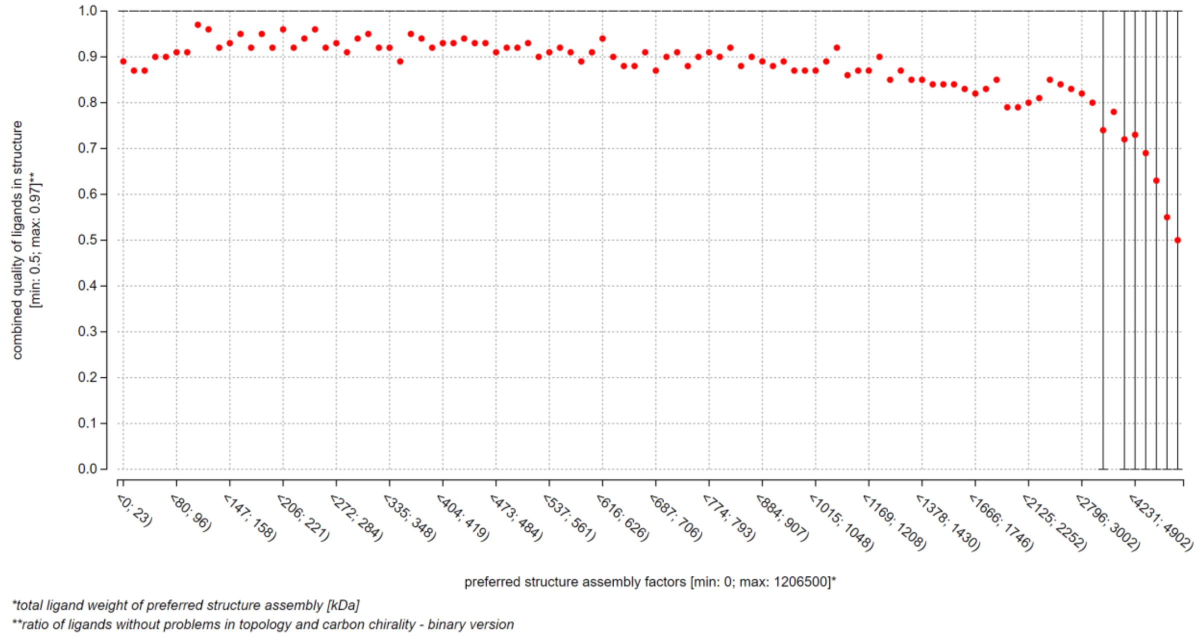

Figure S55. Relationship between factors *total ligand weight of preferred structure assembly* and *ratio of ligands without problems in topology and carbon chirality - binary version*. Higher values on Y axis represent higher quality, while higher values on X axis represent larger structures. Note that all medians and upper quartiles in the plot are equal to 1. Spearman's coefficient $\mathbf{R_s}$ **= -0.7711**.

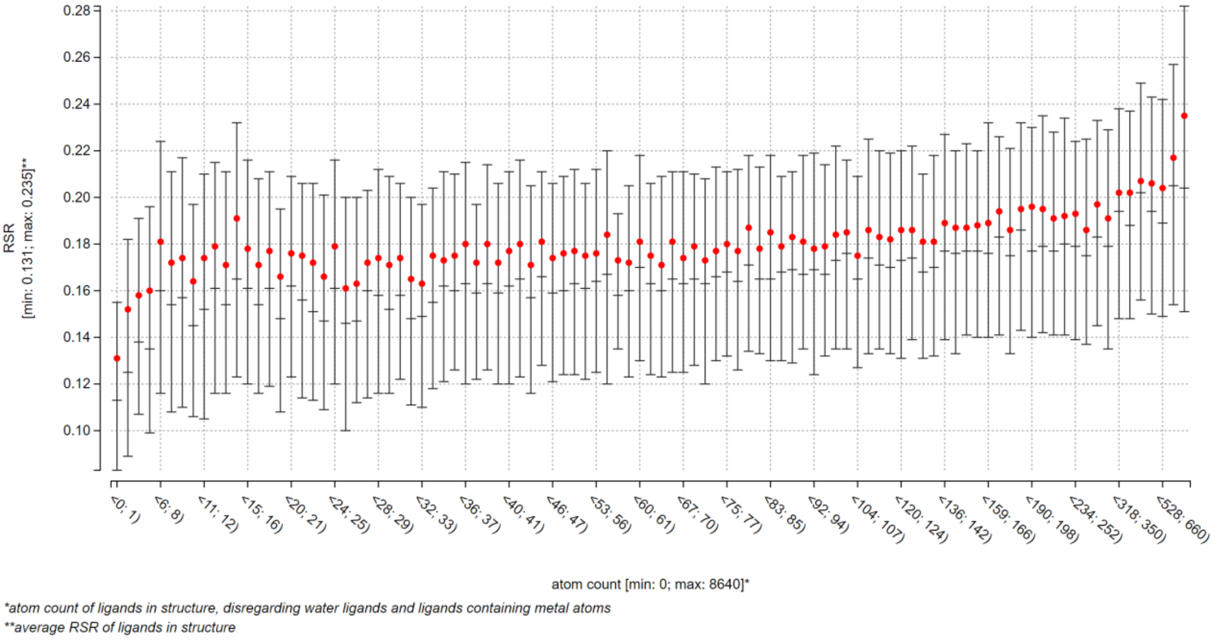

Figure S56. Relationship between factors *atom count of ligands in structure, disregarding water ligands and ligands containing metal atoms* and *average RSR of ligands in structure*. Lower values on Y axis represent higher quality, while higher values on X axis represent larger structures. Spearman's coefficient $\mathbf{R_s}$ **= 0.7821**.

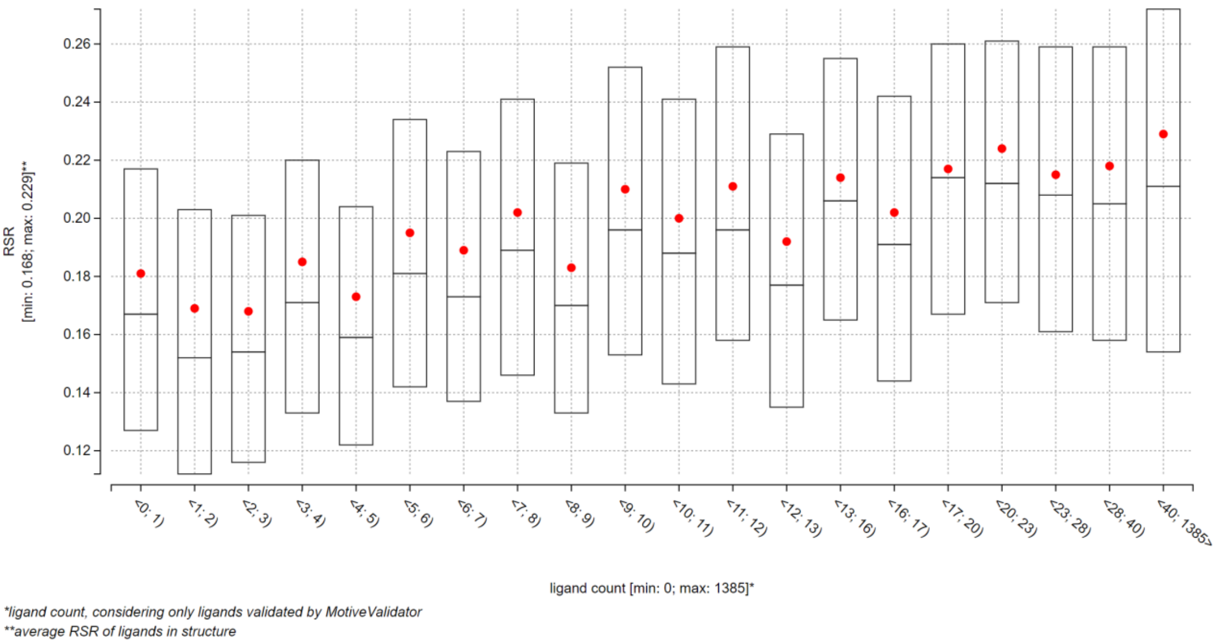

Figure S57. Relationship between factors *ligand count, considering only ligands validated by MotiveValidator* and *average RSR of ligands in structure*. Lower values on Y axis represent higher quality, while higher values on X axis represent larger structures. Spearman's coefficient $\mathbf{R_s}$ **=** **0.8720**.

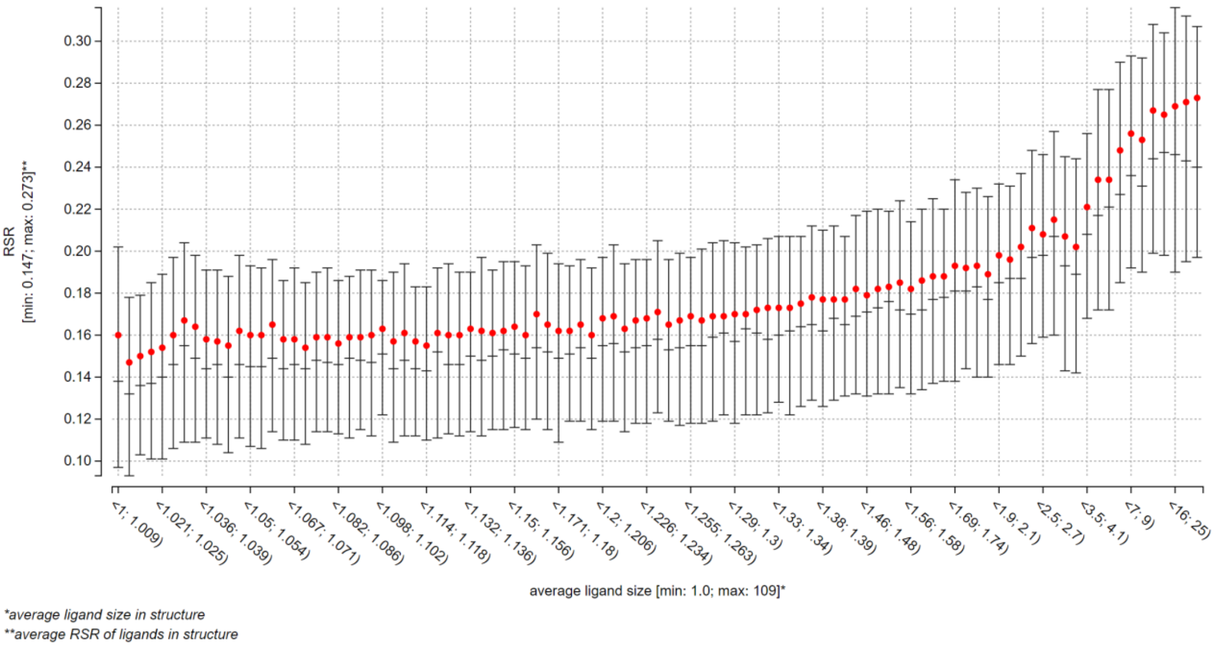

Figure S58. Relationship between factors *average ligand size in structure* and *average RSR of ligands in structure*. Lower values on Y axis represent higher quality, while higher values on X axis represent larger structures. Spearman's coefficient $\mathbf{R_s}$ **=** **0.9397**.

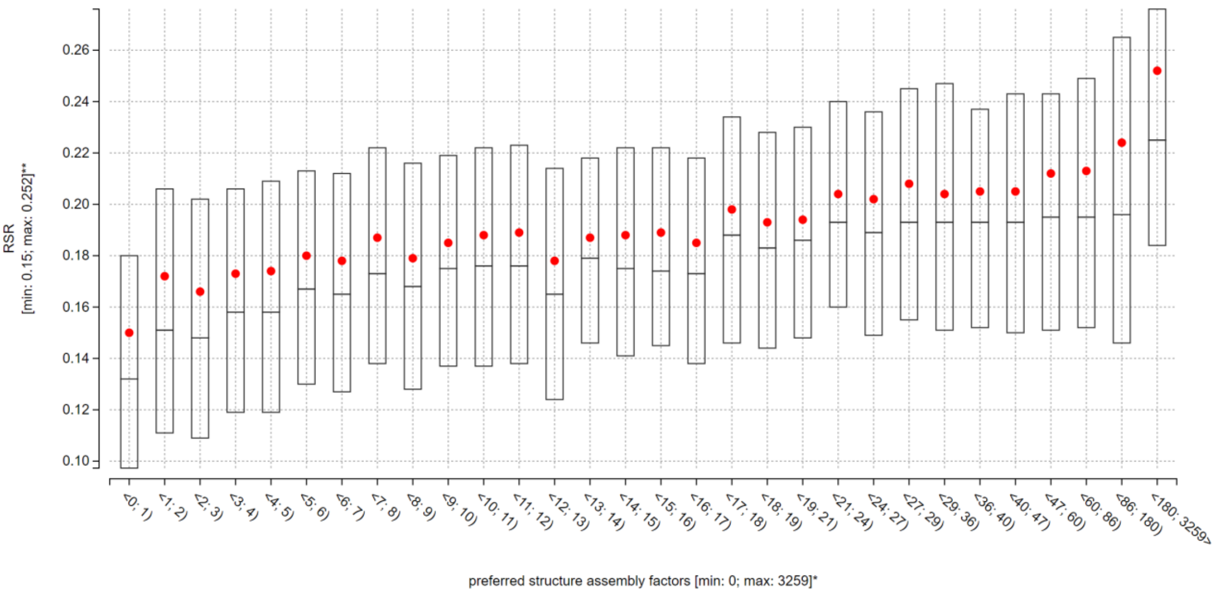

**total ligand molecule count in preferred structure assembly*
***average RSR of ligands in structure*

Figure S59. Relationship between factors *total ligand molecule count in preferred structure assembly* and *average RSR of ligands in structure*. Lower values on Y axis represent higher quality, while higher values on X axis represent larger structures. Spearman's coefficient **R_s = 0.9402**.

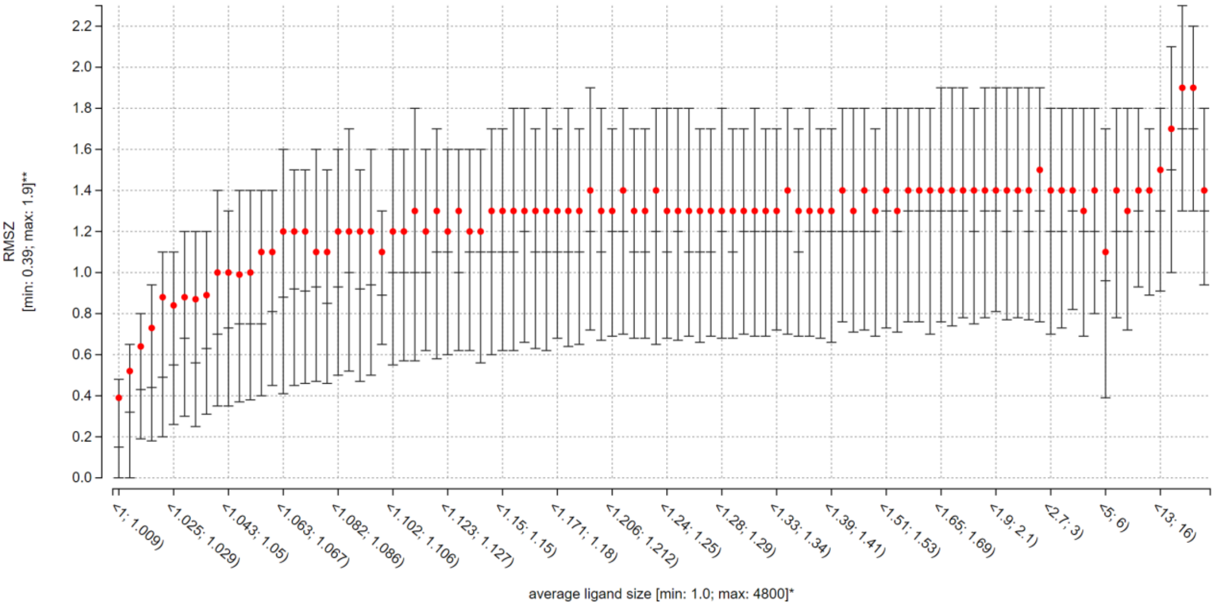

**average ligand size in structure*
***average RMSZ of bond angles in ligands*

Figure S60. Relationship between factors *average ligand size in structure* and *average RMSZ of bond angles in ligands*. Lower values on Y axis represent higher quality, while higher values on X axis represent larger structures. Spearman's coefficient **R_s = 0.8864**.

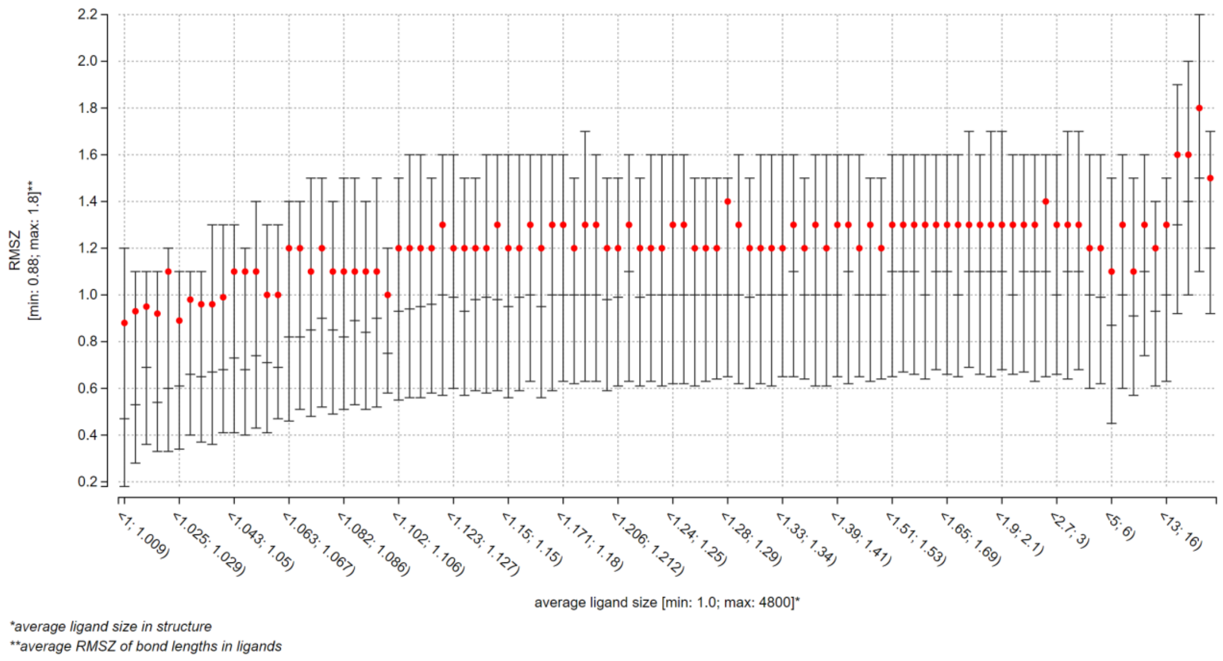

Figure S61. Relationship between factors *average ligand size in structure* and *average RMSZ of bond lengths in ligands*. Lower values on Y axis represent higher quality, while higher values on X axis represent larger structures. Spearman's coefficient $\mathbf{R_s}$ **=** **0.7469**.

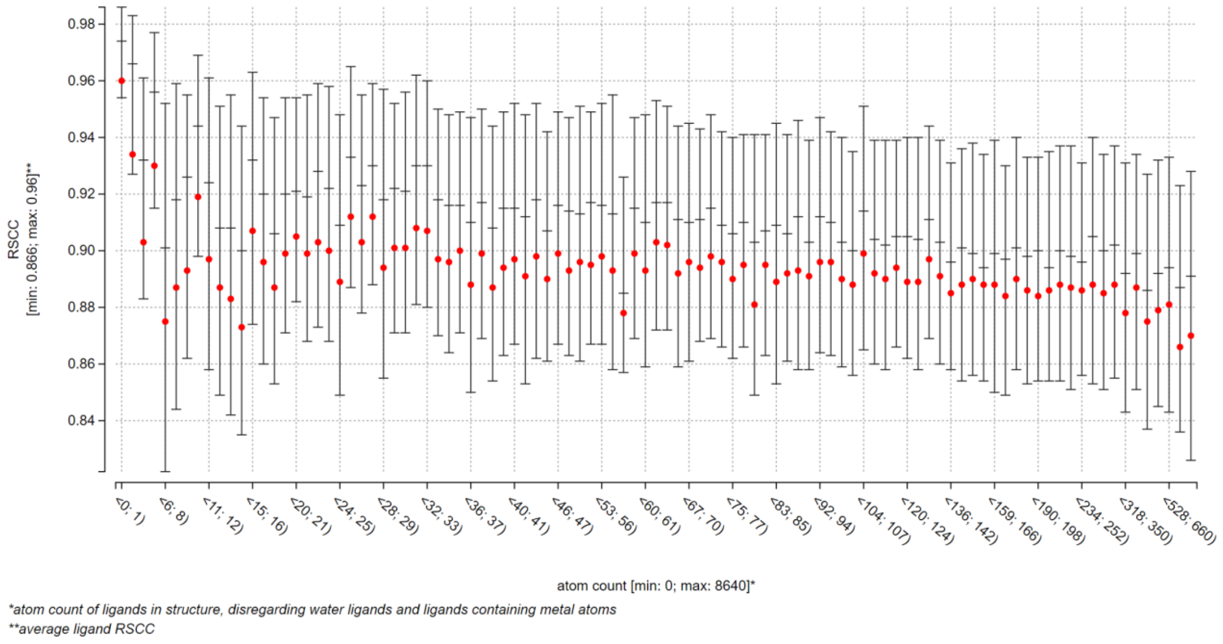

Figure S62. Relationship between factors *atom count of ligands in structure, disregarding water ligands and ligands containing metal atoms* and *average ligand RSCC*. Higher values on Y axis represent higher quality, while higher values on X axis represent larger structures. Spearman's coefficient $\mathbf{R_s}$ **=** **-0.5090**.

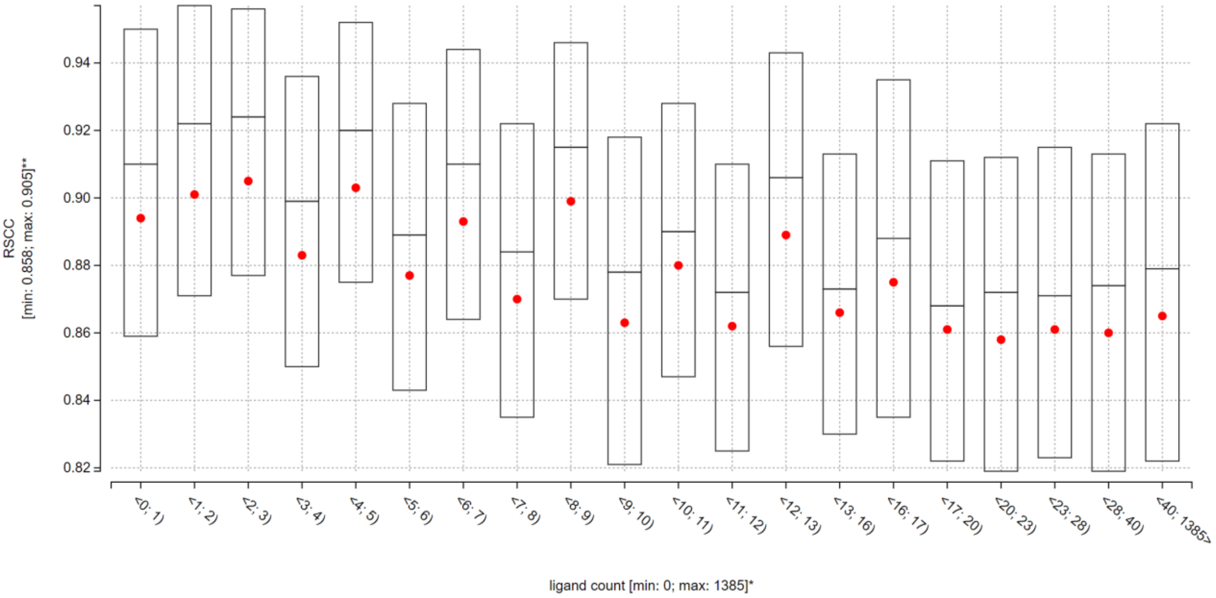

**ligand count, considering only ligands validated by MotiveValidator*
***average ligand RSCC*

Figure S63. Relationship between factors *ligand count, considering only ligands validated by MotiveValidator* and *average ligand RSCC*. Higher values on Y axis represent higher quality, while higher values on X axis represent larger structures. Spearman's coefficient **R_s = -0.8060**.

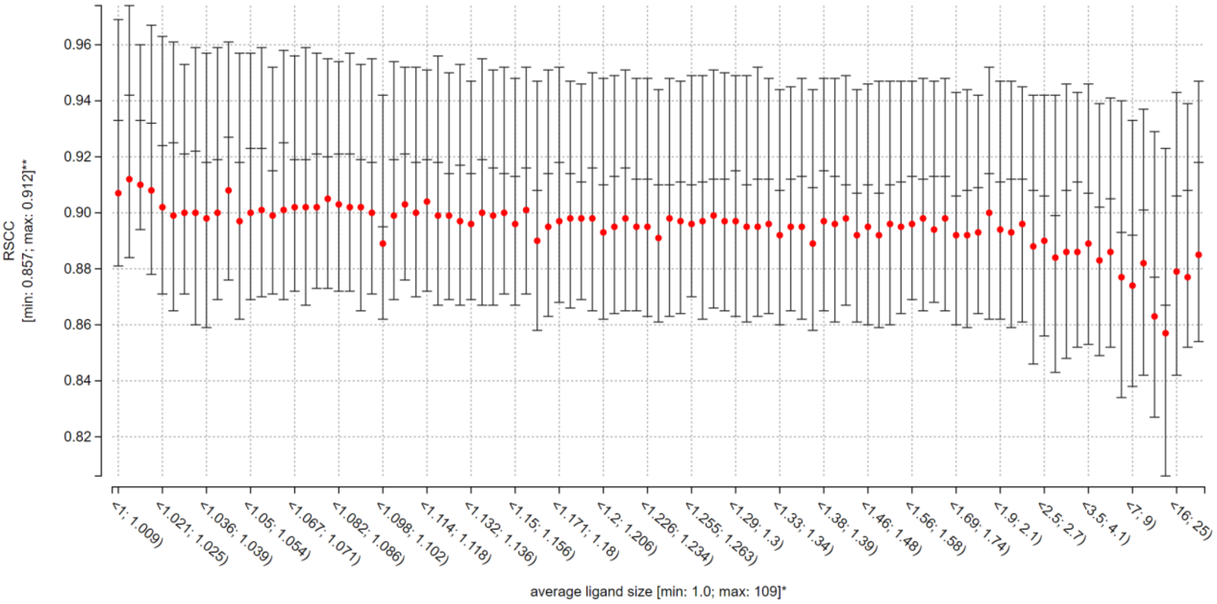

**average ligand size in structure*
***average ligand RSCC*

Figure S64. Relationship between factors *average ligand size in structure* and *average ligand RSCC*. Higher values on Y axis represent higher quality, while higher values on X axis represent larger structures. Spearman's coefficient **R_s = -0.7979**.

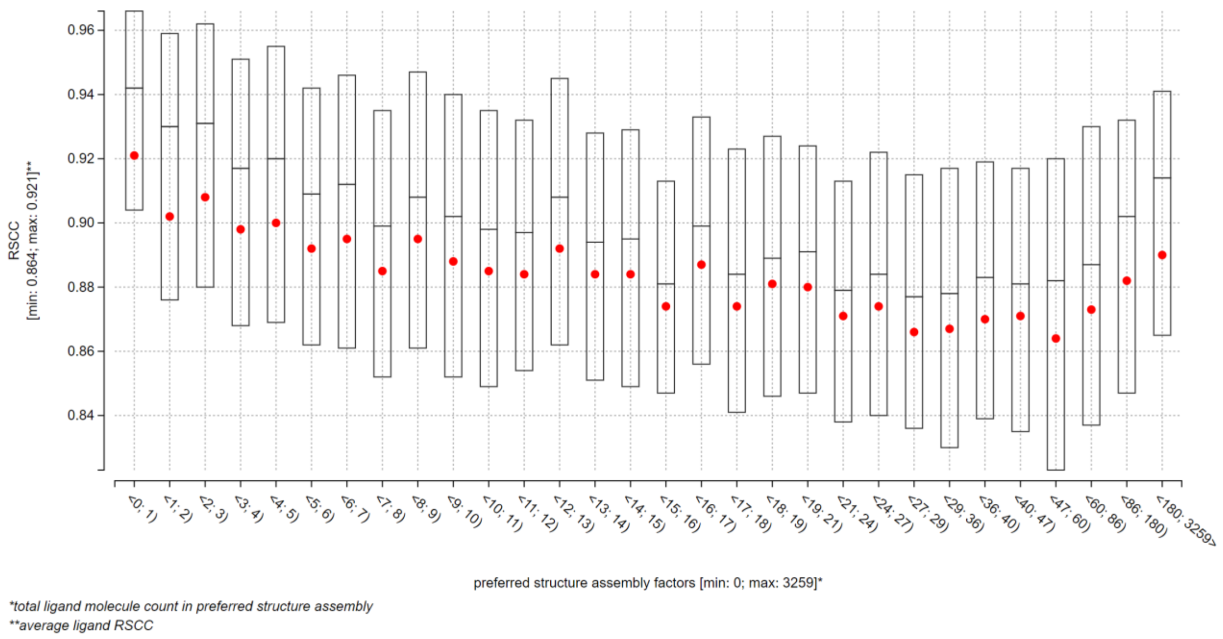

Figure S65. Relationship between factors *total ligand molecule count in preferred structure assembly* and *average ligand RSCC*. Higher values on Y axis represent higher quality, while higher values on X axis represent larger structures. Spearman's coefficient $\mathbf{R_s}$ **= -0.9554**.

Complete listing of factors and factor groups

88 factors have been considered in total throughout all versions of the dataset. Among them are metadata factors, structure quality factors, and ligand quality factors. Because of their total count, factors have been split into groups for user convenience.

Structure metadata factors

Atom count factor group contains factors that sum all atoms of parts of a PDB structure (structure itself, non-water ligands of a structure, water molecules of a structure). Parts of PDB structures considered are different for nearly every factor in this group (see the name of each factor). *Available in versions: current, yearly 2017, yearly 2016, yearly 2015.*

- Atom count of ligands in structure
- Atom count of ligands in structure, considering only ligands containing metal atoms
- Atom count of ligands in structure, considering only ligands validated by MotiveValidator[3]
- Atom count of ligands in structure, considering only ligands validated by MotiveValidator that contain metal atoms
- Atom count of ligands in structure, considering only ligands validated by MotiveValidator, without ligands containing metal atoms
- Atom count of ligands in structure, disregarding water ligands

[3] ValidatorDB (Sehnal *et al.*, 2015) contains validation results, computed by MotiveValidator (Svobodová Vařeková *et al.*, 2014), for only a subset of ligands stored in structures in the PDB. Specifically, it considers only ligands that are nontrivial (i.e. ligands that contain at least 7 atoms of element other than hydrogen). Derivatives of standard residues are omitted as well.

- Atom count of ligands in structure, disregarding water ligands and ligands containing metal atoms
- Atom count of ligands in structure, without ligands containing metal atoms
- Atom count (structure and ligand atoms)
- Atom count (structure and ligand atoms) on logarithmic scale
- Structure atom count (without ligand atoms)

Average ligand size factor group contains factors which values have been enumerated by a ratio of total number of ligand atoms in a PDB structure to the total number of ligands in a PDB structure. Set of considered ligands is different for each factor (see its name), but both parts of the fraction draw from the same set of ligands. *Available in versions: current, yearly 2017, yearly 2016, yearly 2015.*

- Average ligand size in structure
- Average ligand size in structure without ligands containing metal atoms
- Average ligand size in structure without ligands containing metal atoms, considering only ligands validated by MotiveValidator
- Average ligand size in structure without ligands containing metal atoms, disregarding water ligands
- Average ligand size in structure, considering only ligands validated by MotiveValidator
- Average ligand size in structure, considering only ligands validated by MotiveValidator and ligands containing metal atoms
- Average ligand size in structure, considering only ligands with metal atoms
- Average ligand size in structure, disregarding water ligands

Chiral carbon count in ligands factor represents sum of chiral carbon atoms across all ligands in a PDB structure. *Available in versions: current, yearly 2017, yearly 2016, yearly 2015.*

Ligand count factor group contains factors which values are sums of ligands in a PDB structure. Set of considered ligands is different for each factor (see its name). *Available in versions: current, yearly 2017, yearly 2016, yearly 2015*.

- Ligand count in structure
- Ligand count in structure without ligands containing metal atoms
- Ligand count in structure without ligands containing metal atoms, considering only ligands validated by MotiveValidator
- Ligand count in structure without ligands containing metal atoms, disregarding water ligands
- Ligand count in structure, considering only ligands validated by MotiveValidator and ligands containing metal atoms
- Ligand count in structure, disregarding water ligands
- Ligand count, considering only ligands validated by MotiveValidator
- Ligand count, considering only ligands with metal atoms, in structure

Molecular weight factor group contains factors which values are sums of weight of particular parts of a PDB structure. The unit used here is either one dalton [Da], or one kilodalton [kDa].

- Biopolymer weight in structure [kDa] *Available in versions: current, yearly 2017, yearly 2016, yearly 2015.*
- Ligand and water weight in structure [Da] *Available in versions: current.*
- Ligand and water weight in structure [kDa] *Available in versions: yearly 2017, yearly 2016, yearly 2015.*
- Ligand weight in structure [Da] *Available in versions: current.*
- Ligand weight in structure [kDa] *Available in versions: yearly 2017, yearly 2016, yearly 2015.*
- Total structure weight [kDa] *Available in versions: current, yearly 2017, yearly 2016, yearly 2015.*
- Water weight in structure [Da] *Available in versions: current.*
- Water weight in structure [kDa] *Available in versions: yearly 2017, yearly 2016, yearly 2015.*

Preferred structure assembly factor group contain factors that deal with metadata of preferred structure assembly[4] of each PDB structure. Explanation of structure assembly can be found here. Three types of factors can be found in this group. **Weight factors** enumerate total molecular weight of selected parts of the preferred structure assemblies. Weight unit used here is either one dalton [Da], or one kilodalton [kDa]. **Molecule count factors** enumerate total number of molecules of selected types that comprise the preferred structure assembly. **Flexibility ratio factor** shows how flexible are ligands that comprise the preferred structure assembly. It is enumerated as a ratio of rotatable bonds to all bonds of all ligands in the preferred structure assembly. The higher it is, the more flexible are the ligands.

- Total weight of preferred structure assembly [Da] *Available in versions: yearly 2017, yearly 2016.*
- Total weight of preferred structure assembly [kDa] *Available in versions: current.*
- Total biopolymer weight of preferred structure assembly [Da] *Available in versions: yearly 2017, yearly 2016.*
- Total biopolymer weight of preferred structure assembly [kDa] *Available in versions: current.*
- Total ligand weight of preferred structure assembly [Da] *Available in versions: current, yearly 2017, yearly 2016.*
- Total water weight of preferred structure assembly [Da] *Available in versions: current, yearly 2017, yearly 2016.*
- Total biopolymer unit count of preferred structure assembly *Available in versions: current, yearly 2017, yearly 2016.*
- Total ligand molecule count in preferred structure assembly *Available in versions: current, yearly 2017, yearly 2016.*
- Total water molecule count of preferred structure assembly *Available in versions: current, yearly 2017, yearly 2016.*
- Number of unique biopolymer units in preferred structure assembly *Available in versions: current, yearly 2017, yearly 2016.*
- Number of unique ligand molecules in preferred structure assembly *Available in versions: current, yearly 2017, yearly 2016.*

[4] Assembly is an arrangement of units that form a biomacromolecular complex together. Some assemblies consist of only one chain and no ligands, while other assemblies are made of hundreds of biopolymers and ligands. Each entry of the PDB database has exactly one preferred assembly.

- Flexibility ratio of ligands in preferred structure assembly *Available in versions: current, yearly 2017, yearly 2016.*

Ratio of single bonds in ligands factor value is a fraction of sum of all sigma bonds of all ligands in a PDB structure to the sum of all bonds in all ligands in said PDB structure. *Available in versions: current, yearly 2017, yearly 2016, yearly 2015.*

Residue count factor group contains factors which values represent the total number of standard residues in a PDB structure. Some factors in this group add number of relevant ligands in a PDB structure to its value (what ligands are relevant for each factor is clear from its name). *Available in versions: current, yearly 2017, yearly 2016, yearly 2015.*

- Residue and ligand count in structure
- Residue and ligand count in structure, considering only ligands validated by MotiveValidator
- Residue and ligand count in structure, disregarding water ligands
- Residue count in structure

Structure resolution factor represents highest resolution of a PDB structure in Ångströms. Formally, it is the smallest value of the interplanar spacings for the reflection data to be used in the refinements[5]. *Available in versions: current, yearly 2017, yearly 2016, yearly 2015.*

Year of release factor represents the year when a PDB structure was published in the PDB database. *Available in versions: current, yearly 2017, yearly 2016, yearly 2015.*

Structure quality factors

Average RSR[6] of residues in structure factor represents the average deviation size of standard residue structure in real space from its atomic model[7]. *Available in versions: current, yearly 2017, yearly 2016, yearly 2015.*

Clashscore factor represents the amount of atom clashes (i.e. pairs of atoms that are unusually close to each other) in structure. Formally, it is expressed as number of clashes per thousand atoms of a PDB structure[8]. Ligand atoms are considered as well as atoms of standard residues. Two variants of the clashscore factor have been considered. *Available in versions: current, yearly 2017, yearly 2016, yearly 2015.*

- **Clashscore structure quality factor:** Each PDB structure is represented in this factor by its clashscore structure quality metric.
- **Clashscore structure quality factor - percentile version:** This factor shows quality of a PDB structure in relation to the rest of structures in the PDB database. Its value ranges from 0 to 100. The higher is its value, the larger portion of structures in the PDB database has lower quality than this structure.

Ramachandran outliers factor represents percentage of standard residues in a PDB structure that are identified as Ramachandran outliers. A standard residue is identified as a Ramachandran

[5] http://mmcif.wwpdb.org/dictionaries/mmcif_pdbx_v40.dic/Items/_refine.ls_d_res_high.html
[6] real-space R-value
[7] http://www.wwpdb.org/validation/legacy/XrayValidationReportHelp#overall_quality
[8] http://www.wwpdb.org/validation/legacy/XrayValidationReportHelp#close_contacts

outlier if the combination of backbone ϕ-ψ torsion angle values is unusual[9]. Two variants of the Ramachandran outliers factor have been considered. *Available in versions: current, yearly 2017, yearly 2016, yearly 2015.*

- **Ramachandran outliers structure quality factor:** Each PDB structure is represented in this factor by its Ramachandran outliers structure quality metric.
- **Ramachandran outliers structure quality factor - percentile version:** This factor shows quality of a PDB structure in relation to the rest of structures in the PDB database. Its value ranges from 0 to 100. The higher is its value, the larger portion of structures in the PDB database has lower quality than this structure.

R_{free} factor is a refinement statistic of a PDB structure model. It measures similarity between observed structure factor[10] amplitudes and those calculated from the model while using reflections that were not used during model refinement. Lower value is usually better[11]. Too low value may point to overfitting of the model though. *Available in versions: current, yearly 2017, yearly 2016, yearly 2015.*

RMSZ[12] factor group contains factors that quantify deviation of bond angles and bond lengths in standard residues of a PDB structure. It is calculated for individual standard residues, then averaged for each chain, and then - in the case of two factors from this factor group - averaged over the whole structure. Scores of factors from this factor group are expected to lie between 0 and 1[13]. *Available in versions: current, yearly 2017, yearly 2016, yearly 2015.*

- Average RMSZ of bond angles in residues
- Average RMSZ of bond lengths in residues
- Highest RMSZ of bond angles in residues of a chain in structure
- Highest RMSZ of bond lengths in residues of a chain in structure

RSCC[14] factor group contains factors that determine how well the calculated electron density map matches the electron density map that has been computed from experimental data. They are alternatives to the RSR factor family. Standard residue qualifies as an outlier if its RSCC value is below 0.8[15]. Two variants of RSCC factors have been considered. *Available in versions: current, yearly 2017, yearly 2016.*

- **Average residue RSCC:** This factor represents each PDB structure by a RSCC value that has been averaged through all of its standard residues.
- **Ratio of RSCC outliers among residues:** This factor is valued for each PDB structure as a ratio of the number of standard residues, classified as outliers, to the total number of standard residues that comprise the structure.

---

[9] http://www.wwpdb.org/validation/legacy/XrayValidationReportHelp#torsion_angles
[10] this term has different meaning in X-ray crystallography than in the analysis described in the article
[11] http://www.wwpdb.org/validation/legacy/XrayValidationReportHelp#data_stats
[12] root-mean-square value of the Z-scores
[13] http://www.wwpdb.org/validation/legacy/XrayValidationReportHelp#standard_geometry
[14] real-space correlation coefficient
[15] http://www.wwpdb.org/validation/2016/XrayValidationReportHelp#fit_nonstandard_and_ligands

RSRZ[16] factor group contains factors that quantify percentage of standard residues in a PDB structure that qualify as real-space R-value outliers (RSRZ). RSRZ is a measure of how much does the fit quality between the atomic model of a standard residue and its data in real space deviate from expected value, as assessed by the RSR valdiation metric. Standard residue qualifies as an outlier if its RSRZ value is above 2[17]. Two variants of the RSRZ outlier percentage factor have been considered. *Available in versions: current, yearly 2017, yearly 2016, yearly 2015.*

- **RSRZ outlier percentage in residues:** Each PDB structure is represented in this factor by its RSRZ outlier percentage structure quality metric.
- **RSRZ outlier count in residues - percentile version:** This factor shows quality of a PDB structure in relation to the rest of structures in the PDB database. Its value ranges from 0 to 100. The higher is its value, the larger portion of structures in the PDB database has lower quality than this structure.

R_{value} factor is a refinement statistic of a PDB structure model. It measures similarity between observed structure factor[18] amplitudes and those calculated from the model. Lower value is usually better[19]. Too low value may point to overfitting of the model though. *Available in versions: current, yearly 2017, yearly 2016, yearly 2015.*

Sidechain outliers factor represents percentage of standard residues in a PDB structure with sidechains which torsion angle combination is considered to be an outlier, i.e. is not a preferred combination[20]. Two variants of the Sidechain outliers factor have been considered. *Available in versions: current, yearly 2017, yearly 2016, yearly 2015.*

- **Sidechain outliers structure quality factor:** Each PDB structure is represented in this factor by its Sidechain outliers percentage structure quality metric.
- **Sidechain outliers structure quality factor - percentile version:** This factor shows quality of a PDB structure in relation to the rest of structures in the PDB database. Its value ranges from 0 to 100. The higher is its value, the larger portion of structures in the PDB database has lower quality than this structure.

Ligand quality factors

Average RSR of ligands in structure factor represents the average deviation size of ligand molecule structure in real space from its atomic model[21]. *Available in versions: current, yearly 2017, yearly 2016, yearly 2015.*

Chiral quality of ligands in structure factor group contains factors that quantify the relative amount of chiral carbon atoms with incorrect configuration. *Available in versions: current, yearly 2017, yearly 2016, yearly 2015.*

[16] real-space R-value Z-score
[17] http://www.wwpdb.org/validation/legacy/XrayValidationReportHelp#overall_quality
[18] this term has different meaning in X-ray crystallography than in the analysis described in the article
[19] http://www.wwpdb.org/validation/legacy/XrayValidationReportHelp#data_stats
[20] http://www.wwpdb.org/validation/legacy/XrayValidationReportHelp#overall_quality
[21] http://www.wwpdb.org/validation/legacy/XrayValidationReportHelp#fit_nonstandard_and_ligands

- **Ratio of ligands with problems in carbon chirality:** Value of this factor is a ratio of ligands that contain at least one chiral carbon atom with incorrect configuration to the total number of ligands in a PDB structure.
- **Relative ratio of atoms involved in carbon chirality problems:** Value of this factor is a ratio of sum of chiral carbon atoms with incorrect configuration across all ligands of a PDB structure to the total number of chiral carbon atoms across all ligands in the same PDB structure.
- **Relative ratio of atoms involved in carbon chirality problems - binary version:** Value of this factor is 1 if a ligand in a PDB structure contains at least one chiral carbon atom with incorrect configuration. Otherwise, it is 0.

Combined quality of ligands in structure factor group contains factors that quantify both topological and chiral problems of ligands in a PDB structure (see links for details). *Available in versions: current, yearly 2017, yearly 2016, yearly 2015.*

- **Ratio of ligands without problems in topology and carbon chirality:** Value of this factor is a ratio of ligands that have neither topological problems, nor chiral problems to the total number of ligands in a PDB structure.
- **Ratio of ligands without problems in topology and carbon chirality - binary version:** Value of this factor is 1 if all ligands of a PDB structure are free from topological and chiral problems. Otherwise, it is 0.
- **Relative ratio of atoms involved in topological problems and carbon atoms in carbon chirality problems:** Value of this factor is a sum of values of factors *Relative ratio of atoms involved in topological problems* and *Relative ratio of atoms involved in carbon chirality problems*.
- **Relative ratio of atoms involved in topological problems and carbon atoms in carbon chirality problems - binary version:** Value of this factor is 1 if the value of factor *Relative ratio of atoms involved in topological problems and carbon atoms in carbon chirality problems* is nonzero. Otherwise, it is 0.

LLDF[22] factor group quantifies the Local Ligand Density Values (LLDF) of ligands in a PDB structure. LLDF is a Z-score[23] computed as a statistical comparison of RSR of a ligand to the RSR values of neighboring standard residues that are present within 5 Å of the ligand in question. If there are no standard residues within 5 Å of a ligand, LLDF cannot be computed for such ligand. A ligand is considered to be a negative quality outlier if its LLDF value is greater than 2[24]. Two variants (plus two more) of LLDF factors have been considered.

- **Average ligand LLDF:** This factor represents each PDB structure by a LLDF value that has been averaged through all of its ligands. *Available in versions: yearly 2017, yearly 2016.*

---

[22] local ligand density fit

[23] Z-score is defined as the difference between an observed value and either expected or average value, divided by the standard deviations of either the expected or the average value (http://stattrek.com/statistics/dictionary.aspx?definition=z%20score). Z-scores are used in PDB validation reports.

[24] http://www.wwpdb.org/validation/legacy/XrayValidationReportHelp#fit_nonstandard_and_ligands

- **Ratio of LLDF outliers among ligands:** This factor is valued for each PDB structure as a ratio of the number of ligands, classified as outliers, to the total number of ligands that comprise the structure. *Available in versions: yearly 2017, yearly 2016, yearly 2015.*
- **Average ligand LLDF of small ligands:** This factor represents each PDB structure by a LLDF value that has been averaged through all of its ligands. Only small ligands (with 10 heavy atoms or less) have been considered for this factor. *Available in versions: yearly 2017, yearly 2016.*
- **Average ligand LLDF of big ligands:** This factor represents each PDB structure by a LLDF value that has been averaged through all of its ligands. Only big ligands (with 11 heavy atoms or more) have been considered for this factor. *Available in versions: yearly 2017, yearly 2016.*

RMSZ factor group contains factors that quantify deviation of bond angles and bond lengths in ligands of a PDB structure. It is calculated for individual ligands, then averaged over the whole molecule. Scores of factors from this factor group are expected to lie between 0 and 1[25]. *Available in versions: current, yearly 2017, yearly 2016, yearly 2015.*

- Average RMSZ of bond angles in ligands
- Average RMSZ of bond lengths in ligands

RSCC factor group contains factors that determine how well the calculated electron density map matches the electron density map that has been computed from experimental data. They are alternatives to the RSR factor family. Ligand qualifies as an outlier if its RSCC value is below 0.8[26]. Two variants (plus two more) of RSCC factors have been considered. *Available in versions: current, yearly 2017, yearly 2016.*

- **Average ligand RSCC:** This factor represents each PDB structure by a RSCC value that has been averaged through all of its ligands.
- **Ratio of RSCC outliers among ligands:** This factor is valued for each PDB structure as a ratio of the number of ligands, classified as outliers, to the total number of ligands that comprise the structure.
- **Average ligand RSCC of small ligands:** This factor represents each PDB structure by a RSCC value that has been averaged through all of its ligands. Only small ligands (with 10 heavy atoms or less) have been considered for this factor.
- **Average ligand RSCC of big ligands:** This factor represents each PDB structure by a RSCC value that has been averaged through all of its ligands. Only big ligands (with 11 heavy atoms or more) have been considered for this factor.

Topological quality of ligands in structure factor group contains factors that quantify the relative amount of atoms that are either missing or redundant in ligands of a PDB structure. Atoms that are not present in a ligand for chemically valid reasons (e.g. atoms that were lost when a covalent bond was formed) are not quantified by factors from this factor group. *Available in versions: current, yearly 2017, yearly 2016, yearly 2015.*

[25] http://www.wwpdb.org/validation/legacy/XrayValidationReportHelp#nonstandard_residues_and_ligands
[26] http://www.wwpdb.org/validation/2016/XrayValidationReportHelp#fit_nonstandard_and_ligands

- **Ratio of ligands with topological problems:** Value of this factor is a ratio of ligands that contain, or are missing, at least one topologically problematic atom to the total number of ligands in a PDB structure.
- **Relative ratio of atoms involved in topological problems:** Value of this factor is a ratio of sum of topologically problematic atoms across all ligands of a PDB structure to the total number of atoms across all ligands in the same PDB structure.
- **Relative ratio of atoms involved in topological problems - binary version:** Value of this factor is 1 if a ligand in a PDB structure contains, or is missing, at least one topologically problematic atom. Otherwise, it is 0.

Version overview

Below is the outline of versions that are available in ValTrendsDB. Please note that yearly versions are named by the last year from which all PDB entries are included in the analysis. They are not named by the year when the complete dataset for a version was obtained. The reason for the discrepancy between the name of version and the year when it was obtained is that the first update of the PDB database in a year contains the rest of PDB entries from the previous year.

current yyyy-mm-dd

The current version is based on the latest version of source data available, and is updated weekly. The date of origin of this version is included in its name. Unlike yearly versions, the current version was not processed by a statistician. Therefore, the Spearman coefficients are not available for this version. All other outputs of ValTrendsDB are, however, provided by this version as well.

An additional difference from the yearly 2017 version is that all LLDF ligand quality factors were omitted, since PDB Validation Reports no longer offer them.

yearly 2017

The yearly 2017 version is based on source data from the 3rd January 2018. It is the first version to include all PDB entries into the analysis, not just structures acquired using X-ray crystallography.

yearly 2016

The yearly 2016 version is based on source data from the 18th January 2017. It contains 19 new factors (e.g., the assembly factors, the RSCC factors for standard residues and ligands) and trends among 533 new factor pairs.

yearly 2015

The yearly 2015 version is based on source data from the 17th February 2016. It is the first version of dataset presented in ValTrendsDB.

References

Brunger,A.T. (1992) Free R value: a novel statistical quantity for assessing the accuracy of crystal structures. *Nature*, **355**, 472–475.

Chen,V.B. *et al.* (2010) MolProbity: all-atom structure validation for macromolecular crystallography. *Acta Crystallogr. D. Biol. Crystallogr.*, **66**, 12–21.

Gore,S. *et al.* (2017) Validation of Structures in the Protein Data Bank. *Structure*, **25**, 1916–1927.

Jones,T.A. *et al.* (1991) Improved methods for building protein models in electron density maps and the location of errors in these models. *Acta Crystallogr. Sect. A*, **47**, 110–119.

Kleywegt,G.J. *et al.* (2004) The Uppsala Electron-Density Server. *Acta Crystallogr. Sect. D Biol. Crystallogr.*, **60**, 2240–2249.

Sehnal,D. *et al.* (2015) ValidatorDB: database of up-to-date validation results for ligands and non-standard residues from the Protein Data Bank. *Nucleic Acids Res. Database issue*, **43**, D369–D375.

Svobodová Vařeková,R. *et al.* (2014) MotiveValidator: interactive web-based validation of ligand and residue structure in biomolecular complexes. *Nucleic acids Res. Web Serv. issue*, **42**, W227–W233.

Yang,H. *et al.* (2016) DCC: a Swiss army knife for structure factor analysis and validation. *J. Appl. Crystallogr.*, **49**, 1081–1084.
